# Cellular Morphological Profiling of PROTAC Degraders of the m6A RNA Methyltransferase METTL3

**DOI:** 10.1021/acsbiomedchemau.6c00136

**Published:** 2026-10-06

**Authors:** Mao Jiang, Chukwudi T. Ugwu, Laura Wortmann, Jeffrey L. Schloßhauer, Christian Begemann, Sonja Sievers, Katarzyna W. Kliza, Peng Wu

**Affiliations:** † Chemical Genomics Centre, Max Planck Institute of Molecular Physiology, Dortmund 44227, Germany; ‡ Department of Chemical Biology, Max Planck Institute of Molecular Physiology, Dortmund 44227, Germany; § Faculty of Chemistry and Chemical Biology, TU Dortmund University, Dortmund 44227, Germany; ∥ Max Planck Institute of Molecular Physiology, Dortmund 44227, Germany; ⊥ Compound Management and Screening Center, Dortmund 44227, Germany

**Keywords:** RNA modifications, RNA methyltransferase METTL3, PROTAC degrader, anticancer small molecules, cell painting assay

## Abstract

Methylation of N6-adenosine is the most abundant RNA modification, which is mainly installed on mRNA by the methyltransferase METTL3–METTL14 complex. Dysregulation of the catalytic activity of METTL3 has been associated with many human diseases, especially in acute myeloid leukemia and other cancers. Therefore, both monovalent small-molecule inhibitors and bivalent heterobifunctional proteolysis-targeting chimera (PROTAC) degraders have been pursued to address METTL3 for therapeutic development. In this study, we report the structure-based design and synthesis of new METTL3-targeting PROTACs, as well as the first application of unbiased morphological profiling of the obtained METTL3 PROTACs in the cell painting assay to obtain phenotypic characterization data for the further development of METTL3 PROTAC degraders.

## Graphical abstract

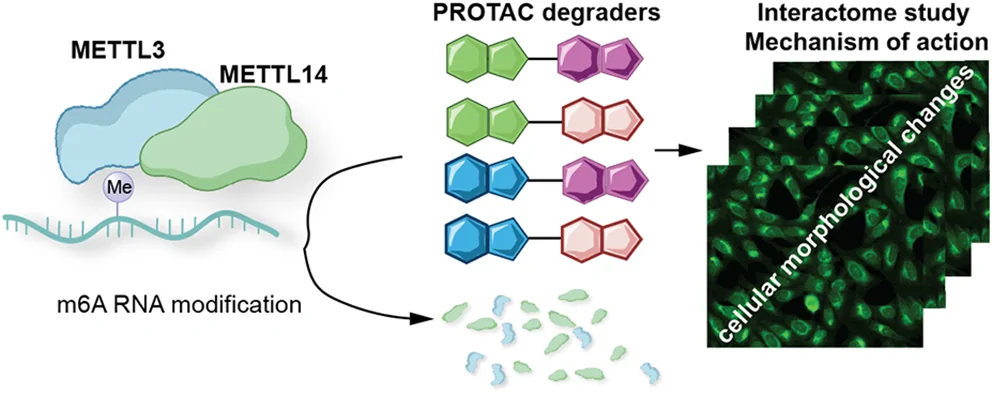

## Introduction

RNA modification is a critical layer of post-transcriptional regulation, impacting various cellular activities at both the transcriptional and translational levels.^1,2^ The N6-methyladenosine (m6A) RNA modification is the most common mRNA modification in mammalian cells. The reversible m6A modification is coordinated by a suite of RNA-binding and -modifying proteins that function as methyltransferases (writers), demethylases (erasers), and recognition proteins (readers) (Figure 1A).^3^ The installation of m6A is achieved by methyltransferases such as the methyltransferase-like 3­(METTL3)–methyltransferase-like 14 (METTL14) complex and METTL16, with the former being the primary m6A writer for the consensus GGACU sequence that is enriched at the 3′-untranslated regions. METTL3 is the catalytic core of the METTL3–METTL14 complex, coupling with other cofactors.^4^ METTL14 contributes to sustaining the complex integrity and facilitating RNA substrate binding, with S-adenosyl-l-methionine (SAM) being the cosubstrate and the methyl donor.

**Figure 1:**
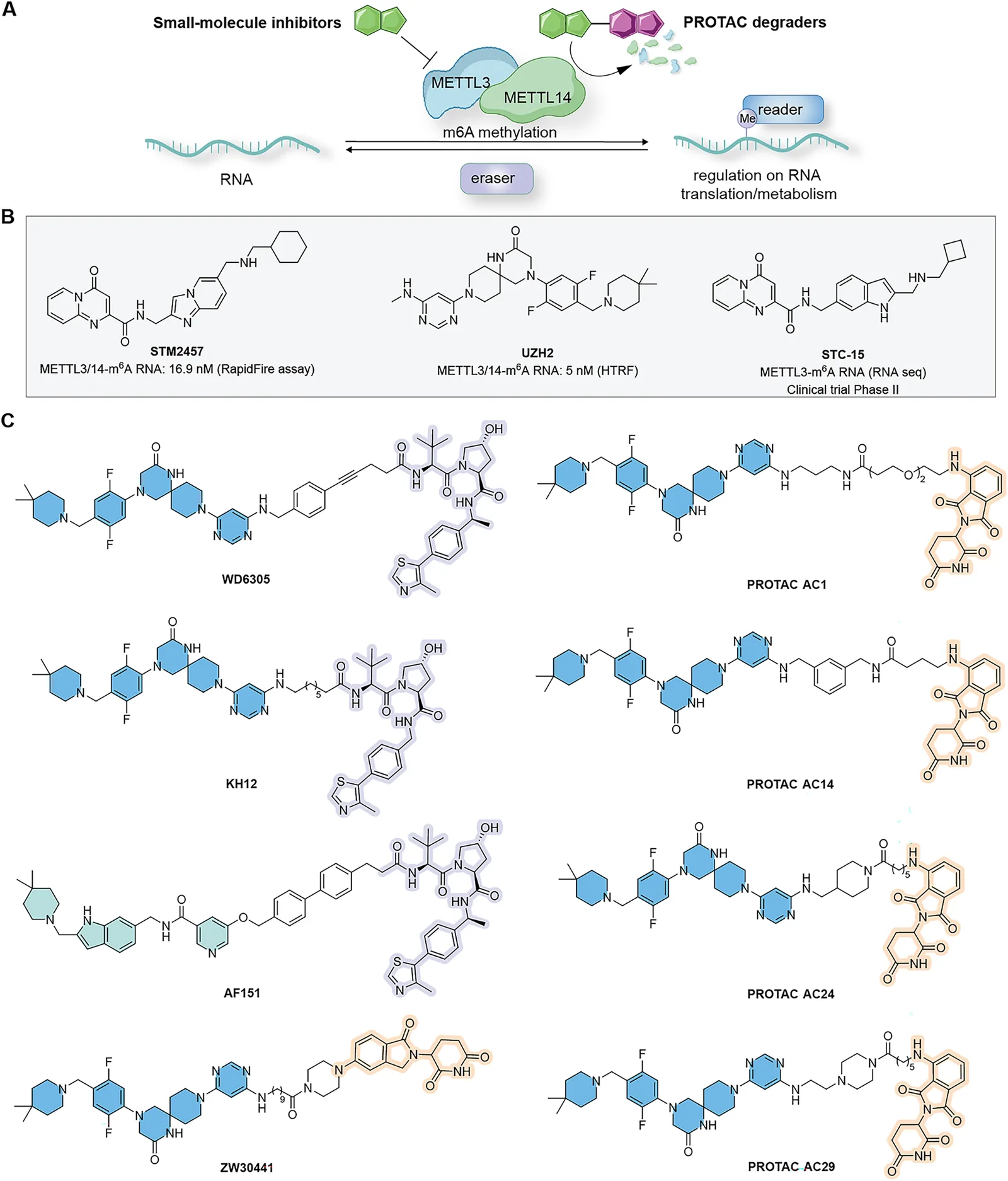
Targeting the m6A RNA methyltransferase METTL3 with small molecules and PROTAC degraders. (A) Small-molecule-based strategies to target the m6A RNA-modifying protein METTL3 via monovalent inhibitors and bivalent bifunctional PROTAC degraders. (B) Representative examples of reported small-molecule inhibitors of METTL3. (C) Examples of reported METTL3-targeting PROTAC degraders. The majority of the reported PROTACs consist of UZH2-based analogues as the METTL3-binding component (shown in blue-filled ring structures), conjugated with small-molecule ligands recruiting either the VHL or cereblon E3 ligase (the VHL ligand is shown in gray and the cereblon ligand shown in orange).

Dysregulation of the dynamic m6A RNA modification is closely associated with disease development in humans, including neurological diseases, cardiovascular diseases, viral infections, and especially cancers.^5^ An increasing number of studies have revealed the prominent role of METTL3 in driving tumorigenesis, metastasis, and tumor growth. METTL3 is aberrantly highly expressed in many cancer types and drives translation of oncogenes in acute myeloid leukemia (AML),^6^ lung cancers,^7^ and osteosarcoma.^8^ Additionally, high expression of METTL14 is observed in AML cells. It is noteworthy that, despite the close association with cancer development, METTL3 is reported to function either as an oncogene or a tumor suppressor in different cancer types.^9^

Overall, given the importance of METTL3 in tumorigenesis and cancer progression, the development of small-molecule-based strategies to target METTL3 (and METTL14) is emerging as a promising anticancer research field (Figure 1A).^10^ So far, such small-molecule-based strategies include mainly two classes: monovalent small-molecule inhibitors and bivalent heterobifunctional molecules in the format of proteolysis-targeting chimera (PROTAC). Albeit there is a high cellular concentration of SAM, SAM-competitive small-molecule METTL3 inhibitors with high selectivity and potency have been reported, such as STM2457,^11^ UZH2,^12^ STM3006,^13^ and EP652.^14^ Small-molecule inhibitors have been shown to increase differentiation, induce apoptosis, and reduce overall AML growth. STC-15,^15^ which is a structural analogue of STM2457, is among the most advanced developed molecules in the field and is currently being evaluated in a clinical trial for patients with advanced cancers and sarcomas (NCT06975293) (Figure 1B).

Apart from small-molecule inhibitors, METTL3-degrading PROTACs have been emerging chemical modalities in the field since 2024, when this manuscript was initially being prepared. PROTACs are heterobifunctional molecules that are composed of an inhibitor/binder to the protein of interest, a ligand recruiting an E3 ubiquitin ligase, and a linker. Different from monovalent small-molecule inhibitors, PROTACs disrupt both the enzymatic and scaffolding functions of proteins via a catalytic degradation mechanism. The majority of the reported METTL3-PROTACs were built from the UZH2-based METTL3-binding component, such as WD6305,^16^ AC series PROTACs,^17^ KH12,^18^ and ZW30441.^19^ Either cereblon or VHL-binding ligands were used for E3 ligase recruitment. The resulting PROTACs have been shown to degrade METTL3 and METTL14 via induced ubiquitination and a subsequent proteasome-mediated system. Non-UZH2-based METTL3 PROTACs have also been reported, such as AF151 and associated indole-nicotinamide analogues.^20^ These studies convincingly demonstrated the feasibility of applying the PROTAC-based degrading strategy to target METTL3 in AML and other cancer cells. Recently, both reported data and our in-house results have demonstrated that the cell painting assay (CPA) is a powerful, unbiased phenotypic method to characterize new bifunctional modalities, including PROTACs^21,22^ and ribonuclease-targeting chimeric small molecules (RIBOTACs),^23^ while no such phenotypic method has been applied to evaluate the METTL3 PROTACs.

Associated with our recent studies in developing small molecules targeting m6A RNA-binding and -modifying proteins,^24,25^ in this study, we report the structure-based design and synthesis of new METTL3-targeting PROTACs to diversify the METTL3-degrading repertoire. Also, we report here for the first time the characterization of METTL3 PROTACs via unbiased morphological profiling in the cell painting assay to provide an alternative evaluation approach for the further development of METTL3 PROTACs (Figure 2).

**Figure 2:**
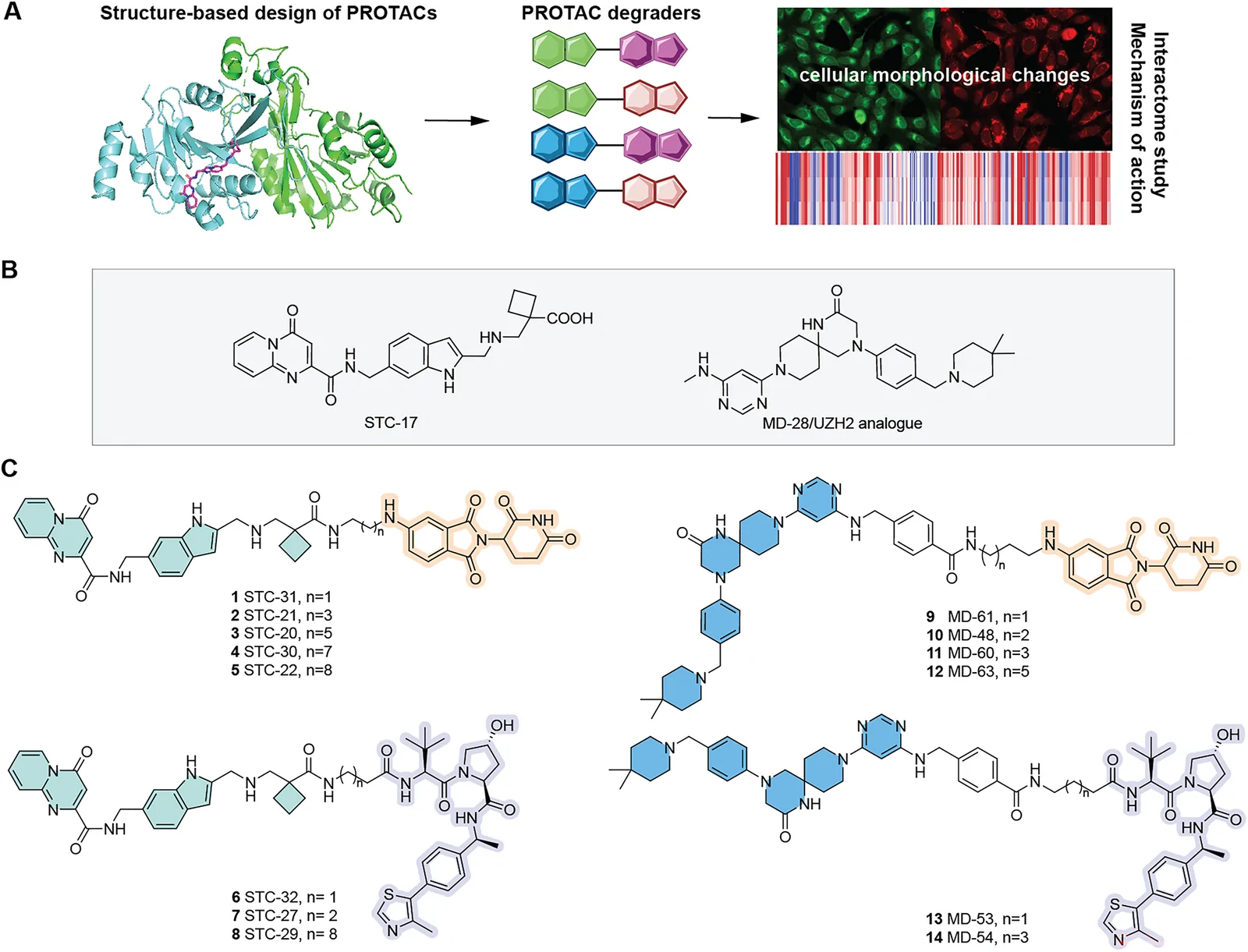
Overview of the workflow of this study in profiling the cellular morphological changes in cancer cells with the in-house designed and synthesized METTL3 PROTACs. (A) Structure-based design and synthesis of METTL3-targeting PROTAC degraders, which were subject to unbiased cellular morphological profiling in the cell painting assay to obtain phenotypic characterization data. The complex structure shown on the left is the METTL3-METTL14 complex with SAM (PDB ID: 5IL1). METTL3 is shown in turquoise, METTL14 in green, and SAM with a pink backbone. The PROTAC degrader elements shown in the middle have the following color codes: green structures indicate STC-17 analogues, blue structures indicate UZH2 analogue, pink structures indicate VHL-binding ligands, and orange structures indicate cereblon-binding ligands. (B) Structures of STC-17 and MD-28 (UZH2 analogue) that are used as the building components to bind METTL3 for the assembly of the PROTACs. (C) The designed PROTACs can be classified into four subclasses based on the different METTL3-binding molecules and E3 ligand-recruiting molecules (either VHL- or cereblon-binding ligand).

## Results and Discussion

### Structure-Based Design of METTL3 PROTACs

To design the PROTAC molecules, we started with the structure-based analysis of the binding modes for the two “parent” monovalent METTL3 small-molecule inhibitors, STC-17 and UZH2, that were used as the building components for the following linker attachment to conjugate the E3-ligase-binding ligands. Based on the complex structures of METTL3–METTL14 with STM2457 (PDB 7O2I)^11^ and METTL3-METTL14 with UZH2 (PDB 7O2F),^12^ we identified suitable positions on the STM2457 and UZH2 scaffolds that were pointed towards the solvent front and would permit linkage appendage without interfering with METTL3 binding. Building from STM2457 and its analogues, linkers were attached at the terminal cyclobutene carboxylic acid moiety, and conjugated with either pomalidomide analogues at the 5-amino position, leading to PROTACs **1–5** with varied linker lengths. Alternatively, the VHL-binding ligand was attached to yield the PROTACs **6**–**8** with varied linker lengths. Building from UZH2 and its analogues, linkers were attached at the terminal pyrimidinylaminomethylbenzoic acid moiety, and conjugated with either pomalidomide analogues at the 5-amino position, leading to PROTACs **9–12** with varied linker lengths. Alternatively, the VHL-binding ligand was attached to yield the PROTACs **13** and **14** with varied linker lengths. The former is referred to as the STC-series PROTACs and the latter the MD-series PROTACs in the Results and Discussion section, each with two subclasses depending on the attached E3 ligase-binding ligands.

### Synthesis of the Four Subclasses of METTL3 PROTACs

The four subclasses of METTL3 PROTACs were synthesized according to the general strategy outlined in Figure 3. For the STC-series, the aminoquinazoline scaffold was assembled through palladium-catalyzed allylation, cyclization, and subsequent functional-group manipulations. The terminal cyclobutene moiety of STC-15 was replaced with a cyclobutanecarboxylic acid group, providing a suitable attachment point for linker installation while preserving the key pharmacophoric features required for METTL3 binding. Hydrolysis of the corresponding ester afforded the carboxylic acid intermediate used for PROTAC synthesis. The resulting carboxylic acid warheads were coupled with a panel of (cereblon) CRBN- and (von Hippel-Lindau) VHL-recruiting ligands through alkyl linkers of varying lengths via HATU-mediated amide coupling reaction. For the MD-series, the METTL3-binding scaffold was synthesized through two key intermediates. Intermediate 1 was prepared via a sequence of nitro–Mannich reaction, reductive amination, nucleophilic substitution, Cbz deprotection, and intramolecular lactamization. Intermediate 2 was synthesized from commercially available (4-bromophenyl) methanol through alcohol chlorination, nucleophilic substitution, Buchwald–Hartwig coupling with intermediate 1, and subsequent Boc deprotection. Nucleophilic aromatic substitution of intermediate 2 with the corresponding monosubstituted chloropyrimidines afforded the MD-28 and MD-41 precursors. Subsequent hydrolysis of the ester functionality in the MD-41 precursor furnished the corresponding carboxylic acid warhead for PROTAC synthesis. Final purification by preparative reverse-phase HPLC furnished the desired METTL3 PROTACs **1–14** for the following biological evaluation.

**Figure 3:**
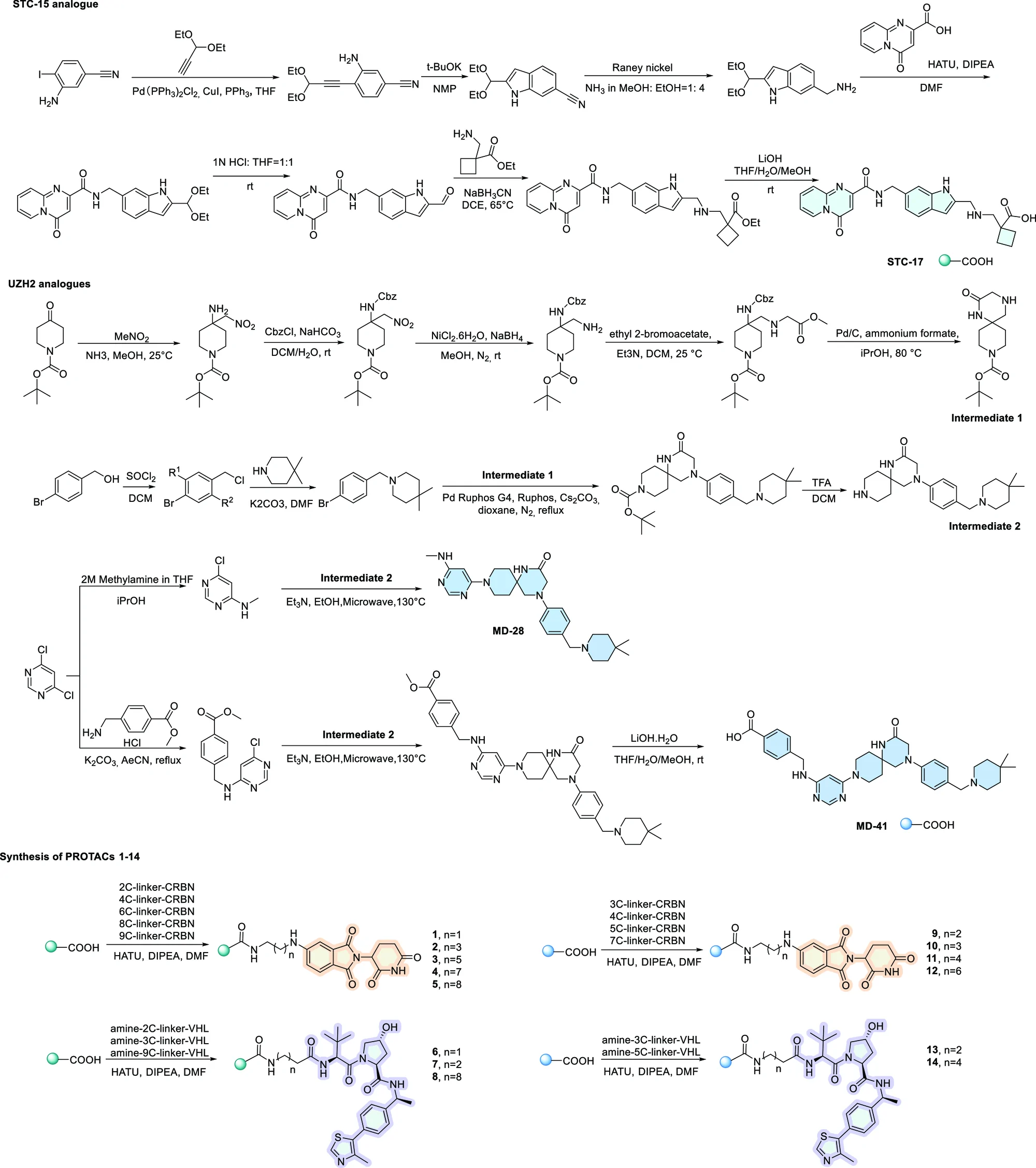
Overview of the synthetic strategies to access METTL3 PROTACs **1–14**. For the STC-series, the aminoquinazoline scaffold was assembled through palladium-catalyzed allylation, cyclization, and subsequent functional-group manipulations to the carboxylic acid STC-17, which was used for PROTACs **1–8** synthesis via coupling with a panel of CRBN- and VHL-recruiting ligands. For the MD-series, the METTL3-binding scaffold was synthesized via spirocyclic intermediates 1 and 2 to afford the pyrimidine precursor MD-41, which features a carboxylic acid for the subsequent PROTACs **9–14** synthesis.

### METTL3-Degrading Activities

Following the design and synthesis of the 14 PROTACs, we initially evaluated their METTL3-degrading activities in cancer cells. Both the MOLM13 and A549 cells were used for Western blot analysis, as METTL3 was widely studied for its high expression in AML cells and its reported association with lung cancers via inhibition of the RIG-I-MAVS pathways.^26^ As shown in Figure 4, at the single treatment concentration of 2 μM, the STC-series PROTACs **4** and **5** showed the most potent METTL3-degrading activities in MOLM13 AML cells (Figure 4A–C), while the MD-series PROTACs **9** and **10** showed the most potent METTL3-degrading activities in A549 lung cancer cells (Figure 4D–F). Combining the structural features of the PROTACs and the observed METTL3-degrading activities, it is revealed that the PROTACs incorporating the CRBN-recruiting ligands showed better activity in inducing METTL3 degradation than the VHL-recruiting ligand-composed PROTACs in both the STC- and MD-series. With the CRBN-containing STC-series, a longer linker composition is preferred, as shown in the cases of **4** and **5**. Within the CRBN-containing MD-series, a comparatively shorter linker composition is preferred, such as **9** and **10**. Despite the observed structure-activity relationship (SAR), it is worth noting that the linker composition variation and linker lengths tested in this small set of PROTACs are limited, so it needs to be further validated with a bigger set of PROTACs to conclude whether this SAR can be generally applied.

**Figure 4:**
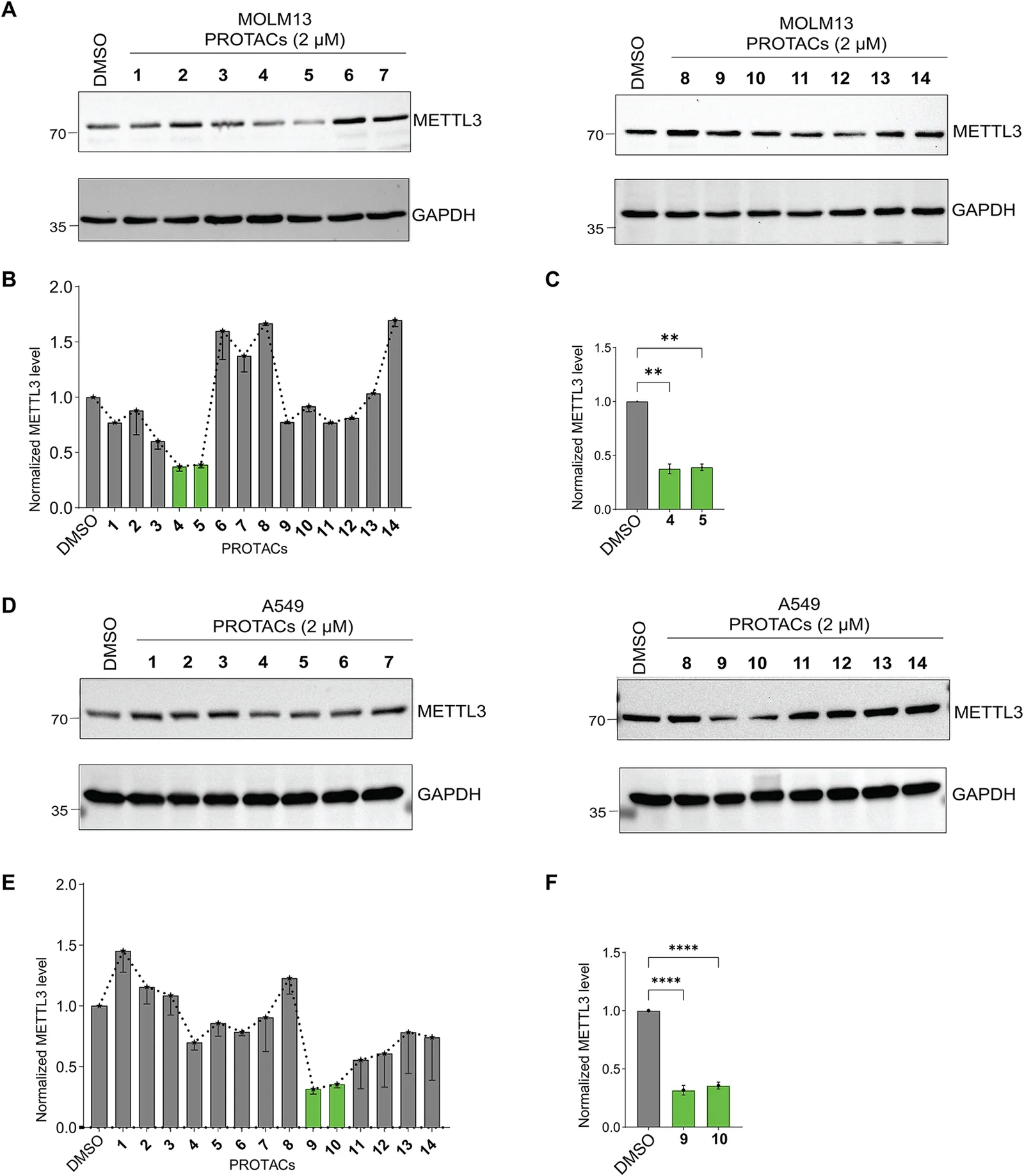
METTL3-degrading activities of the obtained PROTACs. (A) Evaluation of METTL3-degrading activity of PROTACs **1**–**14** (2 μM, for 24 h) in MOLM13 cells. (B) Quantification of the results corresponding to (A). The dashed line connects the protein level of the DMSO control used for normalization (*y* = 1) to the mean protein level for each PROTAC treatment (*n* = 2). The green bars represent the results for the most potent degraders, PROTACs **4** and **5**. (C) Dunnett’s multiple comparison one-way ANOVA test of mean values of METTL3 level for the best-performing PROTACs (**4** and **5**) at 2 μM in the MOLM13 cell line. ** = *p* < 0.01. (D) METTL3-degrading activity of PROTACs **1**–**14** (2 μM, for 24 h) in A549 cells. (E) Quantification results corresponding to (D). The dashed line connects the protein level of the DMSO control used for normalization (*y* = 1) to the mean protein level for each PROTAC treatment (*n* = 3). The green bars represent the results for the most potent degraders, PROTACs **9** and **10**. (F) Dunnett’s multiple comparison one-way ANOVA test of mean values of METTL3 level for the best-performing PROTACs (**9** and **10**) at 2 μM in the A549 lung cancer cells, relative to DMSO control. **** = *p* < 0.0001.

### METTL3 PROTACs Induced Varied Cellular Morphological Changes in Cancer Cells

CPA is a powerful phenotypic profiling method for bioactive molecules, especially new chemical modalities including PROTACs and RIBOTACs, as it permits the cellular-level characterization of mode of action, safety, and toxicity profile, together with various physicochemical and biological properties, in an unbiased manner, which is not feasibly achievable by evaluating individual components of PROTACs. Therefore, we proceeded with the CPA analysis for the 14 METTL3 PROTACs with the aim of uncovering “hidden” biological information that would otherwise not be obtainable from other target-based evaluations. Previously, CPA has been shown to be a useful method to unfold new biological information for potential targets and mechanism of action.^27–33^ Recently, we demonstrated that CPA can be useful to indicate the new biological performance of small molecules and bifunctional molecules targeting RNA-binding proteins,^22,34^ and RIBOTACs.^23^

The CPA assays were performed in the human osteosarcoma U2OS cells, whose flat and well-spread morphology facilitates high-content imaging and segmentation of subcellular features. The CPA in U2OS cells was conducted at three different concentrations (10, 30, and 50 μM). The microscopic cell images obtained after staining eight cellular compartments (including nucleus, nucleoli, mitochondria, endoplasmic reticulum, Golgi, plasma membrane, actin cytoskeleton, and cytosolic RNA) with six different dyes were used to extract a total of 579 morphological features employing high-content screening and image analysis. This resulted in the morphological fingerprints for each compound at each specific concentration that collectively were used to compare the biological similarities and differences among the tested compounds. As a quantitative measurement of bioactivity, a quantified induction value based on the generated fingerprints described the fraction of significantly altered parameters in comparison to the vehicle control (%, in percent). A compound is considered biologically active with an induction value of >5%,^35^ the results of which are summarized in Figure 5. The generated heatmap including the fingerprints for the active compounds is shown in Figure 6.

**Figure 5:**
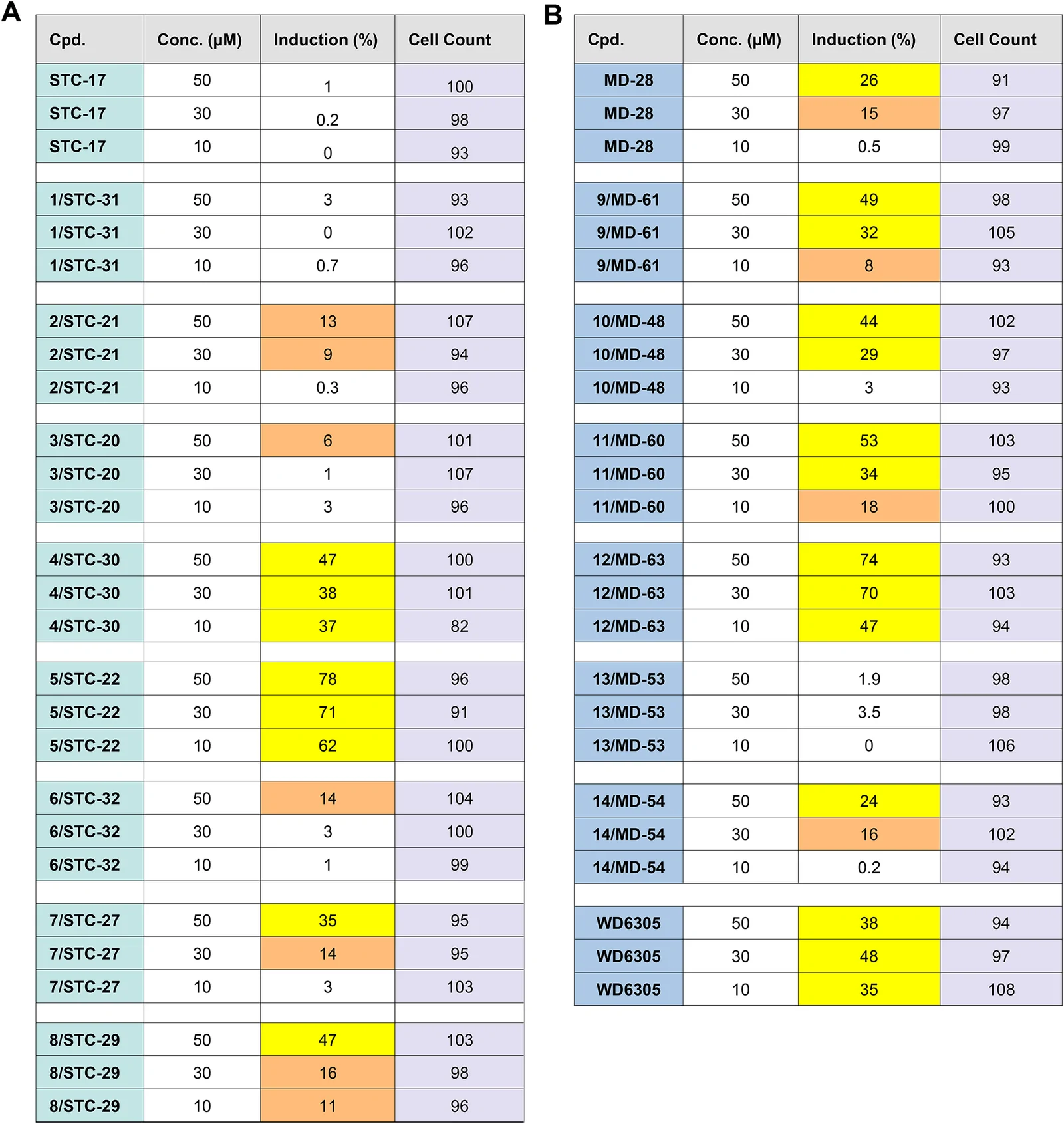
Quantified results of the cell painting assay measuring cellular morphological changes induced by the METTL3 PROTACs. (A) For the STC series PROTACs **1**–**8**, the quantified values are shown as Induction (%) together with the parent monovalent METTL3 inhibitor STC-17. (B) For the MD-series PROTACs **9**–**14**, as well as the reported METTL3 PROTAC WD6305, the quantified values are shown as Induction (%) together with the parent monovalent METTL3 inhibitor MD-28. For both panels: Each compound was tested at three different concentrations (conc.: 50, 30, and 10 μM). For the Induction: values of more than 20% are highlighted in bright yellow; values of less than 20% but more than 5% are highlighted in ocher; values of less than 5% are deemed biologically inactive in the CPA analysis. For the relative cell count in the U2OS cells, values of less than 50% indicate being too cytotoxic in the CPA assay, which does not apply to all the compounds tested in this study.

**Figure 6:**
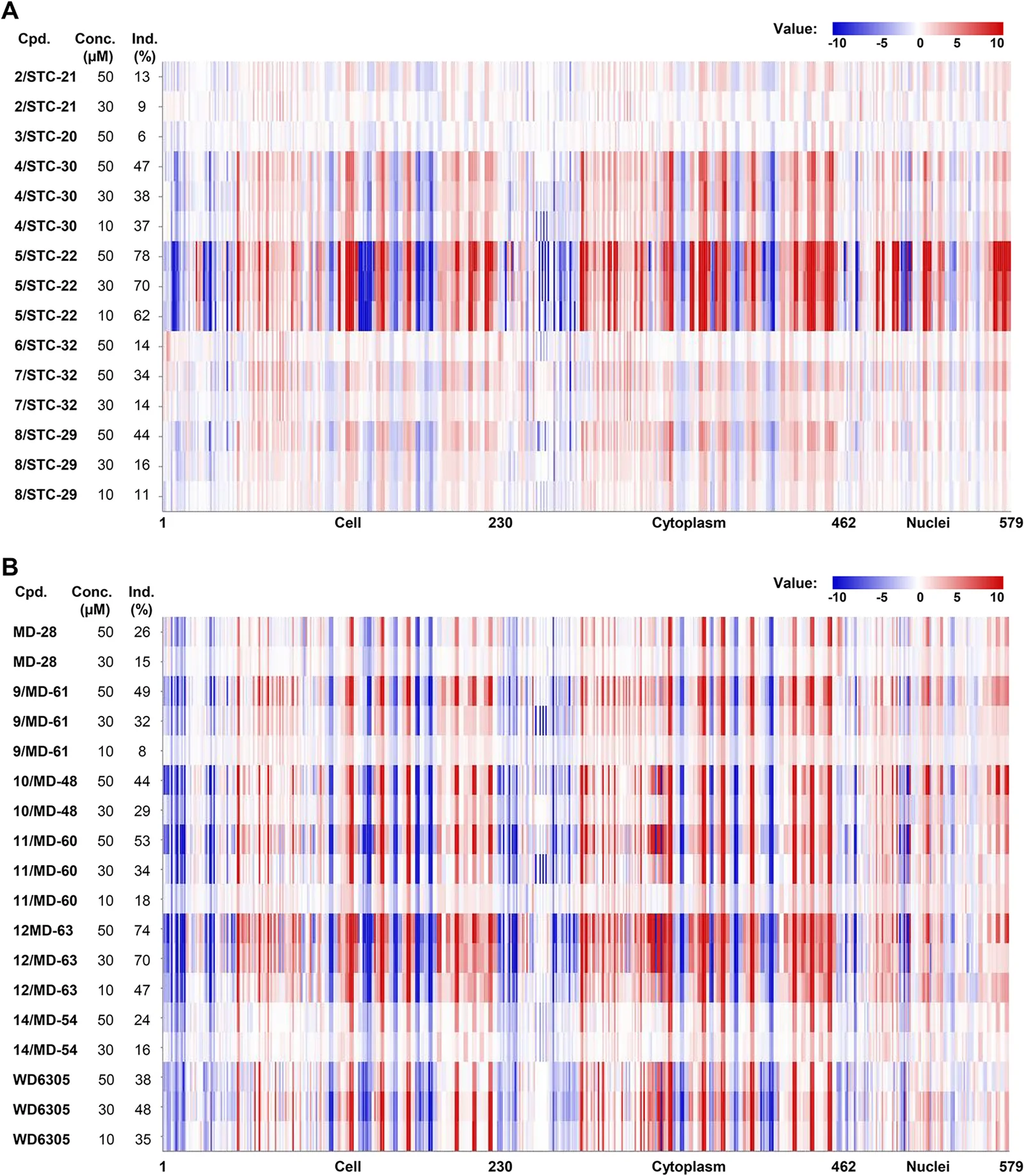
Heatmap of the METTL3-targeting PROTACs in the cell painting assay. Each of the 14 METTL3 PROTACs was tested at three different concentrations (conc.: 50, 30, and 10 μM). Here is the summary of all active testing results (>5% induction) at the varied concentrations. (A) CPA heatmap of active PROTACs in the STC series using STC-17 as the METTL3-binding component. As STC-17 was not active in CPA at the three tested concentrations, PROTAC **2**/STC-21 was set as the reference fingerprint (100% biological similarity). The following PROTACs **3**–**8** are compared to STC-21 for their biological similarity (Biol. Sim. %). (B) CPA heatmap of active PROTACs in the MD series using MD-28 as the METTL3-binding component, as well as the reported METTL3 PROTAC WD6305. The top line of the fingerprint is the result for MD-28, which is set as the reference fingerprint. The following PROTACs **9**–**14** and WD6305 are compared to MD-28 for their biological similarity (Biol. Sim. %). For both panels (A, B): The set of 579 parameters indicated in the *x*-axis is divided into parameters related to the cell (1–229), cytoplasm (230–461), and nuclei (462–579). Increased parameters are shown in red, and decreased parameters are shown in blue.

As shown in Figures 5 and 6, the METTL3 PROTACs showed varied activities in the CPA. The corresponding induction value was calculated to describe the fraction of significantly altered parameters (in percent) in comparison to the vehicle control. Overall, except for PROTAC **1**, most STC-17-derived PROTACs showed better activity in CPA in comparison with that of the monovalent inhibitor STC-17 alone. Among the STC-series PROTACs **1**–**8** that used an STC-17 analogue as the METTL3-binding component, the pomalidomide analogue with 5-amino-linked PROTAC **4** and **5** showed the most potent activities in CPA. For example, PROTAC **5** showed an induction value of >60% at 10 μM. In comparison, PROTACs **1**–**3** that feature shortened linkers were inactive at tested concentrations, except for the weak activity shown by PROTAC **2** at 50 μM (induction >10%). For PROTACs **6**–**8** that were built from the VHL-binding ligand, the tendency for a longer linker to be associated with better CPA activity is echoed, as PROTAC **8** showed the best activity among the three. Among the MD-series PROTACs **9**–**14** that used the UZH2 analogue MD-28 as the METTL3-binding component, the pomalidomide analogue with 5-amino-linked PROTAC **12** showed the most potent activity in CPA, with an induction value of ∼50% at 10 μM. In contrast to the STC-series PROTACs **1**–**8**, the MD-series PROTACs showed both increased and decreased activity in comparison with that of the parent monovalent METTL3 inhibitor MD-28. That being said, the same trend of PROTACs with a longer linker showing better activity in CPA is replicated in the MD-series PROTACs. In addition to the STC-series PROTACs **1**–**8**, the reported METTL3 PROTAC WD6305 built from UZH2 was tested to provide comparison data. WD6305 showed consistent activity in the CPA under the three tested concentrations. Despite the varied CPA activities, all PROTACs showed no obvious cytotoxicity under the tested concentrations (here, “too toxic” is defined by the relative cell count number <50%).

### Cross-Biosimilarities among the Active METTL3 PROTACs

For the active PROTACs inducing cellular morphological changes in CPA, we proceeded with cross-biosimilarity analysis, as shown in Figure 7. Among the STC series PROTACs **1**–**8**, high cross-biosimilarities (>85%) were observed for individual PROTACs under different concentrations or mainly among the most active PROTACs **4**, **5**, and **8**. Among the MD-series PROTACs **9**–**14**, high cross-biosimilarities were observed widely among the PROTACs. Apart from the different cross-biosimilarities within the two individual series, there are also high cross-biosimilarities observed between PROTAC **4** and PROTACs **9**, **10**, and **12**, either at 10 or 30 μM. Collectively, the cross-biosimilarity analysis of the active RIBOTACs demonstrated that, first, by using different monovalent METTL3 small-molecule inhibitors, PROTACs of different biological performance could be obtained by applying under different concentrations; second, even for PROTACs constructed from the same monovalent METTL3 inhibitor and E3 ligase-binding ligand, varied biological activities can be obtained by varying linker compositions and lengths.

**Figure 7:**
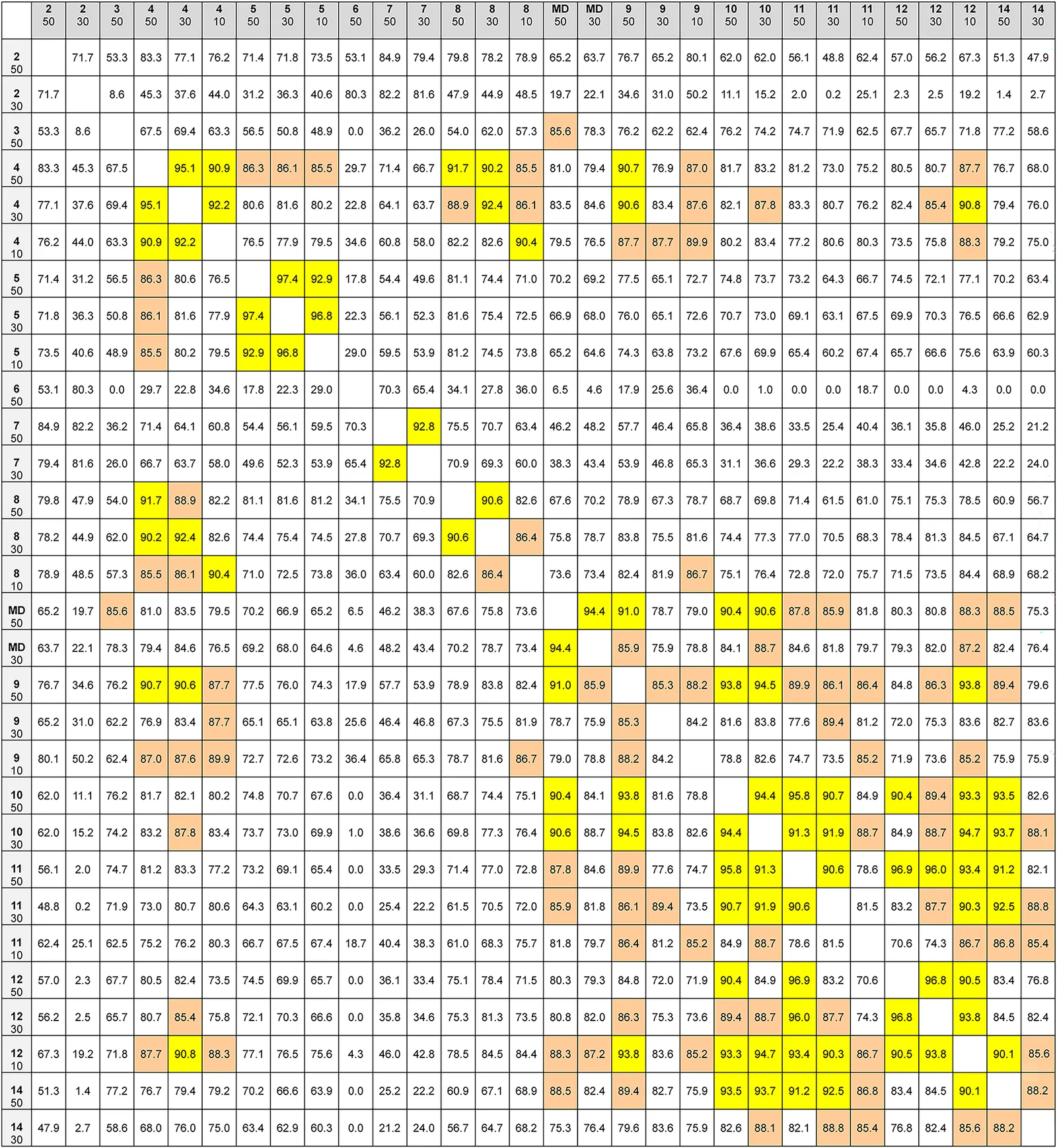
Cross-biosimilarities among the active METTL3-targeting PROTACs (>5% induction) tested in the CPA analysis. The bolded numeric numbers in the first row and first column on the left indicate the PROTAC molecules; the nonbolded numeric numbers in the first row and first column on the left indicate the concentration of the corresponding PROTAC molecules (μM). The percent values with one digit after the decimal point are given to indicate cross-biosimilarities (%). Biosimilarities of more than 90% are highlighted in bright yellow; biosimilarities of more than 85% are highlighted in ocher.

### METTL3 PROTACs and Biosimilarities with Established Bioclusters

Bioactivity clusters were established in the CPA analysis by profiling reference compounds of annotated bioactivities with identified biological targets and characterized modes of action.^27,29^ Comparing the CPA subprofiles with established bioactivity clusters is, therefore, a powerful approach to study the targets and mechanism of action of unknown compounds or new chemical modalities.^29^ As shown in Figure 8, the subprofile analysis revealed the consistent observation of an overall high level of shared biosimilarity among the PROTACs. For the STC-series PROTACs **1**–**8**, although the majority showed similarities with the lysosomotropism and cholesterol homeostasis regulation (LCH) cluster (up to 77%), only PROTAC **4** showed high similarity (>85%) at 30 μM. Comparing the subprofile analysis results between STC-17 and PROTACs **1**–**8** clearly showed that the formation of the bivalent bifunctional PROTACs led to significantly different biological activities in comparison with the parent monovalent METTL3 inhibitor STC-17. For the MD-series PROTACs **9**–**14**, most showed the highest similarity with the LCH cluster (>90%), except for PROTAC **13** (∼50% with the LCH cluster and ∼70% with the mitochondrial stress regulation cluster). Additionally, the subprofile analysis of WD6305 also showed high similarity with the LCH cluster (up to 89%), demonstrating that the high LCH cluster similarity could be a universal phenotype triggered by METTL3 degradation. Alternatively, the overall high biosimilarity with the LCH cluster indicates that the obtained METTL3 PROTACs could be taken up into lysosomes in cells and function as lysosomotropic agents,^36^ the detailed mechanism of which warrants further exploration.

**Figure 8:**
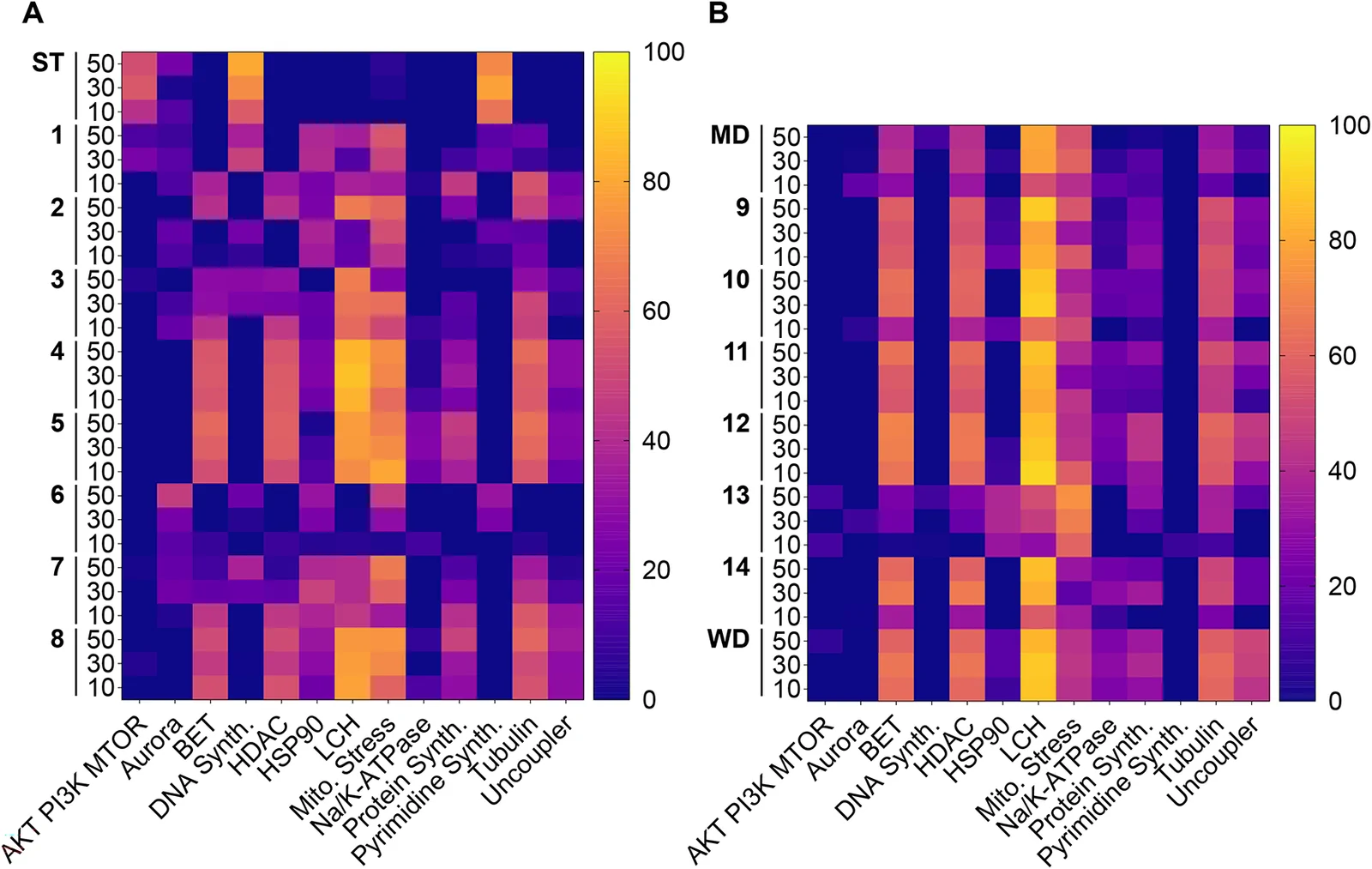
Subprofile analysis of the METTL3 PROTACs for their cluster biosimilarity at different concentrations. (A) Cluster biosimilarity analysis of the STC series METTL3 PROTACs **1**–**8**. (B) Cluster biosimilarity analysis of the MD-series METTL3 PROTACs **9**–**14** and the reported METTL3 PROTAC WD6305 (WD). For both panels, the biosimilarity values are visualized by a gradient series of colors and quantified as a percentage (deep purple-blue, 0%; bright yellow, 100%). ST: STC-17; MD: MD-28. The established bioactivity clusters are those associated with AKT/PI3K/MTOR, aurora kinase, bromodomain and extra-terminal domain (BET), DNA synthesis histone deacetylase (HDAC), heat shock protein 90 (HSP90), lysosomotropism and cholesterol homeostasis (LCH) regulation, mitochondrial stress regulation, Na^+^/K^+^ ATPases, protein synthesis de novo pyrimidine biosynthesis, tubulin, and uncoupling of the mitochondrial proton gradient (Uncoupler).

### METTL3 Degradation in U2OS Cells

Given that the CPA was performed in U2OS cells, we compared the degrading potency of the most active PROTACs **4**, **5**, **9**, and **10** with that of the reported METTL3 degrader WD6305 in U2OS cells (Figure 9). At 2 μM, WD6305 showed the most potent degradation activity (>90%). In comparison, the four PROTACs showed less METTL3-degrading activity, ranging between ∼50% and ∼10% degradation (Figure 9A). The concentration-dependent degradation was observed for PROTAC **9**, with a maximum degradation of 50% observed at 10 μM in U2OS cells (Figure 9B). Furthermore, it was demonstrated that the proteasome inhibitor MG132 partially rescued the induced METTL3 degradation by WD6305 and PROTACs **4** and **9** (Figure 9C). The partial-rescue result indicates that the observed degradation may proceed beyond the canonical ubiquitin-proteasome machinery utilized by PROTACs. Other plausible explanations for the partial-rescue result include non-proteasomal turnover of METTL3, indirect off-target suppression of METTL3 expression, as well as experimental factors related to MG132-associated cytotoxicity. The PROTACs showed no inhibitory activity on cellular viability in U2OS cells in CPA. We then tested the cellular viability of the selected PROTACs **4**, **5**, **9**, and **10** in MOLM13 and A549 cells at different concentrations (Figure 10). At concentrations up to 10 μM (0.1, 1.0, and 10 μM), all PROTACs showed weak or moderate inhibitory activity against MOLM13 and A549 (< 50%). At 50 μM, PROTAC **9** showed the most potent inhibitory activity against MOLM13 (∼50%) and A549 (∼100% inhibition). Therefore, the observed cellular inhibitory activity does not fully correlate with the CPA activities of the PROTACs. Additionally, a remaining unexplored question in this study is the exact contribution of METTL3 degradation to the observed cellular activities.

**Figure 9:**
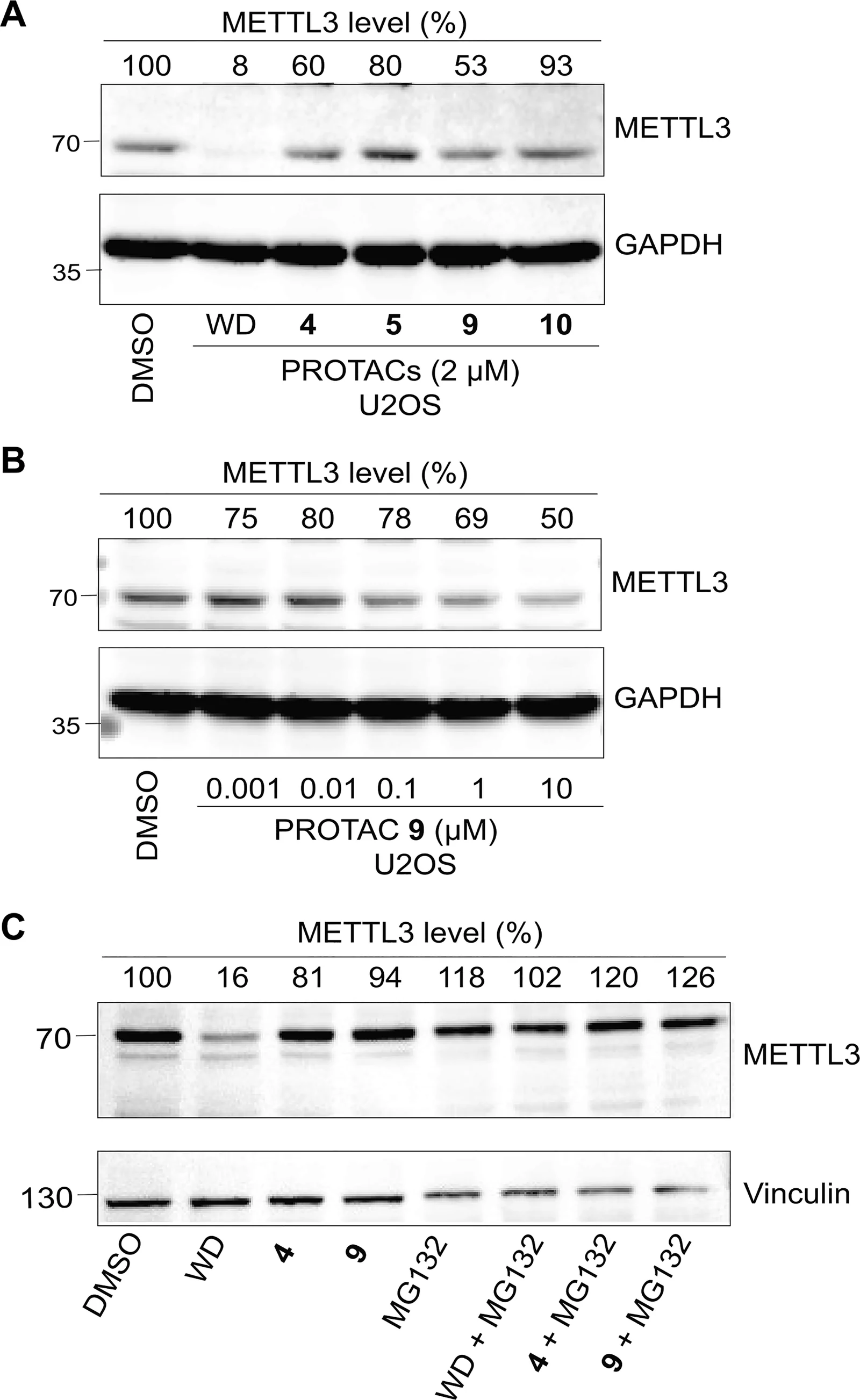
Evaluation of selected METTL3-degrading PROTACs in U2OS cells. (A) Degradation of METTL3 tested by Western blot for selected METTL3 PROTACs (**4**, **5**, **9**, and **10**) and the reported degrader WD6305 (WD) in U2OS cells at 2 μM. The data represent the relative percentage mean of METTL3 levels compared to the DMSO negative control for two independent biological replicates; GAPDH serves as the loading control. (B) Concentration-dependent degradation of METTL3 by PROTAC **9** in U2OS cells. The data represent the relative percentage mean of METTL3 levels compared with the DMSO negative control in two independent biological replicates; GAPDH serves as a loading control. (C) Evaluation of the proteasome-mediated degradation mechanism by using MG132 (10 μM) as the proteasome inhibitor to rescue METTL3 degradation induced by WD6305 or PROTACs **4** and **9** (2 μM) in U2OS cells. Results are shown from two independent biological replicates. Vinculin serves as the loading control.

**Figure 10:**
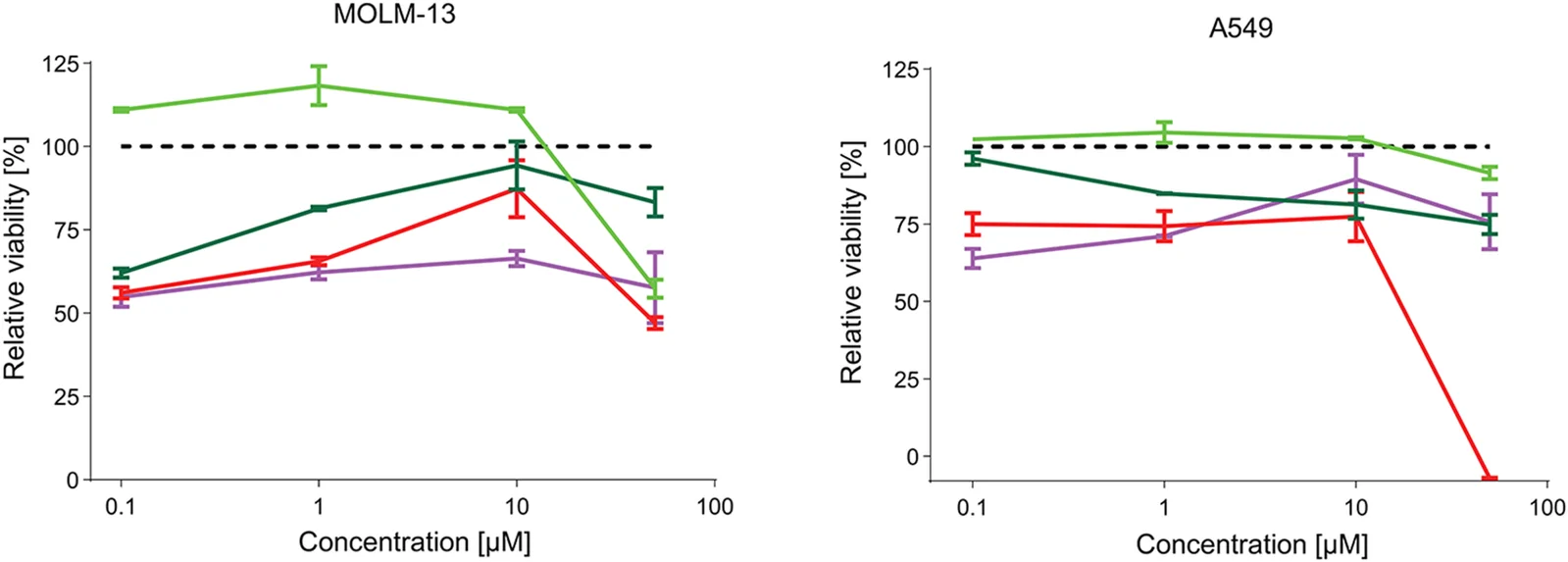
Growth inhibition activity in MOLM13 (left) and A549 (right) cells for selected PROTACs. Cells were treated with different concentrations (0.1, 1.0, 10, and 50 μM) of the PROTACs for 72 h, and viability was measured in the CellTiter assay. The dashed line shows the DMSO control used for relative cell viability normalization (*y* = 100%), and the error bars represent the standard error of the mean of two independent biological replicates. The colored lines indicate results for PROTACs **4** (light green), **5** (dark green), **9** (red), and **10** (purple).

## Conclusion

In this study, we successfully designed and synthesized 14 new METTL3-targeting PROTAC degraders across four subclasses based on two monovalent METTL3-binding scaffolds (STC-17 analogue and the UZH2 analogue). We also report here the first application of unbiased phenotypic profiling in the characterization of METTL3-degrading PROTACs via the CPA. The results revealed that varied linker lengths and the choice of E3 ligase recruiting ligand critically influence both the degradation activity and cellular morphological profiles. Specifically, PROTACs **4** and **5** (STC series with pomalidomide) exhibited potent METTL3 degradation in MOLM13 AML cells, while PROTACs **9** and **10** (MD series with pomalidomide) showed potent activity in A549 lung cancer cells.

The application of CPA for the characterization of METTL3 PROTACs uncovered that these bifunctional degraders induce concentration-dependent cellular morphological changes distinct from those of their parent monovalent inhibitors, with longer linkers generally correlating with higher induction values. Strikingly, cross-biosimilarity analysis revealed that the active PROTACs showed high similarity (>85% for STC-series, >90% for MD-series) with the LCH regulation cluster, suggesting that these PROTACs may function in part as lysosomotropic agents. However, this assumption must be further validated.

Looking forward, the insights gained from this study open several avenues for future research. First, the observed correlation between linker length and CPA activity underscores the need for systematic optimization of linker composition, rigidity, and attachment sites to enhance degradation efficacy while modulating lysosomotropic properties. Second, the high biosimilarity with the LCH cluster may stem from the lysosomotropic accumulation behavior of these PROTACs rather than being the direct downstream effect of METTL3 depletion. The clarification of this observed phenotypic fingerprint warrants further mechanistic investigation and would be useful to advance lead PROTACs development into in vivo studies. Finally, the CPA workflow is a valuable phenotypic evaluation approach that can be readily extended to future generations of METTL3 degraders and, more broadly, to PROTACs targeting other RNA-modifying proteins. Collectively, this work diversifies the METTL3-targeting chemical repertoire and demonstrates CPA as a powerful morphological profiling approach to complement existing methods for PROTAC characterization.

## Experimental Section

### Synthetic Procedure

The general chemical information, synthetic procedures, characterization of the R-Score molecules, as well as NMR spectra, are provided in the Supporting Information.

### Cell Culture

MOLM13, A549, and U2OS cells were purchased from the Leibniz German Collection of Microorganisms and Cell Culture GmbH (DSMZ, Braunschweig, Germany). MOLM13 cells were cultured in sterile-filtered RPMI-1640 medium (Fisher Scientific GmbH, 15303541) supplemented with 10% (v/v) fetal bovine serum (Sigma-Aldrich, S0615-500) and 1% penicillin-streptomycin (PAN-Biotech, 15140122). A549 and U2OS cells were cultured in sterile-filtered DMEM medium (PAN-Biotech, P04-03600) supplemented with 1% (v/v) GlutaMAX (Fisher Scientific GmbH, 35050038), 10% (v/v) fetal bovine serum (Sigma-Aldrich, S0615-500), and 1% penicillin-streptomycin (PAN-Biotech, 15140122). Cells were maintained at 37 °C in a humidified incubator with 5% CO_2_. Cells were routinely tested for mycoplasma contamination using Mycoplasma Detection Kit (Jena BioScience) and were confirmed to be negative.

### Western Blot

A549 and U2OS cells were seeded in 6-well plates at 0.35 × 10^6^ cells per well, and MOLM13 cells were seeded at 1.0 × 10^6^ cells per well. PROTAC stock solutions were prepared in DMSO (10 mM). Cells were treated with METTL3 PROTACs **1**–**14**, MG132, WD6305 at the indicated final concentrations or with DMSO vehicle control for 24 h at 37 °C and 5% CO_2_. The final DMSO concentration was 0.1% (v/v) in all samples. After PROTAC treatment, A549 and U2OS cells were washed twice with ice-cold PBS and lysed in RIPA buffer containing 50 mM Tris–HCl pH 8.0, 150 mM NaCl, 0.5% sodium deoxycholate, 0.1% SDS, 1% NP-40, 2 mM MgCl_2_ and 0.5 mM DTT. RIPA buffer was supplemented freshly with cOmplete EDTA-free protease inhibitor, PhosSTOP phosphatase inhibitor, and benzonase nuclease. MOLM13 cells were collected by centrifugation at 400*g* for 5 min at 4 °C, washed twice with ice-cold PBS, and lysed in 2% SDS supplemented with 5 mM β-mercaptoethanol. MOLM13 lysates were incubated at 95 °C for 30 min. The total protein concentration was determined using the detergent-compatible Bradford assay kit according to the manufacturer’s instructions.

Equal amounts of total protein were mixed with SDS-PAGE sample buffer and separated by SDS-PAGE using 10–12% polyacrylamide gels. Proteins were transferred to 0.2 μm nitrocellulose membranes using a wet-transfer system. Membranes were blocked for 45 min at room temperature in 5% non-fat milk in PBS-T. PBS-T consisted of PBS supplemented with 0.05% Tween-20. Membranes were incubated overnight at 4 °C with primary antibodies diluted in 5% BSA in PBS-T. The following primary antibodies were used: anti-METTL3, anti-GAPDH, and anti-vinculin, at the dilutions listed in the Materials table. After primary antibody incubation, membranes were washed three times for 10 min each in PBS-T and incubated for 1 h at room temperature with the appropriate HRP-conjugated secondary antibody diluted in 5% non-fat milk in PBS-T. Membranes were then washed three times in PBS-T and twice in PBS for 10 min each. Chemiluminescent signal was developed using SuperSignal West Pico PLUS substrate and imaged using a ChemoStar Plus chemiluminescence imager. Western blot band intensities were quantified using Bio-Rad Image Lab software (version 6.0.1) and analyzed in GraphPad Prism (version 11.0.2). METTL3 signal was normalized to GAPDH or vinculin loading controls, as indicated, and then normalized to the DMSO-treated control. Representative Western blot figures were assembled using Adobe Illustrator 2025. Experiments were performed in two to three independent biological replicates.

### Cell Viability Assay

The A549 and MOLM13 cells were seeded in 96-well plates at a density of 5 × 10^3^ cells/well and 6 × 10^3^ cells/well, respectively. For A549, cells were seeded in 100 μL of growth medium and incubated for 24 h, then the growth medium was replaced with 100 μL of fresh growth medium containing the respective PROTACs in triplicate at final concentrations of 0.1, 1, 10, and 50 μM or DMSO control. For MOLM13, cells were seeded in 50 μL of growth medium and supplemented with an additional 50 μL of growth medium containing the respective PROTACs in triplicate at final concentrations of 0.1, 1, 10, and 50 μM or DMSO control. After treatments, cells were incubated for 72 h at 37 °C in a 5% CO_2_ incubator. Cell viability was determined using a CellTiter 96_AQueous_ cell viability assay (Promega: G3582) based on the detection of colored formazan product produced by dehydrogenase enzymes in metabolically active cells, following the manufacturer’s instructions. Briefly, 20 μL of CellTiter 96_AQueous_ One Solution was added to each well containing the samples in 100 μL of culture medium. The samples were incubated at 37 °C for 2 h in a 5% CO_2_ incubator. The absorbance was measured at 490 nm using BMG LABTECH Omega 96-well plate reader. The background absorbance value was obtained from wells containing CellTiter 96_AQueous_ reagent and growth medium without the cells. Outliers were removed, and background absorbance was subtracted. The graph of DMSO-normalized biological duplicates was plotted using GraphPad Prism (version 11.0.2).

### Cell Painting Assay

The cell painting assay was performed following the previously described methods.^30,31^ Initially, 5 μL of U2OS medium was added to each well of a 384-well plate (Revvity PhenoPlate 384). Subsequently, U2OS cells were seeded at a density of 1600 cells per well in 20 μL medium. The plate was incubated for 10 min at ambient temperature, followed by an additional 4 h incubation (37 °C, 5% CO2). Compound treatment was performed with the Echo 520 acoustic dispenser (Beckman-Coulter) at final concentrations of 10, 30, or 50 μM. Incubation with the compound was performed for 20 h (37 °C, 5% CO2). Subsequently, mitochondria were stained with Mito Tracker Deep Red (Thermo Fisher Scientific, Cat. No. M22426). The Mito Tracker Deep Red stock solution (1 mM) was diluted to a final concentration of 100 nM in prewarmed medium. The medium was removed from the plate, leaving 10 μL residual volume, and 25 μL of the Mito Tracker solution was added to each well. The plate was incubated for 30 min in darkness (37°C, 5% CO_2_). To fix the cells, 7 μL of 18.5 % formaldehyde in PBS was added, resulting in a final formaldehyde concentration of 3.7 %. Subsequently, the plate was incubated for another 20 min in darkness (RT) and washed three times with 70 μL of PBS. (Agilent Washer Elx405). Cells were permeabilized by addition of 25 μL of 0.1% Triton X-100 to each well, followed by 15 min incubation (RT) in darkness. The cells were washed three times with PBS, leaving a final volume of 10 μL. To each well 25 μL of a staining solution were added, which contains 1% BSA, 5 μL/ml Phalloidin (Alexa594 conjugate, Thermo Fisher Scientific, A12381), 25 μg/mL Concanavalin A (Alexa488 conjugate, Thermo Fisher Scientific, Cat. No. C11252), 5 μg/mL Hoechst 33342 (Sigma, Cat. No. B2261-25 mg), 1.5 μg/mL WGA-Alexa594 conjugate (Thermo Fisher Scientific, Cat. No. W11262) and 1.5 μM SYTO 14 solution (Thermo Fisher Scientific, Cat. No. S7576). The plate is incubated for 30 min (RT) in darkness and washed three times with 70 μL PBS. After the final washing step, the PBS was not aspirated. The plates were sealed and centrifuged for 1 min at 50*g*. The plates were prepared in triplicate with shifted layouts to reduce plate effects and imaged using a Micro XL High-Content Screening System (Molecular Devices) in 5 channels (DAPI: Ex350-400/Em410-480; FITC: Ex470-500/Em510-540; Spectrum Gold: Ex520-545/Em560-585; TxRed: Ex535-585/Em600-650; Cy5: Ex605-650/Em670-715) with 9 sites per well and 20× magnification (binning 2). The generated images were processed with the *CellProfiler* package (https://cellprofiler.org/, version 3.0.0) on a computing cluster of the Max Planck Society to extract 1716 cell features per microscope site. The data were then further aggregated as medians per well (9 sites -> 1 well), then over the three replicates. Further analysis was performed with custom *Python* (https://www.python.org/) scripts using the *Pandas* (https://pandas.pydata.org/) and *Dask* (https://dask.org/) data processing libraries as well as the *Scientific Python* (https://scipy.org/) package (separate publication to follow).

From the total set of 1716 features, a subset of highly reproducible and robust features was determined using the procedure described by Woehrmann et al.^37^ Two biological repeats of one plate containing reference compounds were analysed. For every feature, its full profile over each whole plate was calculated. If the profiles from the two repeats showed a similarity no less than 0.8 (see below), the feature was added to the set. This procedure was only performed once and resulted in a set of 579 robust features out of the total of 1716 that were used for all further analyses. The phenotypic profiles were compiled from the Z-scores of all individual cellular features, where the Z-score is a measure of how far away a data point is from a median value. Specifically, Z-scores of test compounds were calculated relative to the Median of DMSO controls. The phenotypic compound profile is then determined as the list of Z-scores of all features for one compound. In addition to the phenotypic profile, an induction value was determined for each compound as the fraction of significantly changed features, in percent. Similarities of phenotypic profiles (termed *Biosimilarity*) were calculated from the correlation distances (CD) between two profiles (https://docs.scipy org/doc/scipy/reference/generated/scipy.spatial.distance.correlation.html). The Biosimilarity is then defined as *Biosimilarity* = 1 – CD. Biosimilarity values smaller than 0 are set to 0, and the Biosimilarity is expressed in percent (0–100).

## Supplementary Material



## Notes

General chemistry information, synthetic procedures, compound characterizations, and ^1^H NMR and ^13^C NMR spectra (PDF)
