# Supplementary material for: Cellular Morphological Profiling of PROTAC Degraders of the m6A RNA Methyltransferase METTL3
Source: ACS Bio Med Chem Au. 2026 Oct 6;6(5):708–22. doi: 10.1021/acsbiomedchemau.6c00136 (PMC13692740; doi:10.1021/acsbiomedchemau.6c00136)
Supplement: Supplementary file 1 [file bg6c00136_si_001.pdf]

Supporting Information

Cellular Morphological Profiling of PROTAC Degraders of the m6A RNA Methyltransferase METTL3

Mao Jiang,[1-4,#] Chukwudi T. Ugwu,[4,#] Laura Wortmann,[1-4] Jeffrey L. Schloßhauer,[1-4] Christian Begemann,[1-4] Sonja Sievers,[2,4,5] Katarzyna W. Kliza,[4,*] and Peng Wu[1-4,*]

[1]Chemical Genomics Centre, Max Planck Institute of Molecular Physiology, Dortmund 44227, Germany

[2]Department of Chemical Biology, Max Planck Institute of Molecular Physiology, Dortmund 44227, Germany

[3]Faculty of Chemistry and Chemical Biology, TU Dortmund University, Dortmund 44227, Germany

[4]Max Planck Institute of Molecular Physiology, Dortmund 44227, Germany

[5]Compound Management and Screening Center, Dortmund 44227, Germany

[#]Equally contributed authors

Corresponding Authors:

P. Wu, email: peng.wu@mpi-dortmund.mpg.de; peng.wu@tu-dortmund.de

K. Kliza, email: Katarzyna.kliza@mpi-dortmund.mpg.de

Table of Contents

General Chemical Information………………………………………….... S03

Synthetic Procedures and Compound Characterization ………….….… S04

NMR Spectra………………………………………………………………. S29

References………………………………………………………………… S40

General Chemical Information

The commercially available reagents were used without any further purification, unless otherwise mentioned. Solvents used for silica gel column chromatography were of laboratory grade. Dry solvents were purchased from Acros, Fischer Scientific and/or VWR, and used without further treatment. Oxygen and/or moisture sensitive solutions were transferred under inert gas atmosphere using cannulas and syringes. Analytical thin-layer chromatography (TLC) was performed on silica coated aluminum plates (Merck 60 F254) and visualization of products proceeded with UV irradiation (254 nm and/or 356 nm) or through potassium permanganate stain (1.5 g $KMnO_4$, 10 g K_2CO_3 in 1.25 mL of 10% aq. NaOH and 200 mL water). Analytical UHPLC-MS was performed on an Agilent 1260 II Infinity system equipped with a mass detector (UHPLC column: Zorbax Eclipse C18 Rapid Resolution 2.1x50 mm 1.8µm; LC-MS column: InfinityLab Poroshell 120 EC-C18, 2.1x150, 2.7 µm). Appropriate gradient systems were applied by water (+ 0.1% TFA) and mixing Acetonitrile (+ 0.1% TFA). The purification of crude products was performed by silica gel column chromatography (Merck 60, particle size 0.040-0.063 mm) using indicated solvents. ^{1}H NMR and ^{13}C NMR spectra were recorded with either a Bruker AV 400 Avance III HD (NanoBay), Agilent Technologies DD2, Bruker AV 500 Avance III HD (Prodigy), Bruker Avance NEO – 500 MHz, Bruker AV 600 Avance III HD (CryoProbe) or a Bruker AV 700 Avance III HD (CryoProbe) spectrometers. Data are reported in parts per million (ppm) with reference to the used deuterated solvent ($CDCl_3$: 7.26 ppm, 77.16 ppm; DMSO-d_6: 2.50 ppm, 39.52 ppm). Chemical shift values are reported in ppm, multiplicity (s = singlet, d = doublet, t = triplet, dd = double doublet, m = multiplet, br = broad signal, q = quintet, dt = double of triplets, td = triplet of doubles), integration values, and coupling constant values in Hz. Signals were assigned to their corresponding hydrogens or carbons based on 2D NMR correlations ($^{1}H/^{1}H$ COSY, $^{1}H/^{1}H$ NOESY, $^{1}H/^{13}C$ HSQC, $^{1}H/^{13}C$ HMBC). High-resolution mass spectrometry (HRMS) was performed on an LTQ Orbitrap mass spectrometer coupled to an Accela HPLC-System (HPLC column: Hypersyl GOLD, 50 mm x 1 mm, particle size 1.9 µm, ionization method: electrospray ionization, ESI).

Synthesis of STC-15 analogue STC-17

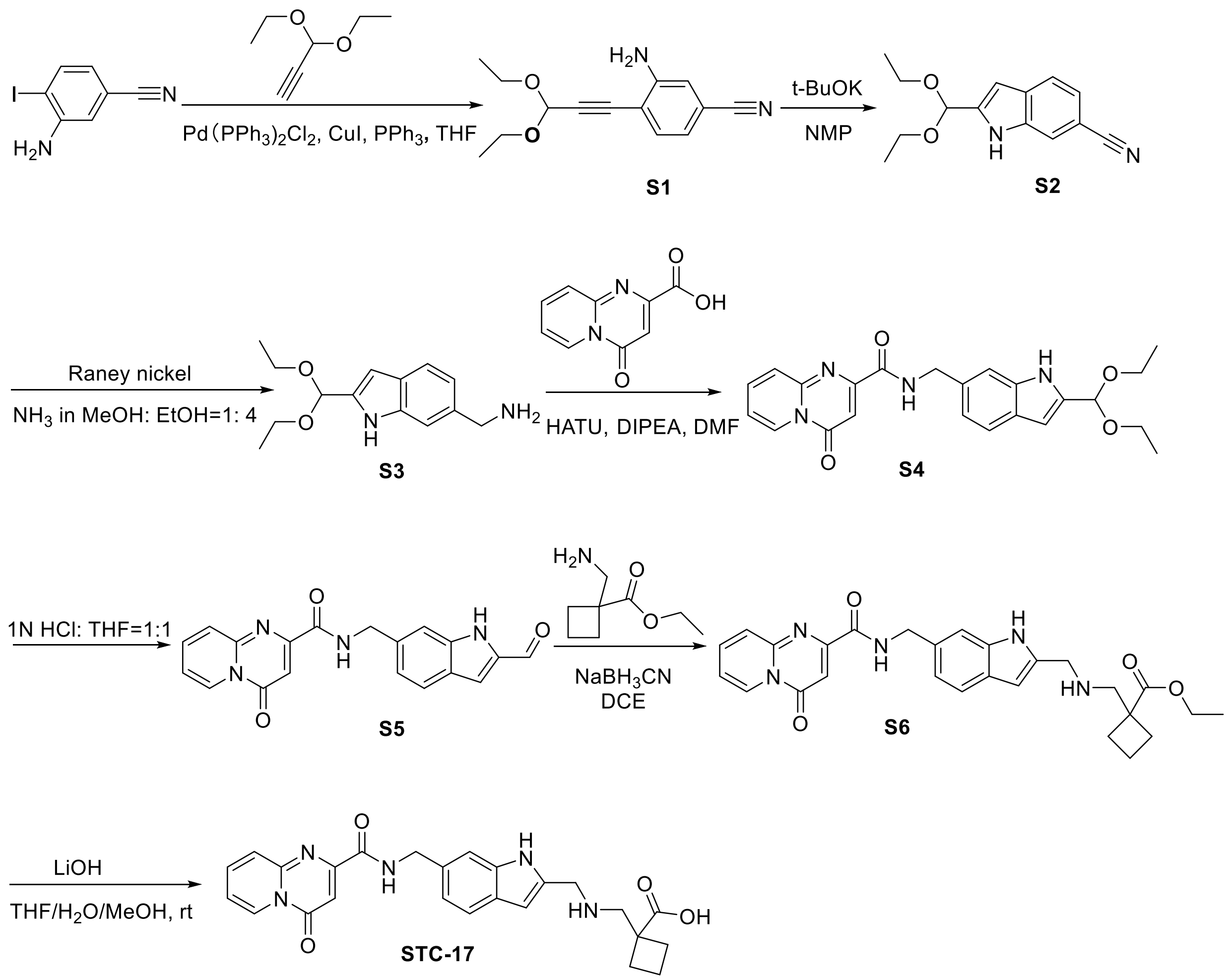

N-((2-Formyl-1H-indol-6-yl) methyl)-4-oxo-4H-pyrido[1,2-a] pyrimidine-2-carboxamide (**S5**)

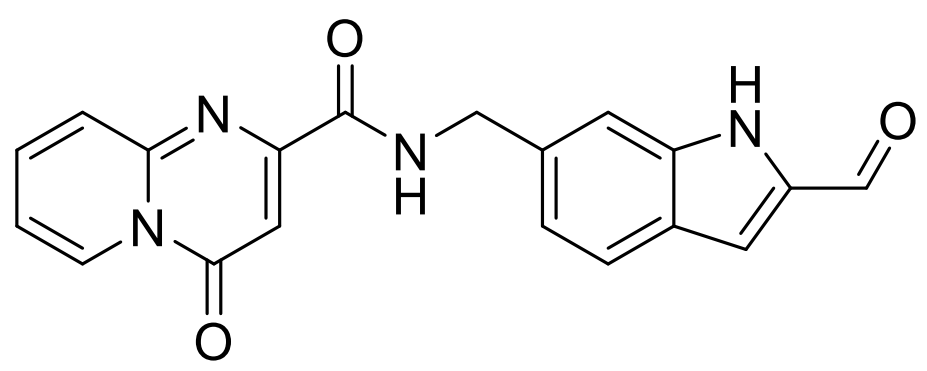

S5 (188.6 mg,0.54 mmol, 21.6 % yield over 5 steps) was prepared following a previously reported procedure[2].

LC-MS (ESI+) *m/z* calculated for $[C_{19}H_{15}N_4O_3]^+$: 347.1; found, 347.0

^{1}H NMR (700 MHz, DMSO-*d*$_6$) δ 11.91 (s, 1H), 9.81 (s, 1H), 9.43 (t, *J* = 6.4 Hz, 1H), 9.02 (d, *J* = 6.5 Hz, 1H), 8.07 (ddd, *J* = 8.5, 6.7, 1.5 Hz, 1H), 7.78 (d, *J* = 8.8 Hz, 1H), 7.70 (d, *J* = 8.3 Hz, 1H), 7.48 – 7.41 (m, 2H), 7.36 (dd, *J* = 2.0, 0.7 Hz, 1H), 7.14 (dd, *J* = 8.4, 1.3 Hz, 1H), 6.90 (s, 1H), 4.60 (d, *J* = 6.4 Hz, 2H).

^{13}C NMR (176 MHz, DMSO) δ 183.26, 163.44, 158.39, 154.35, 150.99, 138.96, 138.25, 137.00, 127.92, 126.65, 126.29, 123.44, 121.42, 117.73, 114.71, 111.96, 101.35, 43.61, 40.35, 39.64, 15.63.

Ethyl 1-((((6-((4-oxo-4H-pyrido[1,2-a] pyrimidine-2-carboxamido) methyl)-1H-indol-2-yl) methyl) amino) methyl) cyclobutane-1-carboxylate (**S6**):

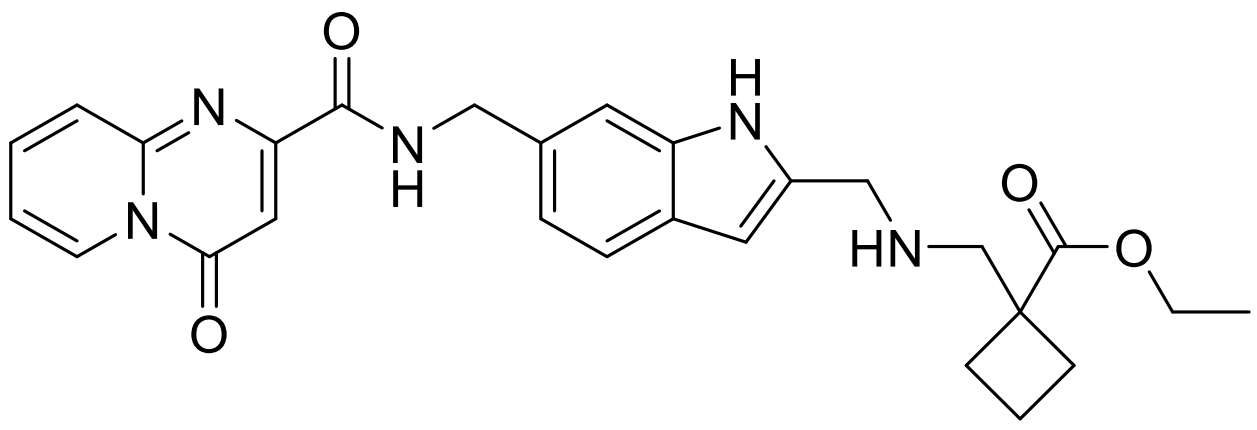

To a solution of **S5** (89.0 mg,0.26 mmol) in MeOH was added ethyl 1-(aminomethyl) cyclobutane-1-carboxylate (40.4 mg, 0.26 mmol). The reaction mixture was stirred at room temperature for 30 min, then $NaBH_3CN$ (80.7 mg, 1.28 mmol) was added portionwise. The mixture was stirred at room temperature for an additional 4 hours. Upon the completion of the reaction, as monitored by LC-MS, the mixture was quenched with water (10 mL) and the aqueous was extracted with DCM (3 × 10 mL). The combined organic phase was washed once with brine, dried over $MgSO_4$, filtered and concentrated under reduced pressure. The crude residue was purified by flash column chromatography (DCM/MeOH, 40:1 to 20:1) to afford the desired product (78.2 mg, 0.16 mmol, 62.4 % yield) as a yellow oil.

LC-MS (ESI+) *m/z* calculated for $[C_{27}H_{30}N_5O_4]^+$: 488.2; found, 488.0

^{1}H NMR (700 MHz, Chloroform-*d*) δ 9.09 – 9.06 (m, 1H), 8.68 (s, 1H), 8.29 (s, 1H), 7.76 (ddd, *J* = 9.0, 6.6, 1.6 Hz, 1H), 7.61 (s, 1H), 7.52 (d, *J* = 8.0 Hz, 1H), 7.36 (s, 1H), 7.35 (s, 1H), 7.18 (td, *J* = 6.9, 1.4 Hz, 1H), 7.09 (dd, *J* = 8.0, 1.6 Hz, 1H), 6.31 (d, *J* = 1.0 Hz, 1H), 4.74 (d, *J* = 5.8 Hz, 2H), 4.18 (q, *J* = 7.1 Hz, 2H), 3.98 (s, 2H), 2.95 (s, 2H), 2.46 – 2.38 (m, 2H), 1.98 – 1.87 (m, 4H), 1.26 (t, *J* = 7.1 Hz, 3H).

^{13}C NMR (176 MHz, $CDCl_3$) δ 176.62, 162.75, 158.68, 153.69, 150.49, 138.63, 136.75, 135.99, 130.83, 128.20, 127.59, 126.54, 120.29, 120.05, 116.03, 110.60, 102.87, 99.80, 60.65, 55.25, 47.85, 46.94, 44.49, 28.49, 15.97, 14.28.

1-((((6-((4-Oxo-4H-pyrido[1,2-a] pyrimidine-2-carboxamido) methyl)-1H-indol-2-yl) methyl) amino) methyl) cyclobutane-1-carboxylic acid (**STC-17**):

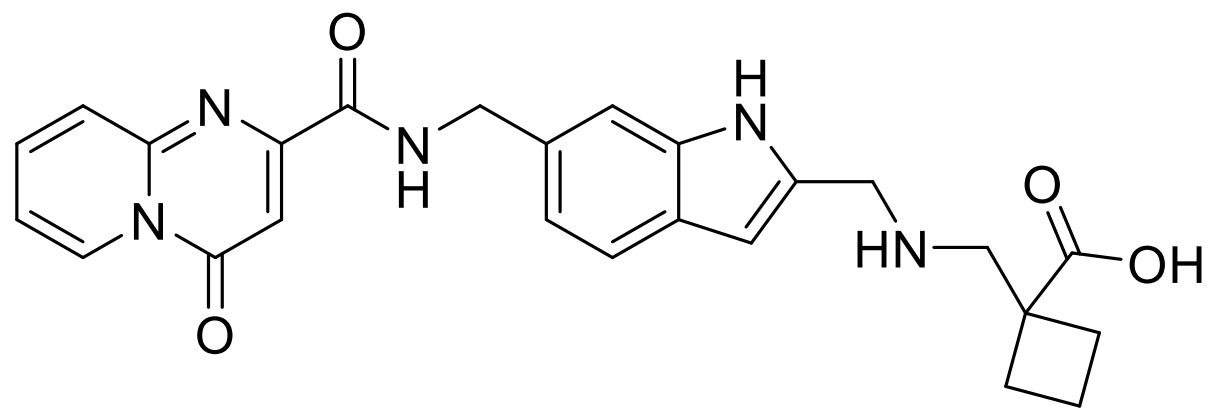

To a solution of **S6** (200.0 mg, 0.41 mmol) in a mixture of THF/MeOH/H_2O (4:1:1, 6 mL) was added LiOH·H_2O (51.5 mg, 1.23 mmol). The reaction mixture was stirred at room temperature for 4 h. Upon completion of the reaction, as monitored by LC-MS, the mixture was concentrated under reduced pressure. The residue was diluted with water (3 mL), and the aqueous solution was neutralized to pH 7 with 1 M HCl. The aqueous phase was extracted with DCM (2 × 10 mL). The combined organic phases were washed once with brine, dried over MgSO4, and concentrated under reduced pressure to afford the desired product as a yellow solid (138.0 mg, 0.3 mmol, 73.2 %), which was directly used for the next step without further purification.

LC-MS (ESI+) *m/z* calculated for $[C_{25}H_{26}N_5O_4]^+$: 460.2; found, 460.0.

Synthesis of UZH2 analogue MD-28

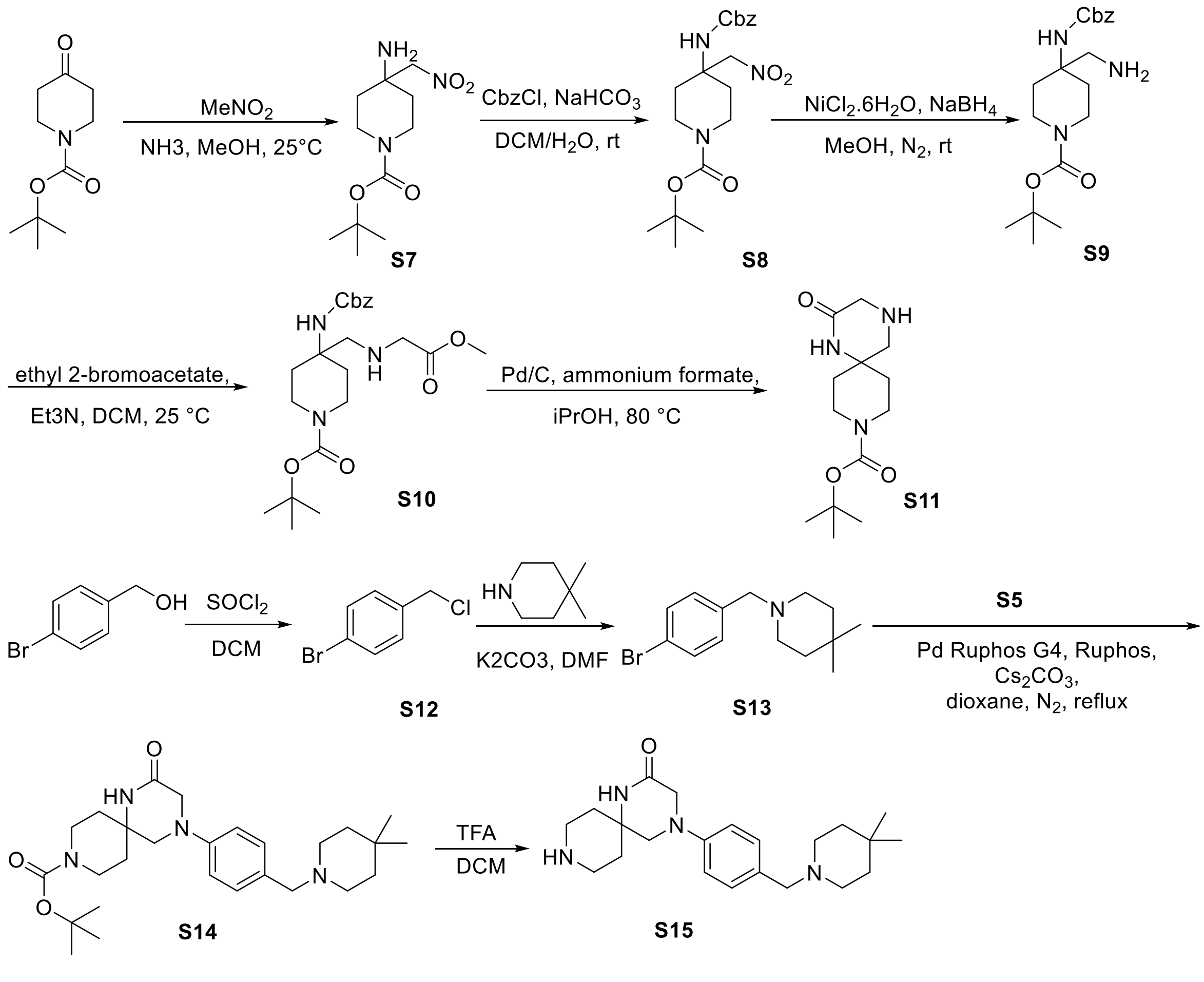

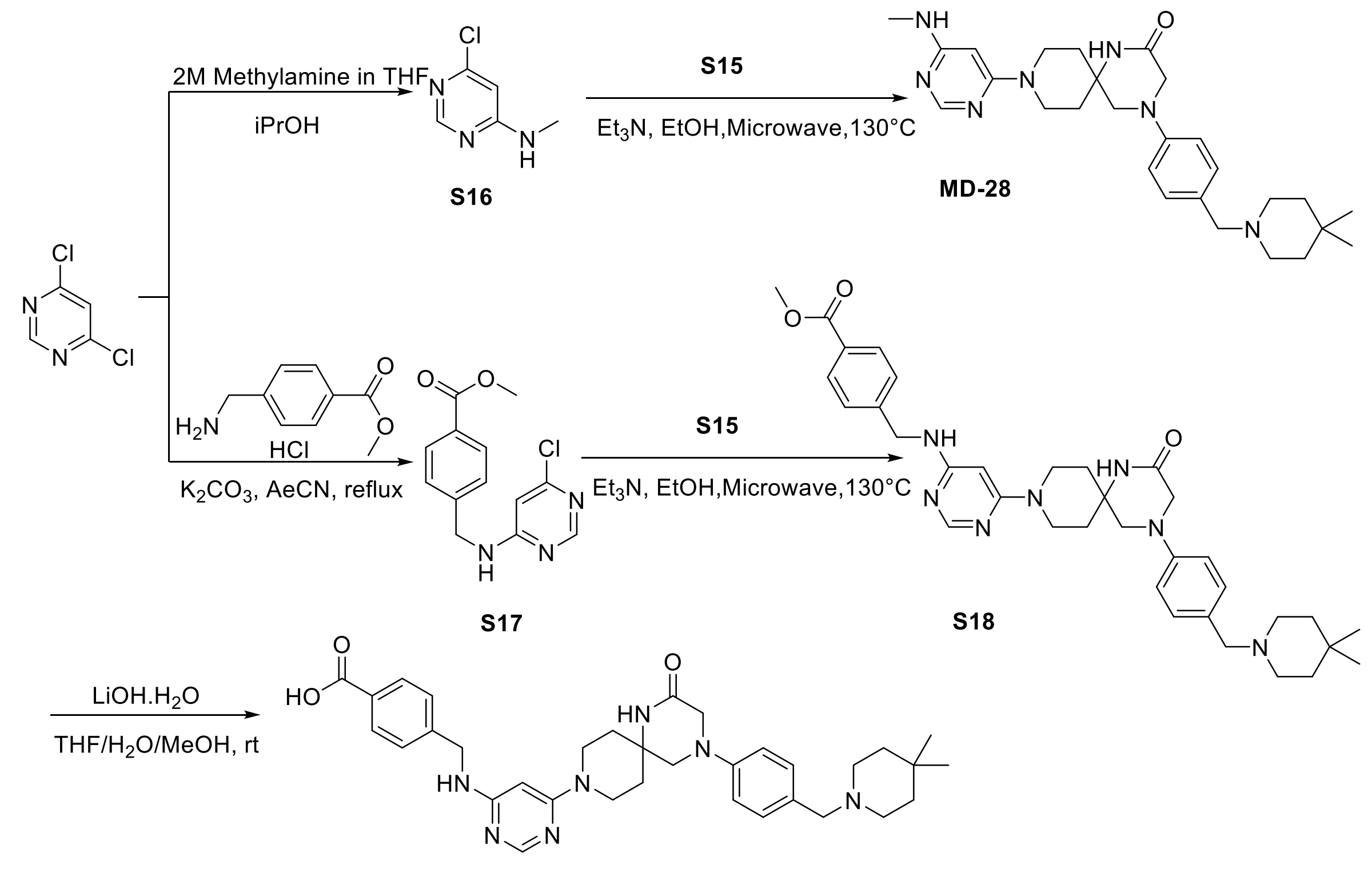

MD-41

Tert-butyl 2-oxo-1,4,9-triazaspiro [5.5] undecane-9-carboxylate (**S11**)

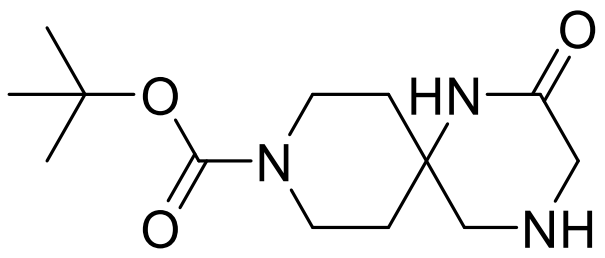

S11 (2.1 g, 7.8 mmol**,** 46.8 % yield over five steps) was synthesized according to the literature procedure[1].

LC-MS (ESI+) *m/z* calculated for $[C_{13}H_{24}N_3O_3]^+$: 270.2; found, 270.2.

^{1}H NMR (400 MHz, Chloroform-*d*) δ 6.18 (s, 1H), 3.50 (s, 2H), 3.45 (t, *J* = 5.8 Hz, 4H), 2.89 (s, 2H), 1.70 (td, *J* = 12.3, 11.3, 5.7 Hz, 4H), 1.45 (s, 9H).

4-(4-((4,4-Dimethylpiperidin-1-yl) methyl) phenyl)-1,4,9-triazaspiro [5.5] undecan-2-one (**S15**)

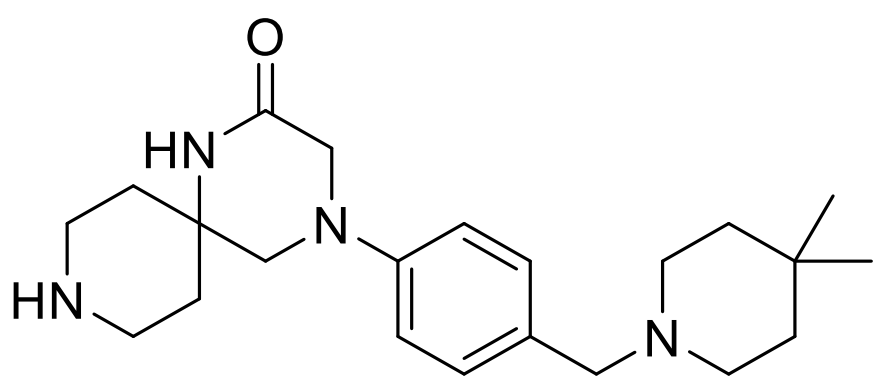

S15 (246.8 mg, 0.67 mmol, 56 %yield over four steps) was prepared following a previously reported procedure[1].

LC-MS (ESI+) *m/z* calculated for $[C_{22}H_{35}N_4O]^+$: 371.3; found, 371.0.

6-chloro-N-methylpyrimidin-4-amine (**S16**)

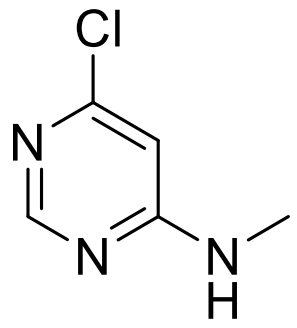

To a solution of 4,6-dichloropyrimidine (0.50 g, 3.4 mmol) in iPrOH (20 mL) at 0 °C was added 2 M methylamine in THF (4.2 mL, 8.4 mmol). The reaction mixture was allowed to warm to room temperature and stirred overnight. Upon completion of the reaction as monitored by LC–MS, water (20 mL) was added, and the mixture was extracted with EtOAc (2 × 20 mL). The combined organic extracts were washed with brine, dried over $MgSO_4$, filtered, and concentrated under reduced pressure. The crude residue was purified by flash column chromatography (petroleum ether/EtOAc = 4:1) to afford the desired product as a white solid (455.8 mg, 3.2 mmol, 94.6% yield).

LC-MS (ESI+) *m/z* calculated for $[C_5H_7ClN_3]^+$: 144.0; found, 144.0.

^{1}H NMR (400 MHz, DMSO-d_6) δ 8.24 (s, 1H), 7.63 (s, 1H), 6.46 (d, *J* = 0.9 Hz, 1H), 2.79 (s, 3H).

4-(4-((4,4-dimethylpiperidin-1-yl) methyl) phenyl)-9-(6-(methylamino) pyrimidin-4-yl)-1,4,9-triazaspiro [5.5] undecan-2-one (**MD-28**):

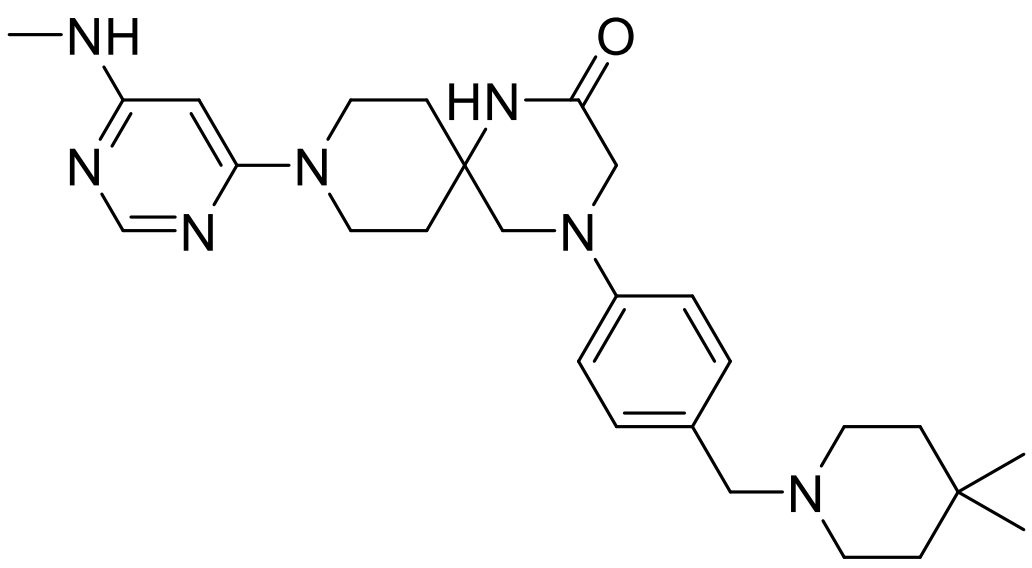

To a solution of **S15** (100.0 mg, 0.27 mmol) and **S16** (38.7 mg, 0.27 mmol) in EtOH was added TEA (174.4 mg, 1.35 mmol). The reaction mixture was heated at 130 °C under microwave irradiation for 6 h. Upon completion of the reaction, as monitored by LC-MS, the mixture was concentrated under reduced pressure, and the crude residue was purified by flash column chromatography (DCM/MeOH = 40:1) to afford the desired compound as a light yellow solid (68.9 mg, 144.3 μmol, 53.5 % yield).

LC-MS (ESI+) *m/z* calculated for $[C_{27}H_{40}N_7O]^+$: 478.3; found, 478.0.

^{1}H NMR (700 MHz, Methanol-d_4) δ 8.07 (s, 1H), 7.30 (d, *J* = 8.8 Hz, 2H), 6.95 (d, *J* = 8.8 Hz, 2H), 5.72 (s, 1H), 4.12 (s, 2H), 3.80 (s, 2H), 3.47 (s, 2H), 3.19 (s, 1H), 2.99 (dt,

J = 12.9, 8.6 Hz, 2H), 2.85 (s, 3H), 1.87 (dd, J = 11.6, 6.9 Hz, 2H), 1.80 – 1.75 (m, 2H), 1.53 (dd, J = 6.8, 3.0 Hz, 4H), 0.98 (s, 3H), 0.94 (s, 3H).

Methyl 4-(((6-chloropyrimidin-4-yl) amino) methyl) benzoate (**S17**):

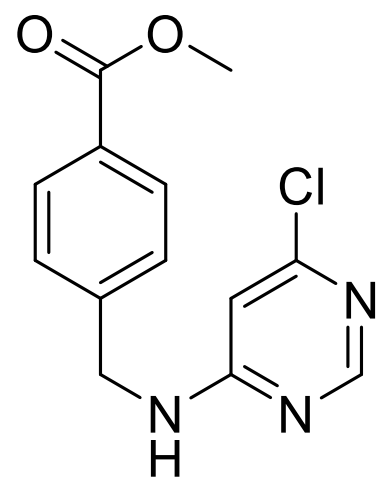

To a solution of (4-(methoxycarbonyl) phenyl) methanaminium hydrochloride (1.0 g, 5.0 mmol) in acetonitrile (30 mL) were added 4,6-dichloropyrimidine (1.5 g, 10.0 mmol) and K_2CO_3 (2.05 g, 14.9 mmol). The reaction mixture was stirred at reflux overnight. Upon completion of the reaction, as monitored by LC-MS, the mixture was diluted with water (100 mL). The aqueous layer was extracted with ethyl acetate (2 × 50 mL). The combined organic extracts were washed with brine, dried over $MgSO_4$, filtered, and concentrated under reduced pressure. The crude residue was purified by flash column chromatography (PE/EA = 2:1) to afford the desired product as a white solid (1.32 g, 4.75 mmol, 95.8%).
LC-MS (ESI+) *m/z* calculated for $[C_{13}H_{13}ClN_3O_2]^+$: 278.1; found, 278.0.

Methyl 4-(((6-(4-(4-((4,4-dimethylpiperidin-1-yl) methyl) phenyl)-2-oxo-1,4,9-triazaspiro [5.5] undecan-9-yl) pyrimidin-4-yl) amino) methyl) benzoate (**S18**):

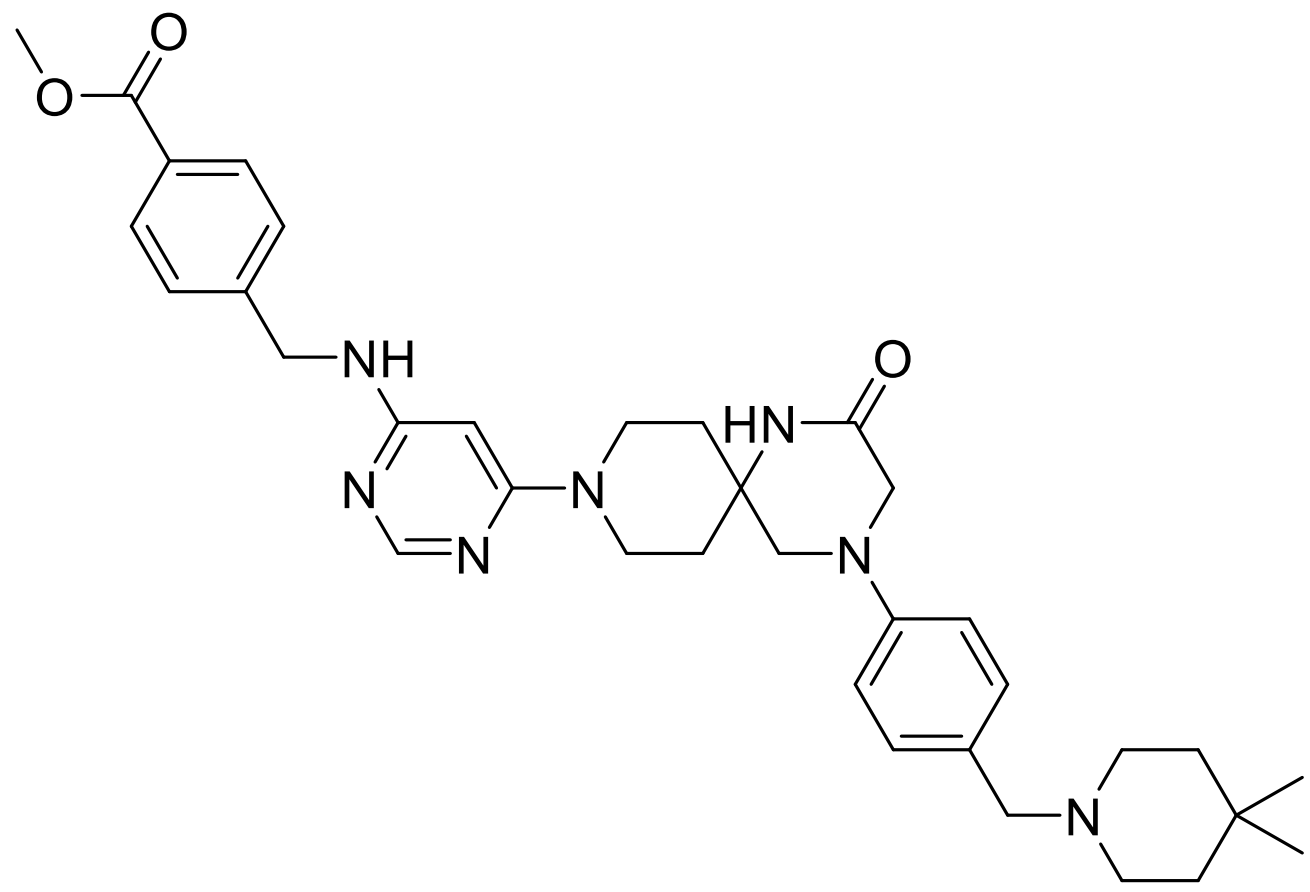

To a solution of **S15** (100.0 mg, 0.27 mmol) and **S17** (89.9 mg, 0.32 mmol) in EtOH was added TEA (136.5 mg, 1.35 mmol). The reaction mixture was heated at 130 °C under microwave irradiation for 6 h. Upon completion of the reaction, as monitored by

LC-MS, the mixture was concentrated under reduced pressure, and the crude residue was purified by flash column chromatography (DCM/MeOH = 40:1) to afford the desired compound as a light yellow solid (98.7 mg, 0.16 mmol, 59.8%).

LC-MS (ESI+) *m/z* calculated for $[C_{35}H_{46}N_7O_3]^+$: 612.4; found, 612.0.

4-(((6-(4-(4-((4,4-Dimethylpiperidin-1-yl) methyl) phenyl)-2-oxo-1,4,9-triazaspiro [5.5] undecan-9-yl) pyrimidin-4-yl) amino) methyl) benzoic acid (**MD-41**):

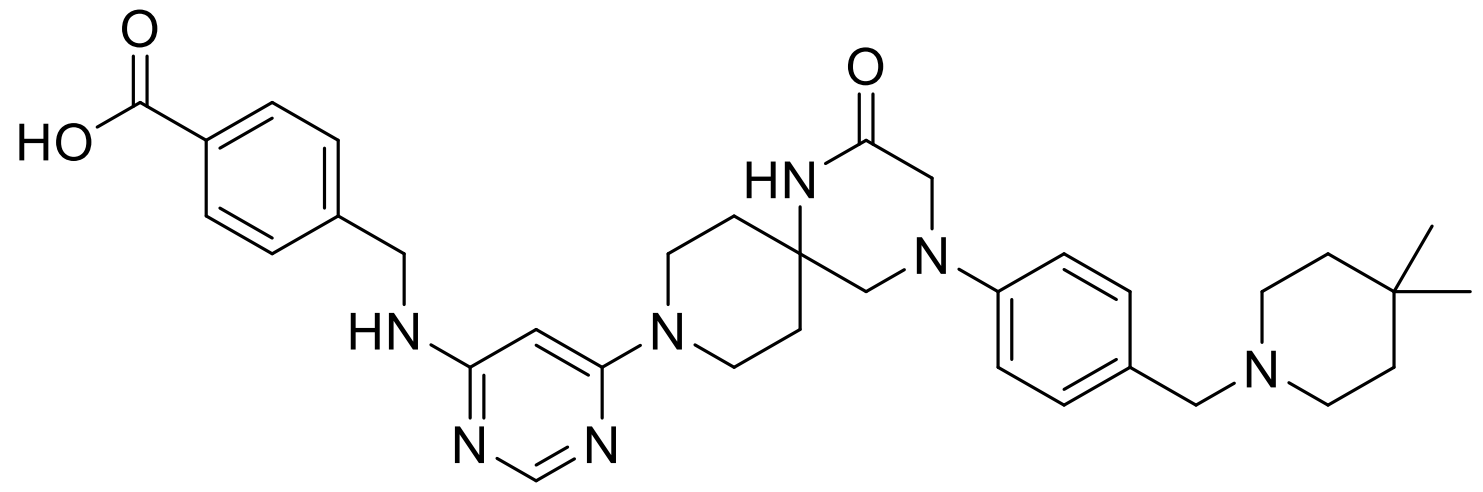

To a solution of **S18** (90.0 mg, 0.15 mmol) in a mixture of THF/MeOH/H_2O (4:1:1, 6 mL) was added LiOH·H_2O (9.3 mg, 0.22 mmol). The reaction mixture was stirred at room temperature for 4 h. Upon completion of the reaction, as monitored by LC-MS, the mixture was concentrated under reduced pressure. The residue was diluted with water (1 mL), and the aqueous solution was acidified to pH 4 with 1 M HCl. A pale yellow solid precipitated from the aqueous phase. The resulting suspension was filtered, and the collected solid was dried to afford the title compound as a light-yellow solid (49.0 mg, 0.08 mmol, 55.7%).

LC-MS (ESI+) *m/z* calculated for $[C_{34}H_{44}N_7O_3]^+$: 598.4; found, 598.0.

Synthesis *of E3 ligase ligand with linkers*

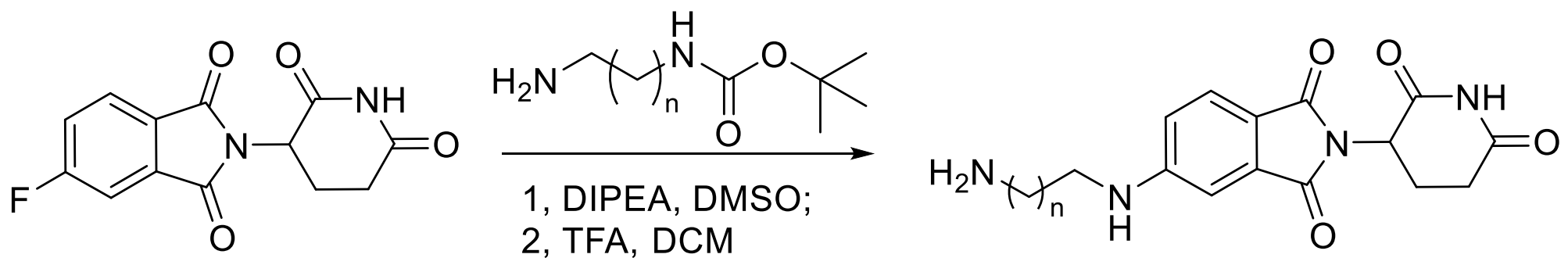

L01, n=1;
L02, n=2;
L03, n=3;
L04, n=4;
L05, n=5;
L06, n=6;
L07, n=7;
L08, n=8.

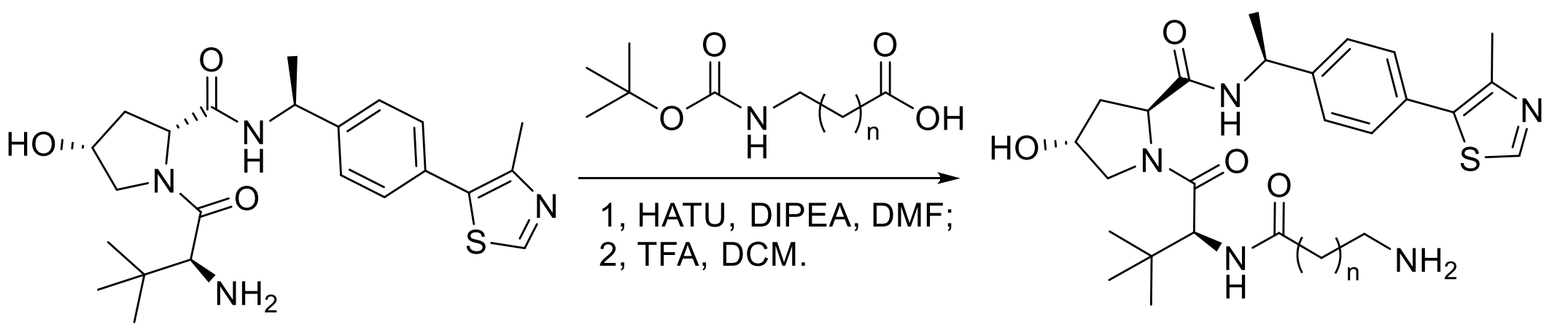

L09, n=1;
L10, n=2;
L11, n=4;
L12, n=8;

General Procedure A (Nucleophilic aromatic substitution and Boc group de-protection):

The corresponding amine linker (1.0 equiv.) was reacted with 2-(2,6-dioxopiperidin-3-yl)-5-fluoroisoindoline-1,3-dione (1.0 equiv.) in DMSO in the presence of DIPEA (3.0 equiv.) at 130 °C for 3 h. After removal of the solvent, the residue was dissolved in DCM and treated with TFA at room temperature for 3 h. Concentration under reduced pressure afforded the crude product, which was carried forward to the next step without purification.

General Procedure B (acid-amine coupling and Boc group de-protection):

To a solution of the corresponding carboxylic acid linker (1.0 equiv.) in DMF (5 mL) were added HATU (1.5 equiv.) and DIPEA (5.0 equiv.). After stirring at room temperature for 5 min, (2S,4R)-1-((S)-2-amino-3,3-dimethylbutanoyl)-4-hydroxy-N-((S)-1-(4-(4-methylthiazol-5-yl) phenyl) ethyl) pyrrolidine-2-carboxamide (1.0 equiv.) was added. The reaction mixture was stirred at room temperature for 3 h. Upon completion of the reaction as monitored by LC–MS, water (50 mL) was added, and the mixture was extracted with EtOAc (3 × 20 mL). The combined organic extracts were washed with brine, dried over $MgSO_4$, filtered, and concentrated under reduced pressure. The resulting residue was dissolved in DCM (5 mL) and treated with TFA (1.5 mL). The reaction mixture was stirred at room temperature for 3 h. After completion of the reaction as confirmed by LC–MS, the solvent was removed under reduced pressure to afford the crude product, which was used directly in the next step without further purification.

5-((2-Aminoethyl) amino)-2-(2,6-dioxopiperidin-3-yl) isoindoline-1,3-dione (**L01**)

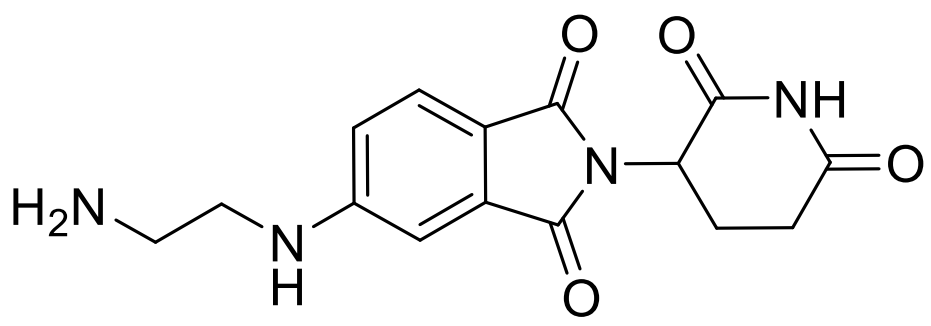

Following **General Procedure A**, **L01** was obtained as a yellow solid (78.6 mg, 0.25 mmol, 69.1 % over two steps).

LC-MS (ESI+) *m/z* calculated for $[C_{15}H_{17}N_4O_4]^+$: 317.1; found, 317.0.

5-((3-Aminopropyl) amino)-2-(2,6-dioxopiperidin-3-yl) isoindoline-1,3-dione (**L02**)

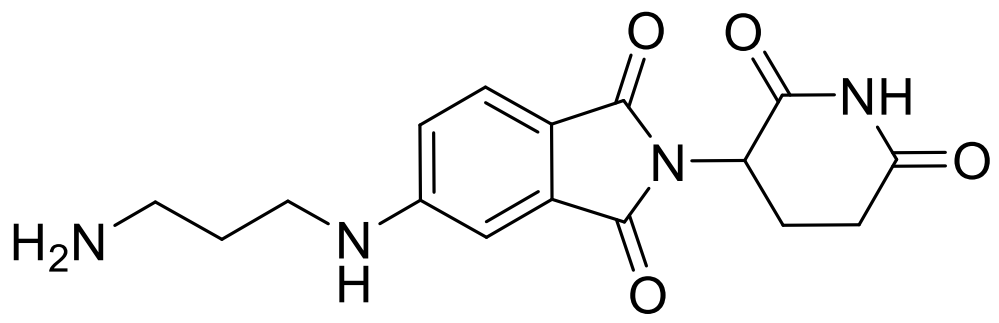

Following **General Procedure A**, **L02** was obtained as a yellow solid (68.3 mg, 0.21 mmol, 57.2 % over two steps).

LC-MS (ESI+) *m/z* calculated for $[C_{16}H_{19}N_4O_4]^+$: 331.1; found, 331.0.

5-((4-Aminobutyl) amino)-2-(2,6-dioxopiperidin-3-yl) isoindoline-1,3-dione (**L03**)

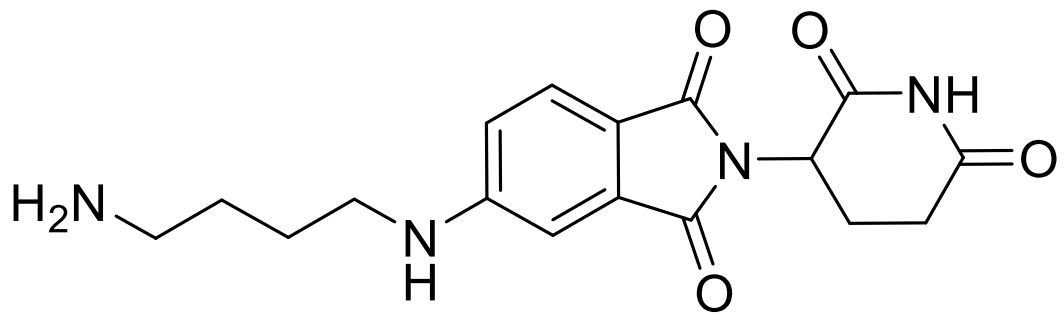

Following **General Procedure A**, **L03** was obtained as a yellow oil (60.4 mg, 0.17 mmol, 48.5 % over two steps).

LC-MS (ESI+) *m/z* calculated for $[C_{17}H_{21}N_4O_4]^+$: 345.2; found, 345.0.

5-((5-Aminopentyl) amino)-2-(2,6-dioxopiperidin-3-yl) isoindoline-1,3-dione (**L04**)

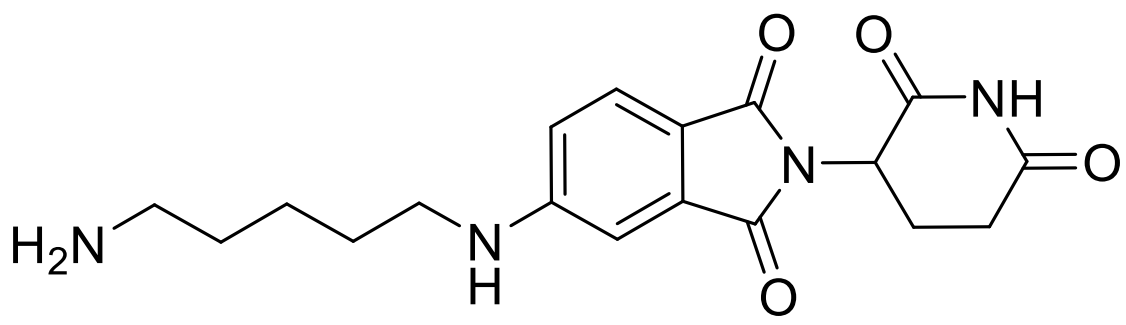

Following **General Procedure A**, **L04** was obtained as a yellow oil (82.4 mg, 0.23 mmol, 63.5 % over two steps).

LC-MS (ESI+) *m/z* calculated for $[C_{18}H_{23}N_4O_4]^+$: 359.2; found, 359.2.

5-((6-Aminohexyl) amino)-2-(2,6-dioxopiperidin-3-yl) isoindoline-1,3-dione (**L05**)

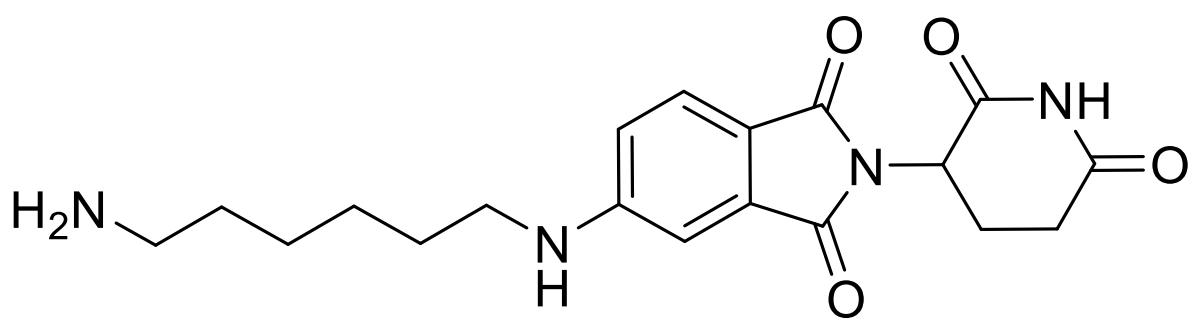

Following **General Procedure A**, **L05** was obtained as a yellow oil (66.2 mg, 0.18 mmol, 49.1 % over two steps).

LC-MS (ESI+) *m/z* calculated for $[C_{19}H_{25}N_4O_4]^+$: 373.2; found, 373.2.

5-((7-Aminoheptyl) amino)-2-(2,6-dioxopiperidin-3-yl) isoindoline-1,3-dione (**L06**)

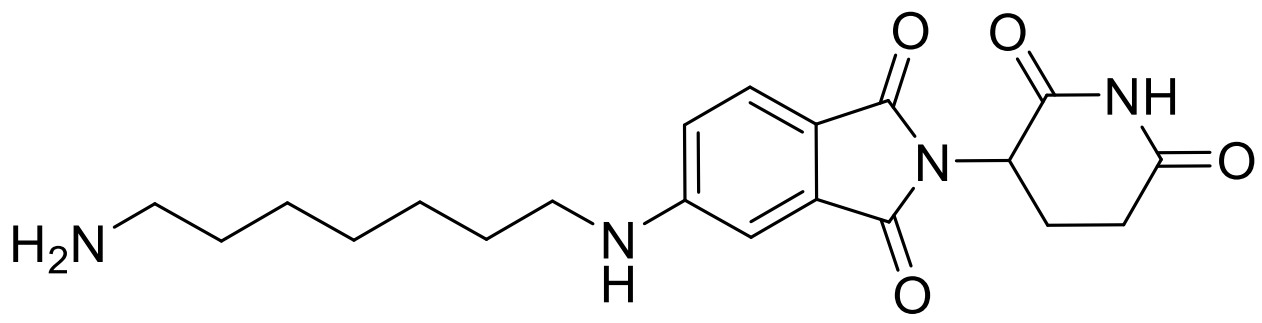

Following **General Procedure A**, **L06** was obtained as a yellow oil (90.5 mg, 0.23 mmol, 64.6 % over two steps).

LC-MS (ESI+) *m/z* calculated for $[C_{20}H_{27}N_4O_4]^+$: 387.2; found, 387.2.

5-((8-Aminooctyl) amino)-2-(2,6-dioxopiperidin-3-yl) isoindoline-1,3-dione (**L07**)

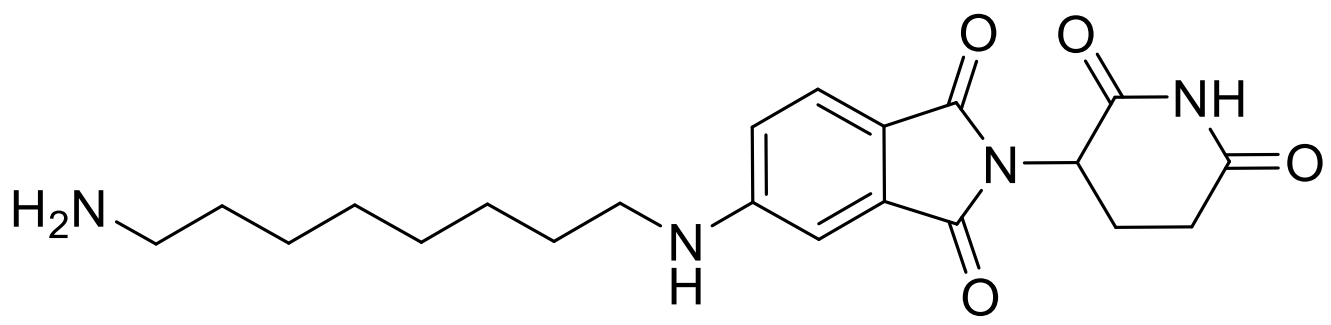

Following **General Procedure A**, **L07** was obtained as a yellow oil (100.7 mg, 0.25 mmol, 69.1 % over two steps).

LC-MS (ESI+) *m/z* calculated for $[C_{21}H_{29}N_4O_4]^+$: 401.2; found, 401.0.

5-((9-Aminononyl) amino)-2-(2,6-dioxopiperidin-3-yl) isoindoline-1,3-dione (**L08**)

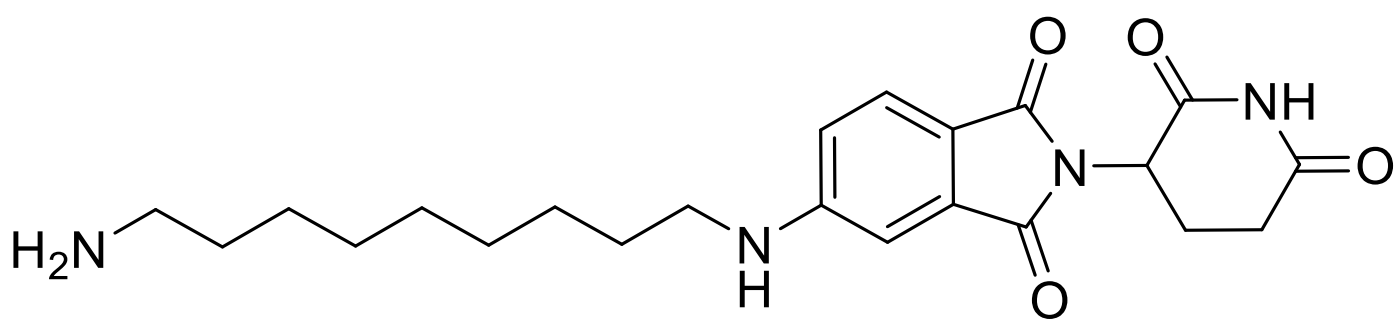

Following **General Procedure A**, **L08** was obtained as a yellow oil (128.9 mg, 0.31 mmol, 85.6 % over two steps).

LC-MS (ESI+) *m/z* calculated for $[C_{22}H_{31}N_4O_4]^+$: 415.2; found, 415.0.

(2S,4R)-1-((S)-2-(3-Aminopropanamido)-3,3-dimethylbutanoyl)-4-hydroxy-N-((S)-1-(4-(4-methylthiazol-5-yl) phenyl) ethyl) pyrrolidine-2-carboxamide (**L09**)

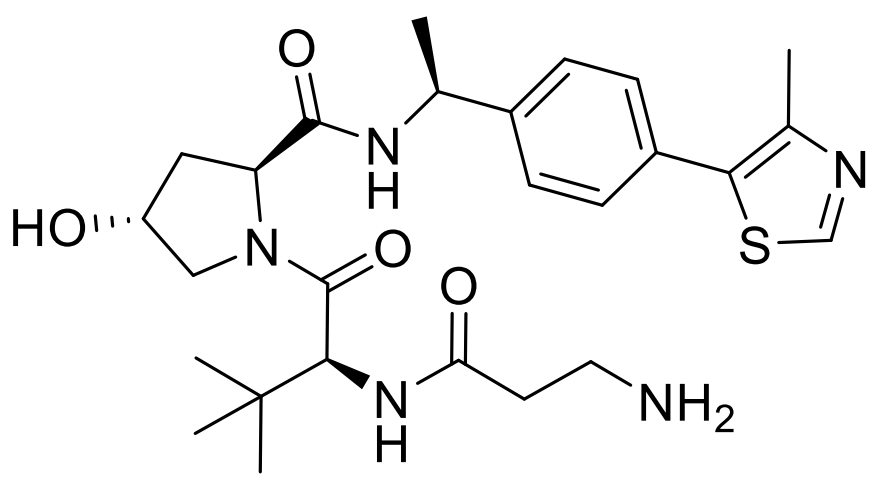

Following **General Procedure B**, **L09** was obtained as a colorless oil (77.2 mg, 0.15 mmol, 66.5 % over two steps).

LC-MS (ESI+) *m/z* calculated for $[C_{26}H_{38}N_5O_4S]^+$: 516.3; found, 516.0.

(2S,4R)-1-((S)-2-(4-Aminobutanamido)-3,3-dimethylbutanoyl)-4-hydroxy-N-((S)-1-(4-(4-methylthiazol-5-yl) phenyl) ethyl) pyrrolidine-2-carboxamide (**L10**)

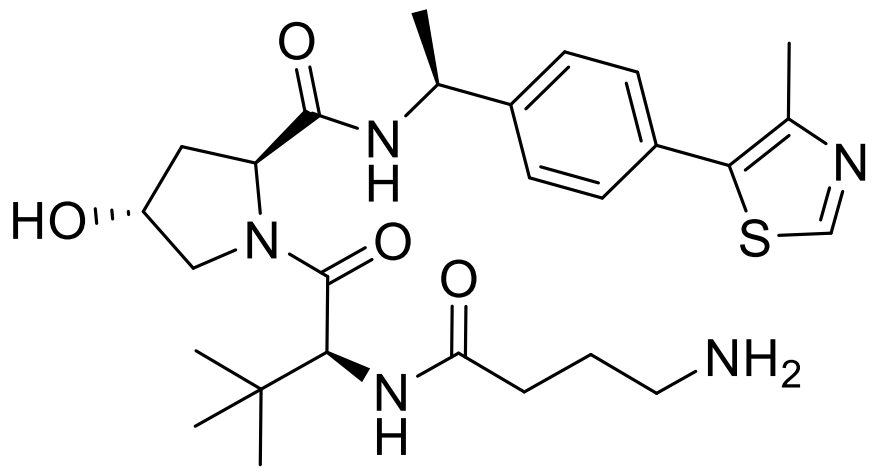

Following **General Procedure B**, **L10** was obtained as a colorless oil (84.7 mg, 0.16 mmol, 71.1 % over two steps).

LC-MS (ESI+) *m/z* calculated for $[C_{27}H_{40}N_5O_4S]^+$: 530.3; found, 530.3.

(2S,4R)-1-((S)-2-(6-Aminohexanamido)-3,3-dimethylbutanoyl)-4-hydroxy-N-((S)-1-(4-(4-methylthiazol-5-yl) phenyl) ethyl) pyrrolidine-2-carboxamide (**L11**)

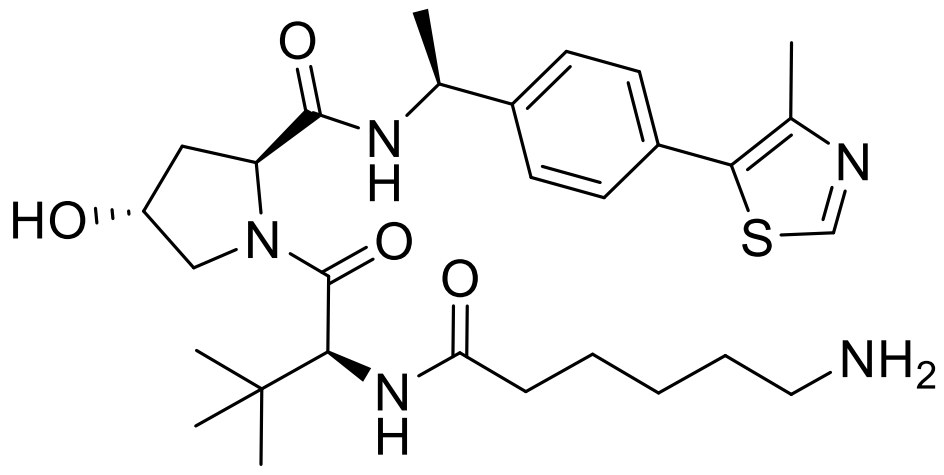

Following **General Procedure B**, **L11** was obtained as a colorless oil (97.6 mg, 0.17 mmol, 75.6 % over two steps).

LC-MS (ESI+) *m/z* calculated for $[C_{29}H_{44}N_5O_4S]^+$: 558.3; found, 558.3.

(2S,4R)-1-((S)-2-(10-Aminodecanamido)-3,3-dimethylbutanoyl)-4-hydroxy-N-((S)-1-(4-(4-methylthiazol-5-yl) phenyl) ethyl) pyrrolidine-2-carboxamide (**L12**)

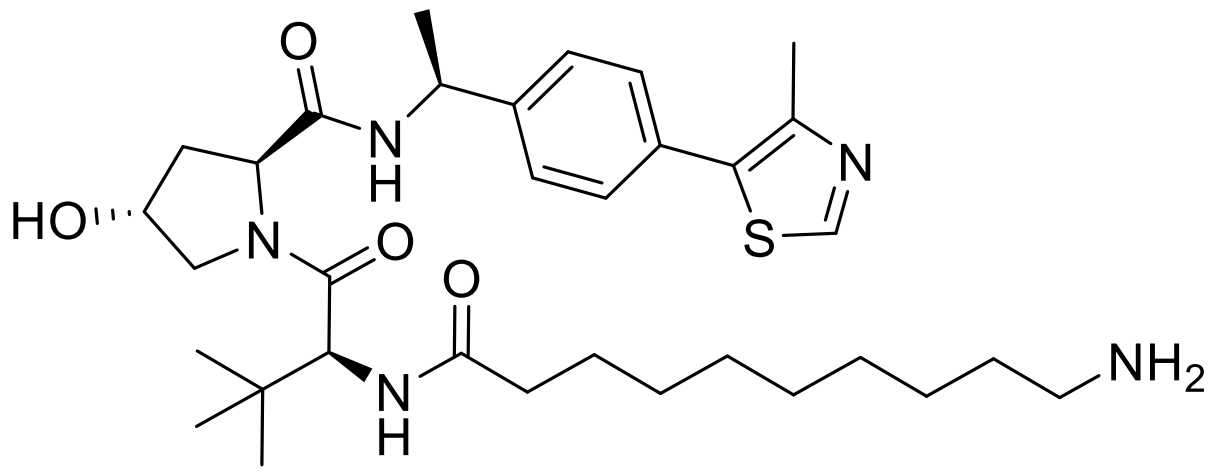

Following **General Procedure B**, **L12** was obtained as a colorless oil (110.7 mg, 0.18 mmol, 80.1 % over two steps).

LC-MS (ESI+) *m/z* calculated for $[C_{33}H_{52}N_5O_4S]^+$: 614.4; found, 614.2.

Synthesis of METTL3 PROTACs

General Purification Procedure for METTL3 PROTACs

Preparative HPLC purification was performed on a BUCHI C-850 Prep-Flash system equipped with a VP 125/21 NUCLEODUR C18 Gravity column (5 μm). UV absorbance was monitored at 210, 254, 280, and 345 nm. Compounds were purified using a linear gradient of 0–100% acetonitrile in water containing 0.1% (v/v) TFA over 120 min at a flow rate of 20 mL/min.

General Procedure C (Amide Coupling)

Regarding **STC-17** series compounds:

To a solution of the corresponding carboxylic acid (1.0 equiv.) and amine (1.0 equiv.) in DMF (5 mL) at 0 °C were added HATU (1.5 equiv.) and DIPEA (3.0 equiv.). The reaction mixture was stirred at 0 °C for 1 h to suppress the formation of intramolecularly cyclized by-products. Upon completion of the reaction as monitored by LC–MS, the solvent was removed under reduced pressure. The crude residue was purified according to the **General Purification Procedure for METTL3 PROTACs** to afford the desired product.

Regarding **MD-28** Series compounds:

To a solution of the corresponding carboxylic acid (1.0 equiv.) in DMF (5 mL) were added HATU (1.5 equiv.) and DIPEA (3.0 equiv.). After stirring at room temperature for 5 min, the corresponding amine (1.0 equiv.) was added. The reaction mixture was stirred at room temperature for 3 h. Upon completion of the reaction as monitored by LC–MS, the solvent was removed under reduced pressure. The crude residue was purified according to the **General Purification Procedure for METTL3 PROTACs** to afford the desired product.

Regarding STC series compounds:

.*N*-((2-((((1-((2-((2-(2,6-Dioxopiperidin-3-yl)-1,3-dioxoisoindolin-5-yl) amino) ethyl) carbamoyl) cyclobutyl) methyl) amino) methyl)-1H-indol-6-yl) methyl)-4-oxo-4H-pyrido [1,2-a] pyrimidine-2-carboxamide (**1**)

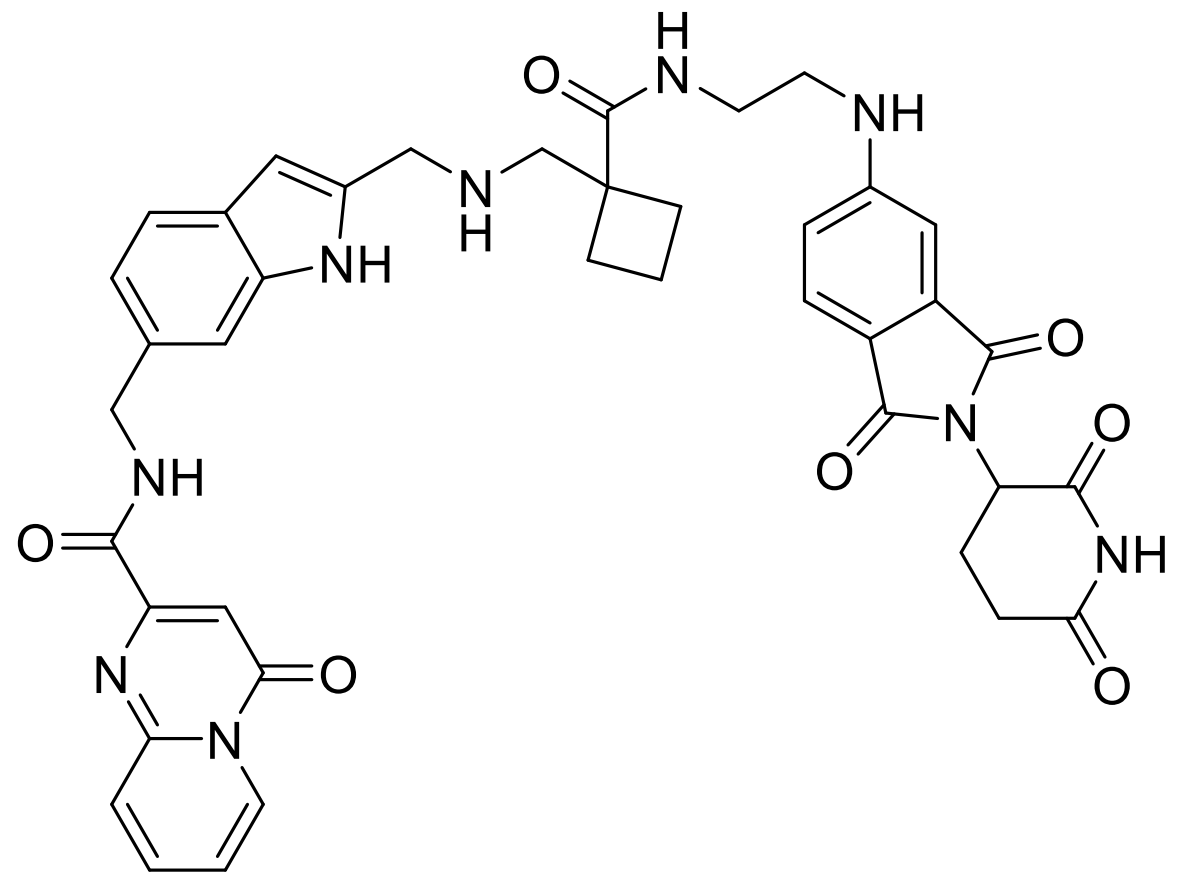

STC-17 (10.0 mg, 21.8 μmol) and **L01** (6.9 mg, 21.8 μmol) were coupled according to **General Procedure C** and purified according to the **General Purification Procedure for METTL3 PROTACs** to afford the title compound as a yellow solid (3.85 mg, 5.1 μmol, 23.0 % yield).

HRMS: Calculated for $[C_{40}H_{40}N_9O_7]^+$: 758.3045; found, 758.3055.

^{1}H NMR (700 MHz, Acetone-d_6) δ 8.91 (dt, J = 7.1, 1.2 Hz, 1H), 7.95 – 7.88 (m, 1H), 7.64 – 7.58 (m, 1H), 7.36 – 7.29 (m, 4H), 6.94 (d, J = 10.0 Hz, 2H), 6.84 (d, J = 2.2 Hz, 1H), 6.69 (dd, J = 8.4, 2.2 Hz, 1H), 6.16 (s, 1H), 4.92 (dd, J = 12.6, 5.5 Hz, 1H), 4.57 (d, J = 11.5 Hz, 2H), 3.79 (s, 2H), 3.32 (t, J = 6.3 Hz, 2H), 3.19 (t, J = 6.3 Hz, 2H), 2.89 – 2.81 (m, 1H), 2.79 (s, 2H), 2.70 – 2.57 (m, 2H), 2.20 (tt, J = 10.5, 4.0 Hz, 2H), 2.04 (p, J = 2.2 Hz, 2H), 1.86 (p, J = 2.2 Hz, 1H), 1.73 (t, J = 3.7 Hz, 3H), 1.68 – 1.61 (m, 1H).

N-((2-((((1-((4-((2-(2,6-Dioxopiperidin-3-yl)-1,3-dioxoisoindolin-5-yl) amino) butyl) carbamoyl) cyclobutyl) methyl) amino) methyl)-1H-indol-6-yl) methyl)-4-oxo-4H-pyrido [1,2-a] pyrimidine-2-carboxamide (**2**)

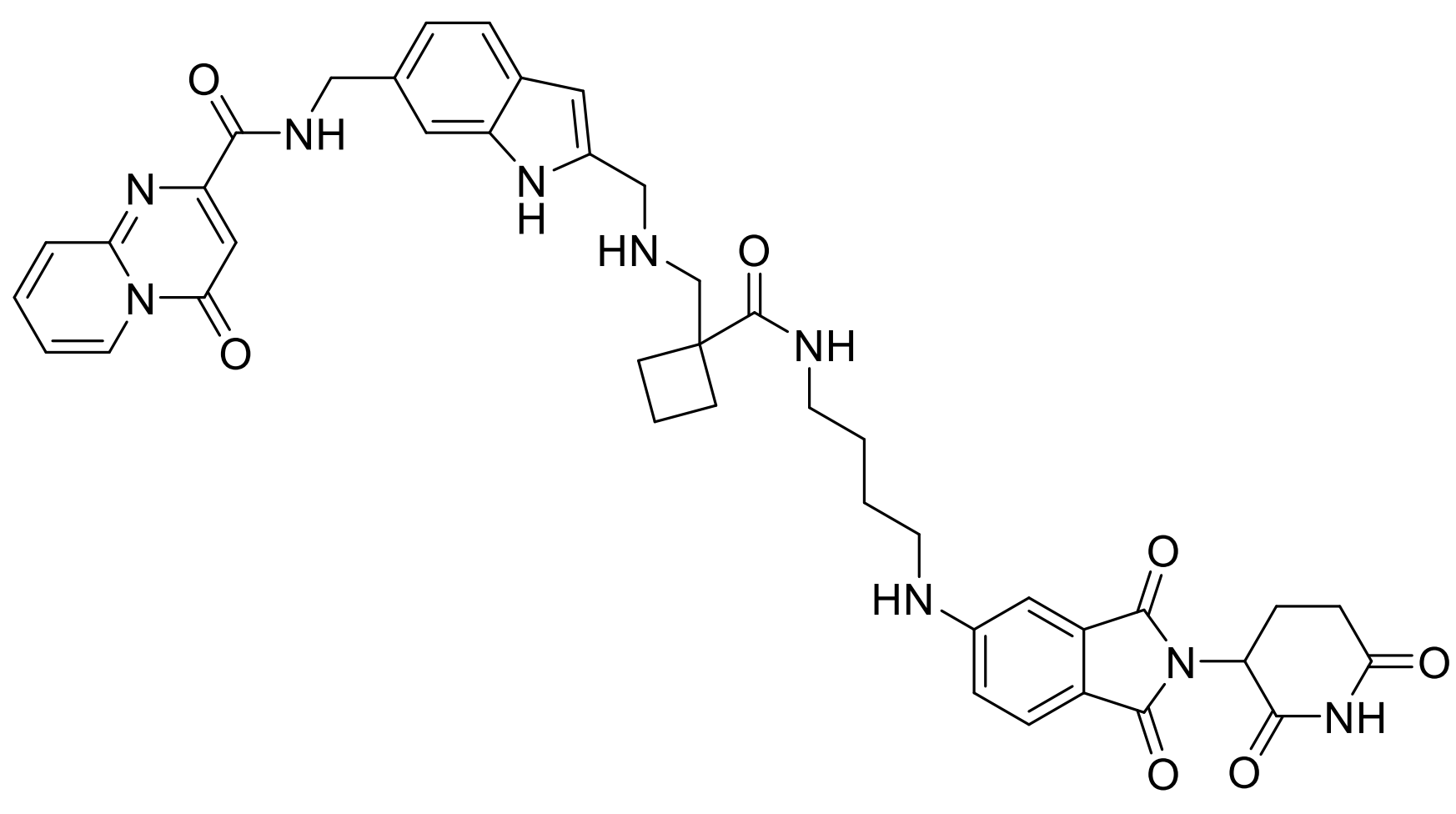

STC-17 (10.0 mg, 21.8 µmol) and **L03** (9.0 mg, 21.8 µmol) were coupled according to **General Procedure C** and purified according to the **General Purification Procedure for METTL3 PROTACs** to afford the title compound as a yellow solid (2.61 mg, 3.3 µmol, 15.3 % yield).

HRMS: Calculated for $[C_{42}H_{44}N_9O_7]^+$: 786.3358; found, 786.3344.

^{1}H NMR (500 MHz, Methanol-d_4) δ 8.92 (dt, *J* = 6.9, 1.2 Hz, 1H), 7.85 (ddd, *J* = 8.5, 6.7, 1.5 Hz, 1H), 7.66 (dt, *J* = 9.0, 1.1 Hz, 1H), 7.43 (d, *J* = 8.2 Hz, 1H), 7.33 (d, *J* = 8.5 Hz, 2H), 7.27 (td, *J* = 7.0, 1.4 Hz, 1H), 7.02 (dd, *J* = 8.2, 1.5 Hz, 1H), 6.95 (s, 1H), 6.72 (d, *J* = 2.1 Hz, 1H), 6.57 (dd, *J* = 8.4, 2.2 Hz, 1H), 6.55 (d, *J* = 0.8 Hz, 1H), 4.91 (dd, *J* = 12.7, 5.6 Hz, 1H), 4.58 (s, 2H), 4.30 (s, 2H), 3.25 (s, 2H), 3.08 (s, 2H), 2.94 (s, 2H), 2.74 (ddd, *J* = 17.4, 13.9, 5.3 Hz, 1H), 2.67 – 2.53 (m, 2H), 2.30 – 2.23 (m, 2H), 1.98 (ddd, *J* = 9.9, 5.2, 2.7 Hz, 1H), 1.94 – 1.89 (m, 3H), 1.87 – 1.80 (m, 1H), 1.42 – 1.35 (m, 4H).

^{13}C NMR (126 MHz, MeOD) δ 175.87, 173.26, 170.39, 168.26, 167.79, 165.42, 164.59, 163.87, 161.00, 159.18, 154.48, 154.00, 150.80, 137.76, 137.21, 134.50, 133.12, 127.50, 127.28, 127.09, 126.31, 124.63, 120.58, 119.98, 117.00, 116.60, 110.45, 104.71, 101.09, 51.55, 48.90, 44.70, 44.29, 43.49, 42.09, 38.67, 30.80, 27.92, 26.29, 25.35, 22.45, 14.68.

N-((2-((((1-((6-((2-(2,6-Dioxopiperidin-3-yl) -1,3-dioxoisoindolin-5-yl) amino) hexyl) carbamoyl) cyclobutyl) methyl) amino) methyl) -1H-indol-6-yl) methyl) -4-oxo-4H-pyrido [1,2-a] pyrimidine-2-carboxamide (**3**)

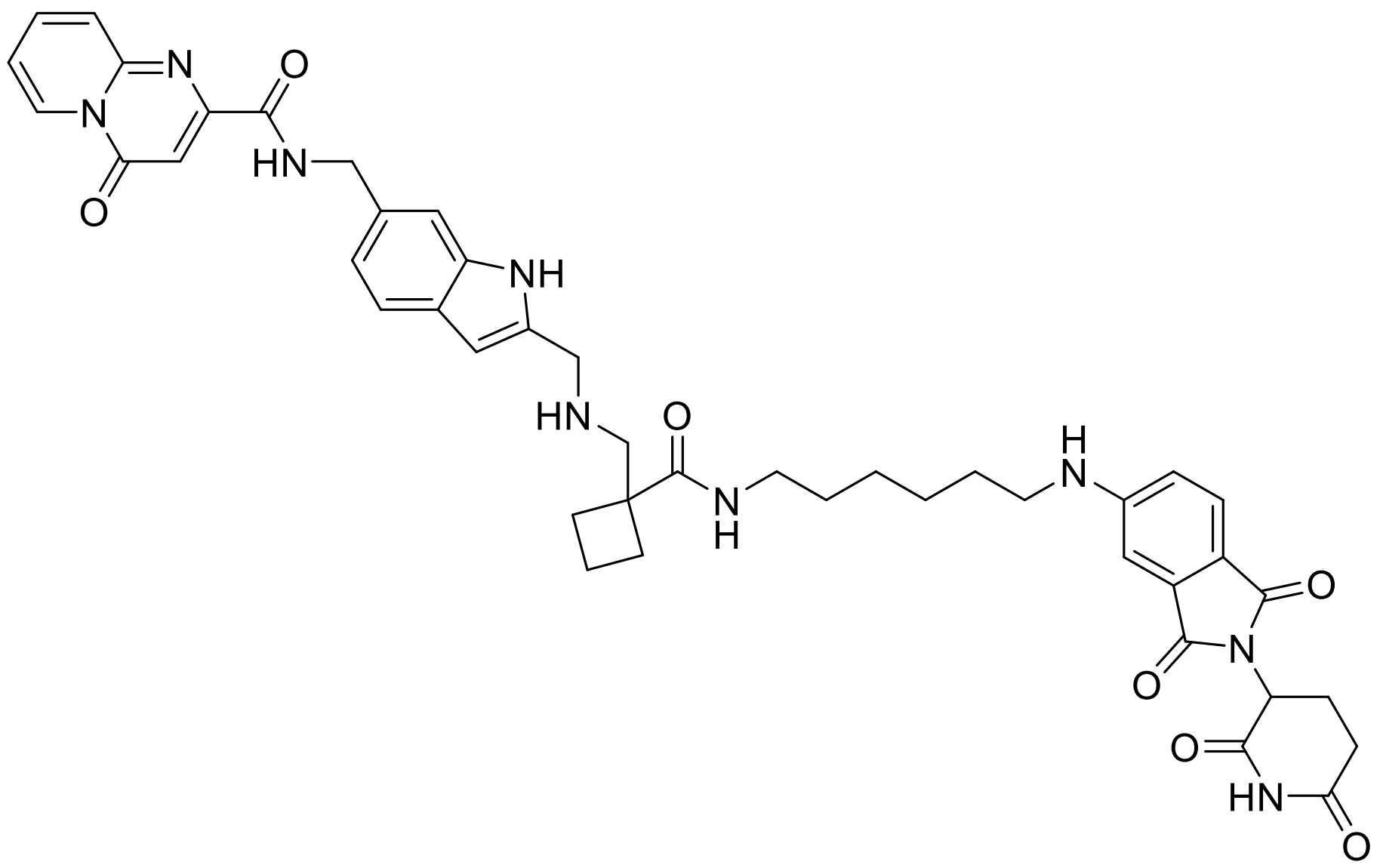

STC-17 (10.0 mg, 21.8 μmol) and **L05** (8.1 mg, 21.8 μmol) were coupled according to **General Procedure C** and purified according to the **General Purification Procedure for METTL3 PROTACs** to afford the title compound as a yellow solid (4.48 mg, 5.5 μmol, 25.3 % yield).

HRMS: Calculated for $[C_{44}H_{48}N_9O_7]^+$: 814.3671; found, 814.3683.

^{1}H NMR (500 MHz, Methanol-d_4) δ 8.96 – 8.93 (m, 1H), 7.86 (ddd, *J* = 8.5, 6.7, 1.6 Hz, 1H), 7.69 (dt, *J* = 8.9, 1.1 Hz, 1H), 7.42 (d, *J* = 8.2 Hz, 1H), 7.38 (d, *J* = 8.4 Hz, 1H), 7.35 – 7.32 (m, 1H), 7.28 (td, *J* = 6.9, 1.4 Hz, 1H), 7.01 (dd, *J* = 8.2, 1.5 Hz, 1H), 6.98 (s, 1H), 6.77 (d, *J* = 2.2 Hz, 1H), 6.64 (dd, *J* = 8.4, 2.1 Hz, 1H), 6.54 (d, *J* = 0.9 Hz, 1H), 4.92 (dd, *J* = 12.7, 5.5 Hz, 1H), 4.59 (s, 2H), 4.29 (s, 2H), 3.25 (s, 2H), 3.05 (t, *J* = 7.1 Hz, 2H), 3.01 (t, *J* = 7.0 Hz, 2H), 2.78 – 2.69 (m, 1H), 2.66 – 2.53 (m, 2H), 2.31 – 2.25 (m, 2H), 2.02 – 1.95 (m, 1H), 1.95 – 1.89 (m, 3H), 1.85 (ddd, *J* = 9.6, 7.1, 4.2 Hz, 1H), 1.47 (p, *J* = 7.2 Hz, 2H), 1.36 (dd, *J* = 15.3, 8.0 Hz, 2H), 1.25 (dd, *J* = 10.8, 5.1 Hz, 2H), 1.21 – 1.15 (m, 2H).

N-((2-((((1-((8-((2-(2,6-Dioxopiperidin-3-yl) -1,3-dioxoisoindolin-5-yl) amino) octyl) carbamoyl) cyclobutyl) methyl) amino) methyl) -1H-indol-6-yl) methyl) -4-oxo-4H-pyrido [1,2-a] pyrimidine-2-carboxamide (**4**)

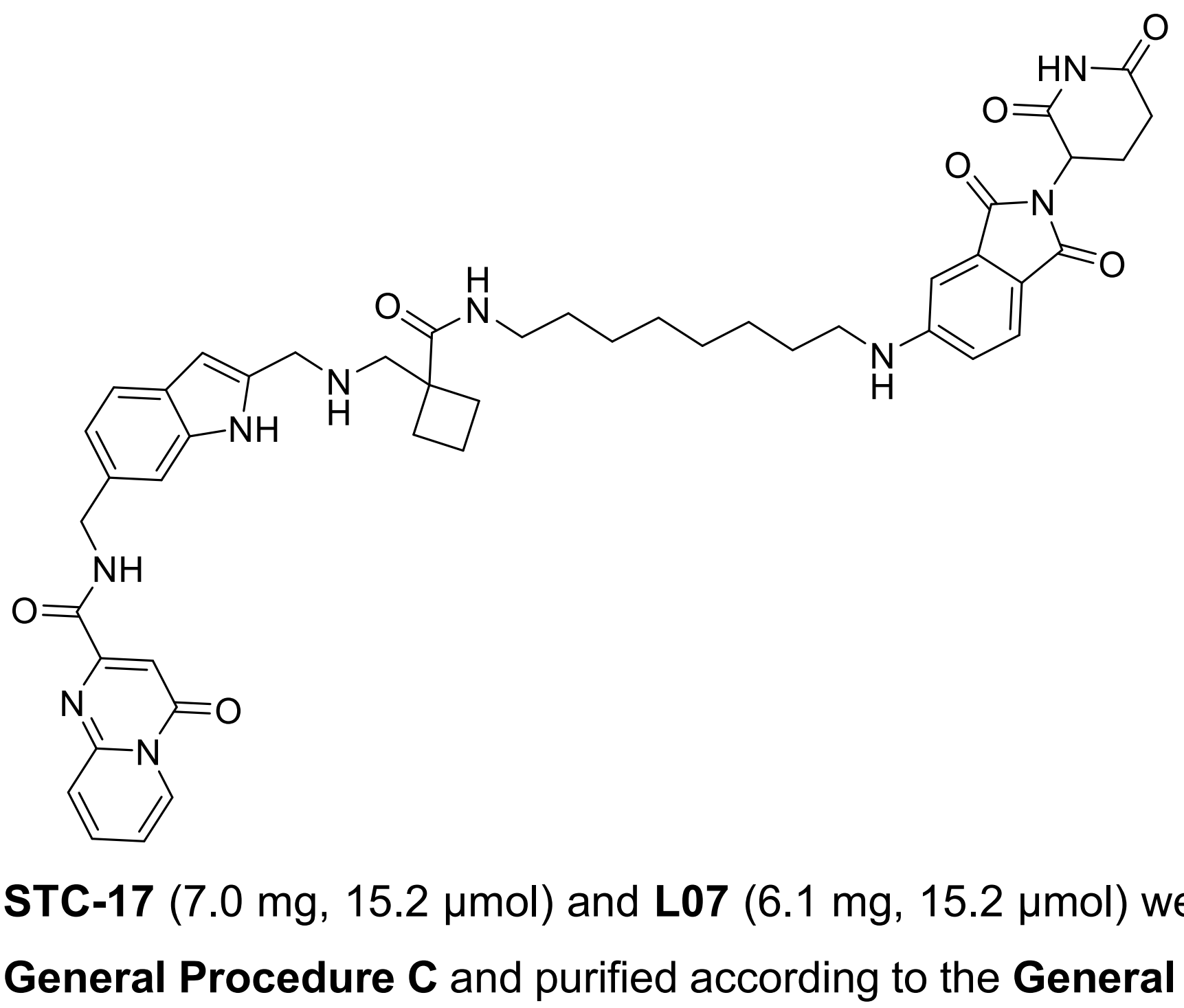

STC-17 (7.0 mg, 15.2 μmol) and **L07** (6.1 mg, 15.2 μmol) were coupled according to **General Procedure C** and purified according to the **General Purification Procedure for METTL3 PROTACs** to afford the title compound as a yellow solid (1.93 mg, 2.3 μmol, 15.0 % yield).

HRMS: Calculated for [$C_{46}H_{52}N_9O_7$] $^+$: 842.3984; found, 842.3992.

^{1}H NMR (700 MHz, Acetone-*d*6) δ 9.04 (dt, *J* = 6.4, 1.1 Hz, 1H), 8.00 (ddd, *J* = 8.5, 6.7, 1.6 Hz, 1H), 7.70 (dt, *J* = 8.9, 1.2 Hz, 1H), 7.53 (dd, *J* = 15.8, 8.3 Hz, 2H), 7.46 (s, 1H), 7.40 (td, *J* = 6.9, 1.3 Hz, 1H), 7.13 (dd, *J* = 8.1, 1.4 Hz, 1H), 7.03 (s, 1H), 6.98 (d, *J* = 2.2 Hz, 1H), 6.90 (dd, *J* = 8.3, 2.2 Hz, 1H), 6.67 – 6.62 (m, 1H), 5.04 (dd, *J* = 12.7, 5.5 Hz, 1H), 4.72 (d, *J* = 5.2 Hz, 2H), 4.62 (s, 2H), 3.48 (s, 2H), 3.25 (t, *J* = 7.1 Hz, 2H), 3.19 (t, *J* = 7.1 Hz, 2H), 2.98 – 2.90 (m, 1H), 2.80 – 2.71 (m, 2H), 2.42 (ddd, *J* = 13.0, 9.6, 7.6 Hz, 2H), 2.23 – 2.12 (m, 3H), 2.09 (s, 3H), 2.02 – 1.95 (m, 1H), 1.90 (tdd, *J* = 9.6, 6.5, 4.6 Hz, 1H), 1.65 (p, *J* = 7.3 Hz, 2H), 1.48 (dd, *J* = 10.2, 3.4 Hz, 2H), 1.41 (ddd, *J* = 11.9, 8.4, 6.1 Hz, 2H), 1.37 – 1.23 (m, 7H). **^{13}C NMR** (176 MHz, Acetone) δ 172.70, 172.63, 170.38, 170.31, 168.68, 168.13, 163.59, 163.53, 159.00, 155.44, 154.96, 154.95, 151.93, 138.70, 138.46, 135.75, 134.56, 128.32, 127.97, 127.93, 127.85, 127.25, 125.75, 121.51, 121.05, 118.24, 117.48, 111.86, 111.85, 105.92, 102.08, 52.82, 49.96, 49.94, 45.57, 45.54, 44.40, 44.28, 43.78, 40.22, 40.09, 32.01, 27.62, 27.48, 23.50, 15.83.

N-((2-((((1-((9-((2-(2,6-Dioxopiperidin-3-yl) -1,3-dioxoisoindolin-5-yl) amino) nonyl) carbamoyl) cyclobutyl) methyl) amino) methyl)-1H-indol-6-yl) methyl)-4-oxo-4H-pyrido [1,2-a] pyrimidine-2-carboxamide (**5**)

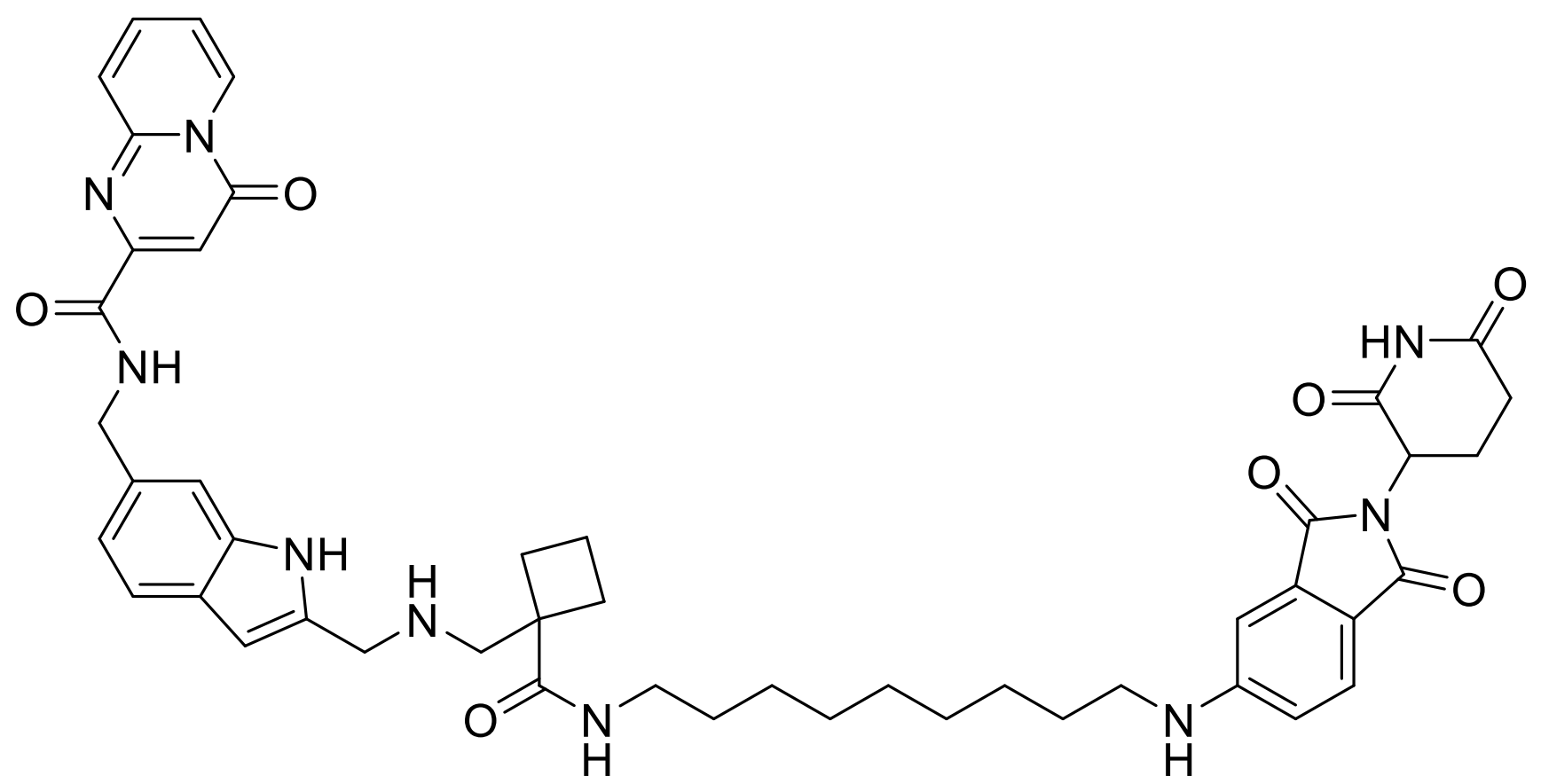

STC-17 (8.0 mg, 17.4 µmol) and **L08** (7.2 mg, 17.4 µmol) were coupled according to **General Procedure C** and purified according to the **General Purification Procedure for METTL3 PROTACs** to afford the title compound as a yellow solid (2.91 mg, 3.4 µmol, 19.5 % yield).

HRMS: Calculated for [$C_{47}H_{54}N_9O_7$] $^+$: 856.4141; found, 856.4157.

^{1}H NMR (500 MHz, Methanol-d_4) δ 9.08 (dt, *J* = 7.2, 1.2 Hz, 1H), 8.00 (ddd, *J* = 8.6, 6.7, 1.6 Hz, 1H), 7.82 (dd, *J* = 8.9, 1.1 Hz, 1H), 7.55 (d, *J* = 8.2 Hz, 1H), 7.52 (d, *J* = 8.4 Hz, 1H), 7.47 (s, 1H), 7.41 (td, *J* = 7.0, 1.4 Hz, 1H), 7.14 (dd, *J* = 8.3, 1.5 Hz, 1H), 7.11 (s, 1H), 6.93 (d, *J* = 2.1 Hz, 1H), 6.79 (dd, *J* = 8.4, 2.2 Hz, 1H), 6.67 (s, 1H), 5.03 (dd, *J* = 12.7, 5.4 Hz, 1H), 4.72 (s, 2H), 4.41 (s, 2H), 3.37 (s, 2H), 3.17 (dt, *J* = 12.4, 7.1 Hz, 4H), 2.84 (ddd, *J* = 17.4, 14.0, 5.3 Hz, 1H), 2.77 – 2.65 (m, 2H), 2.41 (ddt, *J* = 14.0, 6.3, 3.0 Hz, 2H), 2.09 (ddt, *J* = 12.7, 5.4, 2.9 Hz, 1H), 2.06 – 2.01 (m, 3H), 2.00 – 1.93 (m, 1H), 1.62 (q, *J* = 7.3 Hz, 2H), 1.49 – 1.39 (m, 4H), 1.37 – 1.25 (m, 9H).

N-((2-((((1-((3-(((S)-1-((2S,4R) - 4-Hydroxy-2-(((S)-1-(4-(4-methylthiazol-5-yl) phenyl) ethyl) carbamoyl) pyrrolidin-1-yl) -3,3-dimethyl-1-oxobutan-2-yl) amino) -3-oxopropyl) carbamoyl) cyclobutyl) methyl) amino) methyl) -1H-indol-6-yl) methyl) -4-oxo-4H-pyrido [1,2-a] pyrimidine-2-carboxamide (**6**)

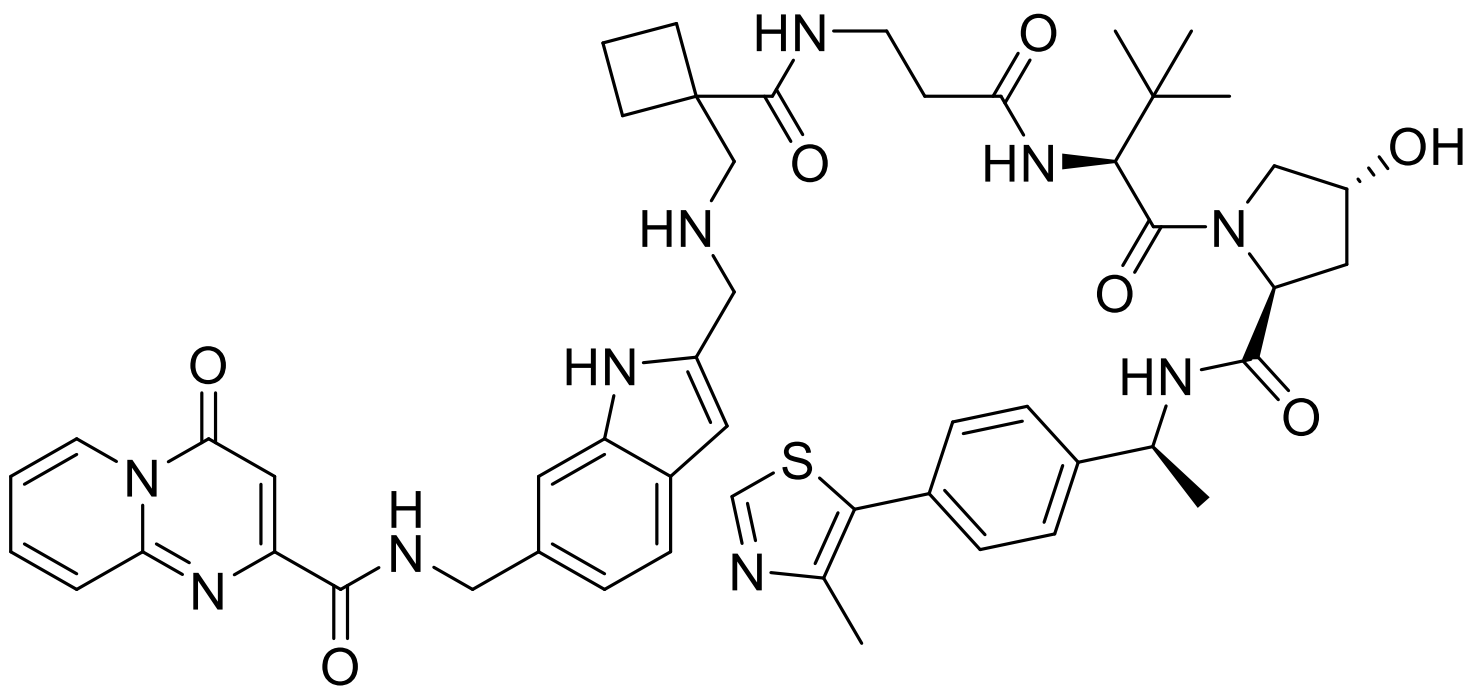

STC-17 (7.0 mg, 15.2 µmol) and **L09** (7.8 mg, 15.2 µmol) were coupled according to **General Procedure C** and purified according to the **General Purification Procedure for METTL3 PROTACs** to afford the title compound as a yellow solid (3.44 mg, 3.6 µmol, 23.6 % yield).

HRMS: Calculated for $[C_{51}H_{61}N_{10}O_7S]^+$: 957.4440; found, 957.4460.

^{1}H NMR (700 MHz, Acetone-d_6) δ 11.07 (s, 1H), 9.04 (dt, *J* = 7.3, 1.2 Hz, 1H), 8.91 – 8.86 (m, 1H), 8.45 (s, 1H), 7.97 (ddd, *J* = 8.6, 6.7, 1.6 Hz, 1H), 7.83 (d, *J* = 36.7 Hz, 1H), 7.74 – 7.68 (m, 1H), 7.56 (d, *J* = 8.2 Hz, 1H), 7.50 (s, 1H), 7.43 (s, 4H), 7.38 (td, *J* = 6.9, 1.3 Hz, 1H), 7.15 (dd, *J* = 8.2, 1.5 Hz, 1H), 7.04 (s, 1H), 4.99 (t, *J* = 7.1 Hz, 1H), 4.73 (dd, *J* = 6.3, 3.2 Hz, 2H), 4.68 – 4.55 (m, 3H), 4.44 (d, *J* = 8.8 Hz, 1H), 4.27 (s, 1H), 3.77 (s, 1H), 3.49 (d, *J* = 38.4 Hz, 3H), 3.32 (d, *J* = 38.6 Hz, 2H), 2.67 – 2.63 (m, 1H), 2.46 (s, 3H), 2.43 – 2.37 (m, 1H), 2.36 – 2.27 (m, 1H), 2.14 (p, *J* = 2.2 Hz, 1H), 1.96 (p, *J* = 2.2 Hz, 2H), 1.87 (q, *J* = 9.3 Hz, 1H), 1.76 (dq, *J* = 11.1, 4.8 Hz, 1H), 1.40 (d, *J* = 7.0 Hz, 2H), 0.98 (s, 9H).

N-((2-((((1-((4-((3-(((S)-1-((2S,4R)-4-Hydroxy-2-(((S)-1-(4-(4-methylthiazol-5-yl) phenyl) ethyl) carbamoyl) pyrrolidin-1-yl) -3,3-dimethyl-1-oxobutan-2-yl) amino) -3-oxopropyl) amino) -4-oxobutyl) carbamoyl) cyclobutyl) methyl) amino) methyl)-1H-indol-6-yl) methyl)-4-oxo-4H-pyrido[1,2-a]pyrimidine-2-carboxamide (**7**)

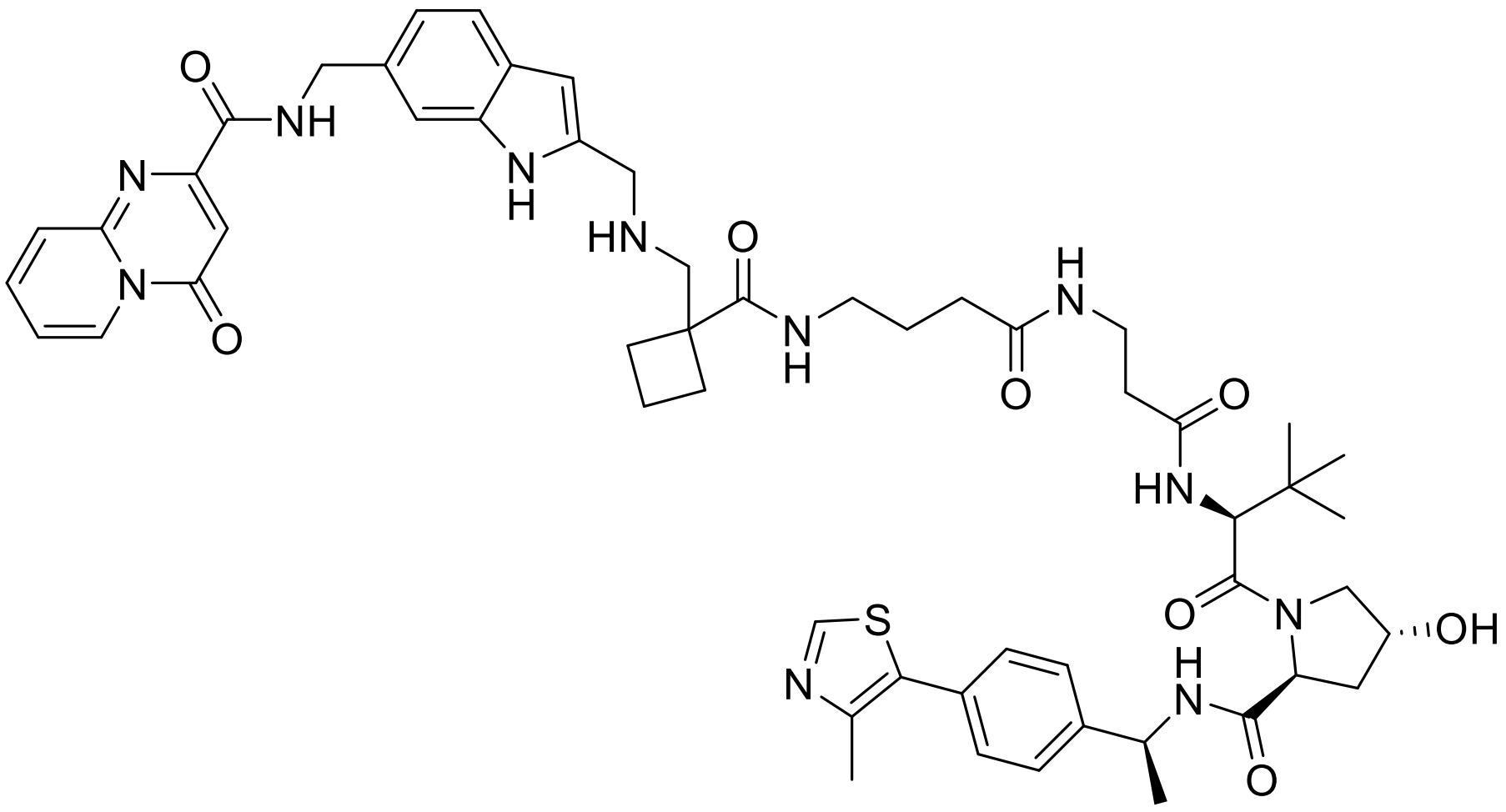

STC-17 (5.0 mg, 10.9 µmol) and **L10** (5.8 mg, 10.9 µmol) were coupled according to **General Procedure C** and purified according to the **General Purification Procedure for METTL3 PROTACs** to afford the title compound as a yellow solid (1.36 mg, 1.4 µmol, 12.9 % yield).

HRMS: Calculated for $[C_{55}H_{68}N_{11}O_8S]^+$: 1042.4968; found, 1042.4974.

^{1}H NMR (500 MHz, Chloroform-*d*) δ 10.96 (s, 1H), 9.25 (t, *J* = 6.3 Hz, 1H), 8.95 (dt, *J* = 6.8, 1.2 Hz, 1H), 8.68 (s, 2H), 8.03 – 7.95 (m, 2H), 7.90 (d, *J* = 8.7 Hz, 1H), 7.71 (dt, *J* = 8.9, 1.2 Hz, 1H), 7.44 (d, *J* = 8.1 Hz, 1H), 7.39 (td, *J* = 6.9, 1.4 Hz, 1H), 7.35 (s, 1H), 7.00 (dd, *J* = 8.2, 1.6 Hz, 1H), 6.84 (s, 1H), 6.53 (s, 1H), 4.52 (d, *J* = 6.4 Hz, 2H), 4.23 (s, 2H), 4.10 (d, *J* = 8.6 Hz, 1H), 3.54 (s, 2H), 3.16 – 3.12 (m, 2H), 2.99 (q, *J* = 6.7 Hz, 2H), 2.22 (q, *J* = 9.8 Hz, 2H), 2.18 – 2.04 (m, 2H), 1.91 – 1.84 (m, 2H), 1.80 (q, *J* = 9.4, 8.2 Hz, 1H), 1.72 (dd, *J* = 10.0, 5.1 Hz, 1H), 1.39 (d, *J* = 4.4 Hz, 2H), 1.33 (d, *J* = 7.2 Hz, 2H), 1.19 – 1.10 (m, 12H), 1.04 (d, *J* = 6.7 Hz, 1H), 0.98 (d, *J* = 6.8 Hz, 1H), 0.85 (s, 6H).

N-((2-((((1-((10-(((S)-1-((2S,4R)-4-Hydroxy-2-(((S)-1-(4-(4-methylthiazol-5-yl) phenyl) ethyl) carbamoyl) pyrrolidin-1-yl)-3,3-dimethyl-1-oxobutan-2-yl) amino)-10-oxodecyl) carbamoyl) cyclobutyl) methyl) amino) methyl) -1H-indol-6-yl)methyl)-4-oxo-4H-pyrido[1,2-a]pyrimidine-2-carboxamide (**8**)

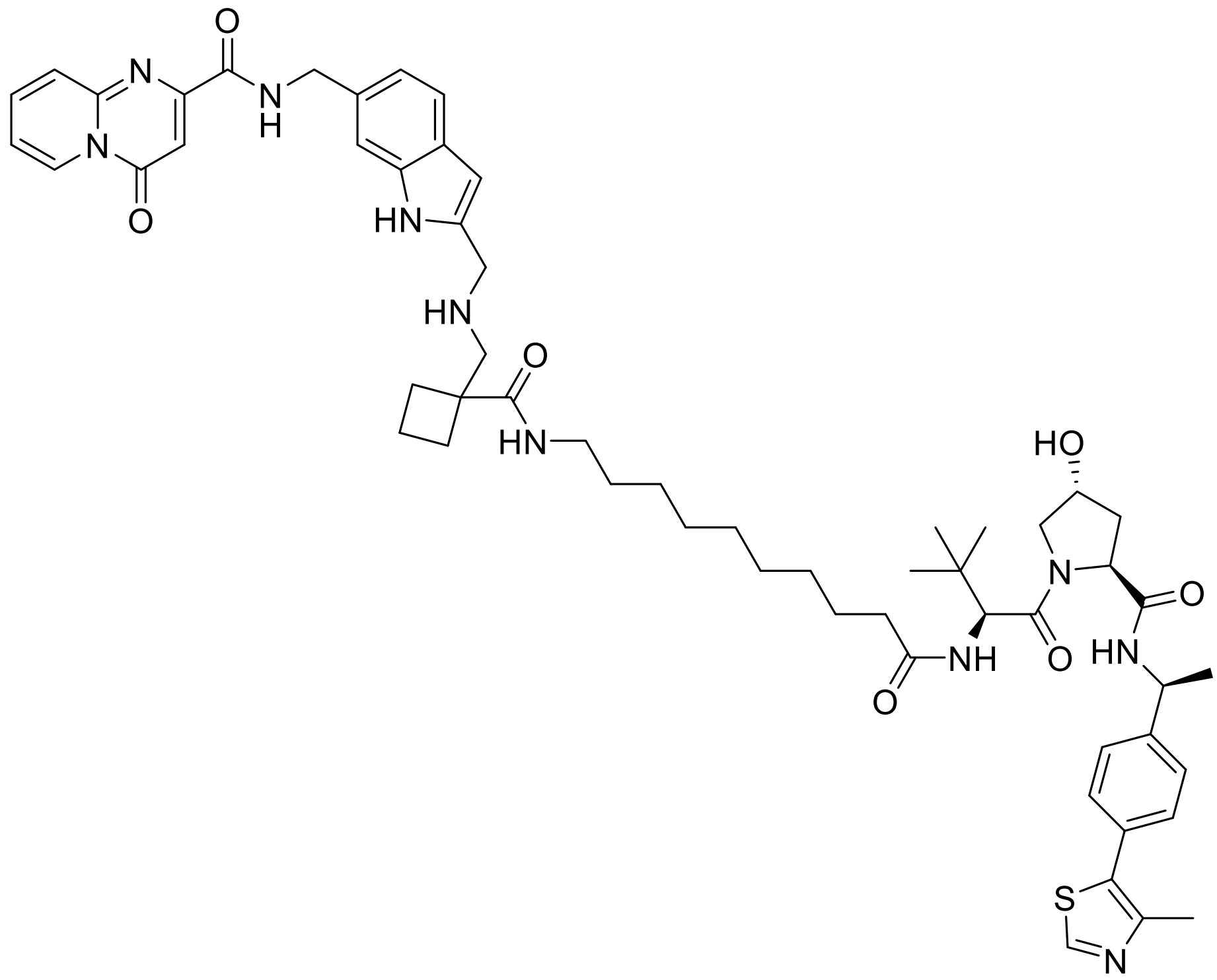

STC-17 (7.0 mg, 15.2 µmol) and **L12** (9.4 mg, 15.2 µmol) were coupled according to **General Procedure C** and purified according to the **General Purification Procedure for METTL3 PROTACs** to afford the title compound as a yellow solid (3.83 mg, 3.6 µmol, 23.8 % yield).

HRMS: Calculated for $[C_{58}H_{75}N_{10}O_7S]^+$: 1055.5535; found, 1055.5545.

1H NMR (700 MHz, Acetone-*d*6) δ 11.39 (s, 1H), 9.54 (s, 1H), 9.04 (dd, *J* = 7.1, 0.8 Hz, 1H), 8.92 (t, *J* = 6.3 Hz, 1H), 8.85 (s, 1H), 8.00 (ddd, *J* = 8.9, 6.7, 1.6 Hz, 1H), 7.86 (t, *J* = 6.2 Hz, 1H), 7.79 – 7.75 (m, 1H), 7.69 (d, *J* = 8.9 Hz, 1H), 7.52 (d, *J* = 8.2 Hz, 1H), 7.49 (s, 1H), 7.45 (d, *J* = 8.1 Hz, 2H), 7.43 – 7.40 (m, 1H), 7.40 – 7.37 (m, 2H), 7.15 (d, *J* = 8.9 Hz, 1H), 7.13 (dd, *J* = 8.2, 1.4 Hz, 1H), 7.04 (s, 1H), 6.65 (s, 1H), 4.73 – 4.70 (m, 2H), 4.68 – 4.63 (m, 2H), 4.62 (s, 2H), 4.58 – 4.53 (m, 2H), 4.33 (dd, *J* = 15.4, 5.2 Hz, 1H), 3.92 (dd, *J* = 10.8, 1.8 Hz, 1H), 3.77 (dd, *J* = 10.7, 4.1 Hz, 1H), 3.53 – 3.46 (m, 2H), 3.22 (dt, *J* = 14.4, 6.5 Hz, 1H), 3.19 – 3.12 (m, 1H), 2.47 – 2.39 (m, 5H), 2.27 – 2.22 (m, 1H), 2.22 – 2.15 (m, 4H), 2.15 – 2.10 (m, 1H), 2.09 (s, 3H), 1.92 – 1.87 (m, 1H), 1.60 – 1.50 (m, 2H), 1.47 (q, *J* = 7.1 Hz, 2H), 1.24 (d, *J* = 7.8 Hz, 10H), 1.01 (s, 9H). **^{13}C NMR** (176 MHz, Acetone) δ 176.59, 176.51, 173.57, 173.49, 172.65, 172.57, 171.79, 171.77, 163.65, 163.59, 160.33, 160.11, 159.89, 159.68, 159.02, 154.94, 154.93, 151.91, 151.38, 149.02, 140.46, 140.45, 138.72, 138.55, 138.38, 134.56, 132.36, 131.28, 129.87, 129.18, 129.17, 128.71, 128.70, 128.33, 128.01, 127.98, 127.26, 121.52, 121.51, 121.10, 121.09, 117.67, 117.51, 116.03, 112.02,

111.96, 105.93, 105.88, 102.12, 70.67, 60.15, 60.11, 58.01, 57.92, 57.46, 52.87, 45.54, 45.51, 44.44, 44.31, 43.13, 43.01, 40.08, 39.95, 38.50, 36.28, 36.23, 36.13, 27.16, 26.95, 26.37, 26.36, 16.25, 15.85.

4-(((6-(4-(4-((4,4-Dimethylpiperidin-1-yl) methyl) phenyl) -2-oxo-1,4,9-triazaspiro [5.5] undecan-9-yl) pyrimidin-4-yl) amino) methyl) -N-(3-((2-(2,6-dioxopiperidin-3-yl) -1,3-dioxoisoindolin-5-yl) amino) propyl) benzamide (**9**)

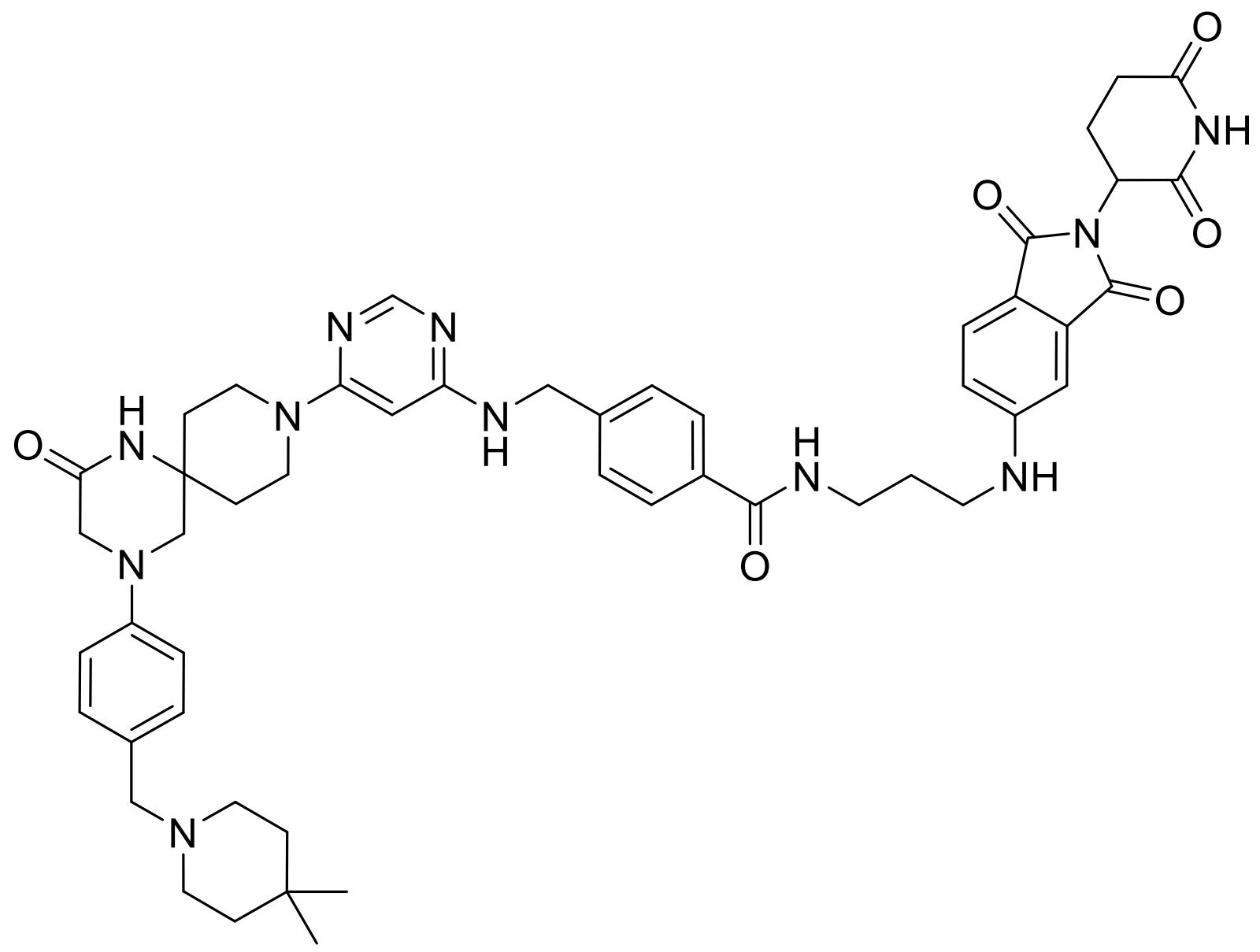

MD-28 (10.0 mg, 16.7 µmol) and **L02** (6.7 mg, 18.4 µmol) were coupled according to **General Procedure C** and purified according to the **General Purification Procedure for METTL3 PROTACs** to afford the title compound as a yellow solid (5.36 mg, 5.9 µmol, 35.2 % yield).

HRMS: Calculated for $[C_{50}H_{60}N_{11}O_6]^+$: 910.4723; found, 910.4737.

^{1}H NMR (700 MHz, DMSO-d_6) δ 10.99 (s, 1H), 8.97 (s, 1H), 8.43 (t, *J* = 5.6 Hz, 1H), 8.36 (s, 1H), 8.20 (s, 1H), 7.77 (d, *J* = 8.2 Hz, 2H), 7.50 (d, *J* = 8.3 Hz, 1H), 7.35 (d, *J* = 7.9 Hz, 2H), 7.24 (d, *J* = 8.6 Hz, 2H), 7.08 (s, 1H), 6.95 (d, *J* = 8.7 Hz, 2H), 6.89 (d, *J* = 2.3 Hz, 1H), 6.79 (dd, *J* = 8.5, 2.1 Hz, 1H), 5.84 (s, 1H), 4.96 (dd, *J* = 12.9, 5.4 Hz, 1H), 4.52 (d, *J* = 5.7 Hz, 2H), 4.13 (s, 2H), 3.70 (s, 2H), 3.41 (s, 2H), 3.30 (t, *J* = 6.4 Hz, 2H), 3.16 (t, *J* = 7.1 Hz, 2H), 3.08 (d, *J* = 12.3 Hz, 2H), 2.96 – 2.90 (m, 2H), 2.80 (ddd, *J* = 17.2, 13.9, 5.5 Hz, 1H), 2.52 – 2.48 (m, 1H), 2.45 (t, *J* = 1.9 Hz, 1H), 1.92 (ddq, *J* = 10.5, 5.6, 2.7 Hz, 1H), 1.77 (p, *J* = 6.9 Hz, 2H), 1.69 (q, *J* = 7.2, 5.3 Hz, 2H), 1.63 – 1.57 (m, 2H), 1.47 – 1.42 (m, 4H), 0.92 (s, 3H), 0.88 (s, 3H).

^{13}C NMR (176 MHz, MeOD) δ 174.67, 171.82, 170.46, 169.81, 169.61, 169.27, 163.30, 163.10, 162.90, 162.74, 162.54, 162.34, 156.01, 151.67, 150.21, 141.78, 135.99, 135.20, 133.59, 128.89, 128.50, 126.24, 120.45, 118.85, 118.31, 117.19, 115.72, 61.00, 53.87, 53.81, 51.82, 50.31, 49.80, 45.91, 41.73, 38.71, 36.59, 35.46, 32.20, 31.49, 29.57, 28.81, 23.85, 23.46.

4-(((6-(4-(4-((4,4-Dimethylpiperidin-1-yl) methyl) phenyl) -2-oxo-1,4,9-triazaspiro [5.5] undecan-9-yl) pyrimidin-4-yl) amino) methyl) -N-(4-((2-(2,6-dioxopiperidin-3-yl) -1,3-dioxoisoindolin-5-yl) amino) butyl) benzamide (**10**)

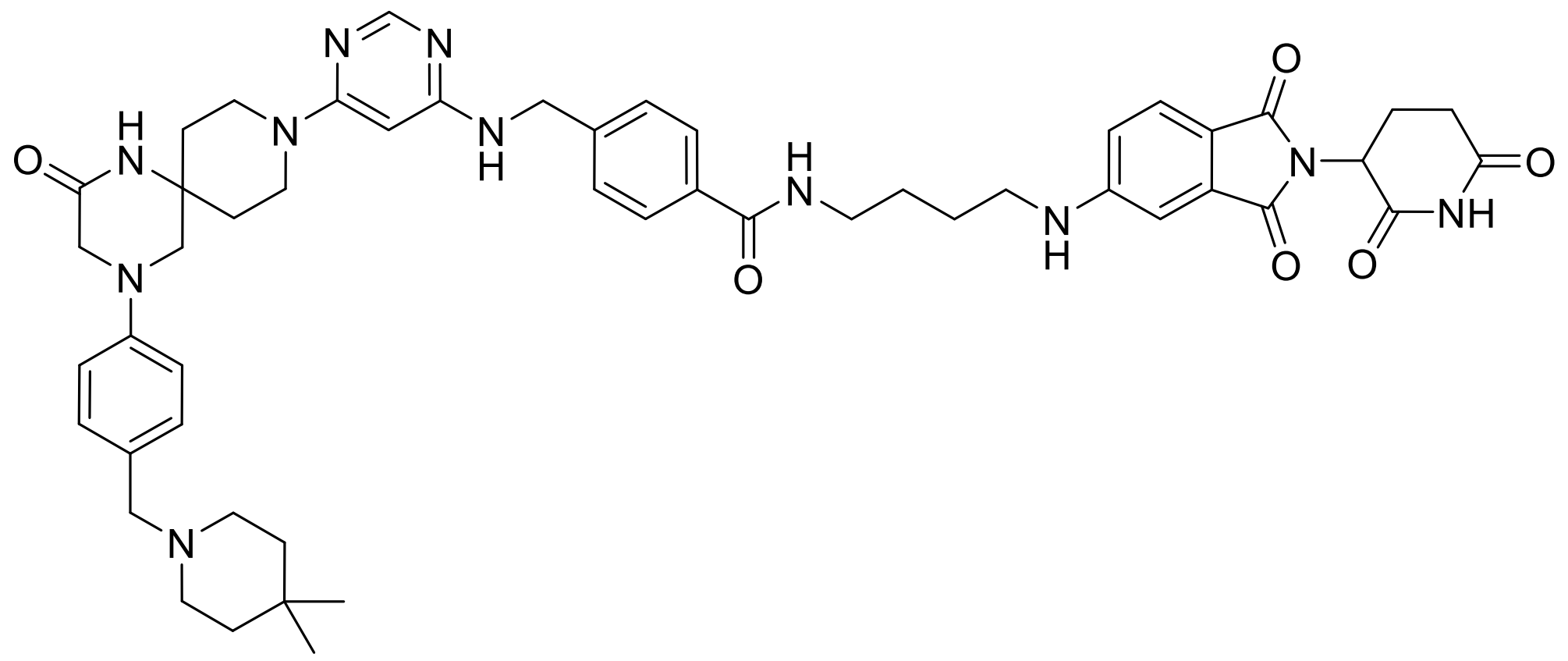

MD-28 (7.5 mg, 12.5 μmol) and **L03** (4.3 mg, 12.5 μmol) were coupled according to **General Procedure C** and purified according to the **General Purification Procedure for METTL3 PROTACs** to afford the title compound as a yellow solid (5.37 mg, 5.8 μmol, 46.3 % yield).

HRMS: Calculated for $[C_{51}H_{62}N_{11}O_6]^+$: 924.4879; found, 924.4883.

^{1}H NMR (700 MHz, DMSO-d_6) δ 11.06 (s, 1H), 9.05 (s, 1H), 8.47 (t, *J* = 5.7 Hz, 1H), 8.44 (s, 1H), 8.29 (s, 1H), 7.83 (d, *J* = 8.3 Hz, 2H), 7.55 (d, *J* = 8.3 Hz, 1H), 7.42 (d, *J* = 7.9 Hz, 2H), 7.32 (d, *J* = 8.7 Hz, 2H), 7.13 (s, 1H), 7.02 (d, *J* = 8.9 Hz, 2H), 6.96 (d, *J* = 2.1 Hz, 1H), 6.85 (dd, *J* = 8.4, 2.2 Hz, 1H), 5.92 (s, 1H), 5.03 (dd, *J* = 12.9, 5.5 Hz, 1H), 4.59 (d, *J* = 6.0 Hz, 2H), 4.20 (d, *J* = 5.4 Hz, 2H), 3.77 (s, 2H), 3.31 (d, *J* = 5.9 Hz, 2H), 3.20 (s, 2H), 3.15 (d, *J* = 12.3 Hz, 2H), 3.00 (qd, *J* = 12.1, 11.1, 7.4 Hz, 2H), 2.88 (ddd, *J* = 17.1, 13.9, 5.4 Hz, 1H), 2.61 – 2.56 (m, 1H), 2.53 – 2.52 (m, 1H), 1.99 (ddd, *J* = 10.5, 5.4, 2.8 Hz, 1H), 1.78 (td, *J* = 8.2, 7.4, 3.8 Hz, 2H), 1.68 (td, *J* = 8.3, 3.8 Hz, 2H), 1.65 – 1.59 (m, 4H), 1.56 – 1.49 (m, 4H), 1.00 (s, 3H), 0.95 (s, 3H).

^{13}C NMR (176 MHz, MeOD) δ 174.66, 171.87, 170.46, 169.70, 169.64, 169.33, 163.29, 163.10, 162.90, 162.54, 162.34, 162.14, 156.08, 151.67, 150.08, 141.64, 135.96, 135.32, 133.60, 128.83, 128.47, 126.21, 120.44, 118.65, 118.04, 116.99,

115.72, 61.00, 53.88, 53.80, 51.82, 50.30, 49.80, 45.90, 43.66, 40.54, 36.59, 35.46, 32.21, 31.49, 28.81, 27.86, 26.96, 23.86, 23.46.

4-(((6-(4-(4-((4,4-Dimethylpiperidin-1-yl) methyl) phenyl) -2-oxo-1,4,9-triazaspiro [5.5] undecan-9-yl) pyrimidin-4-yl) amino) methyl) -N-(5-((2-(2,6-dioxopiperidin-3-yl) -1,3-dioxoisoindolin-5-yl) amino) pentyl) benzamide (**11**)

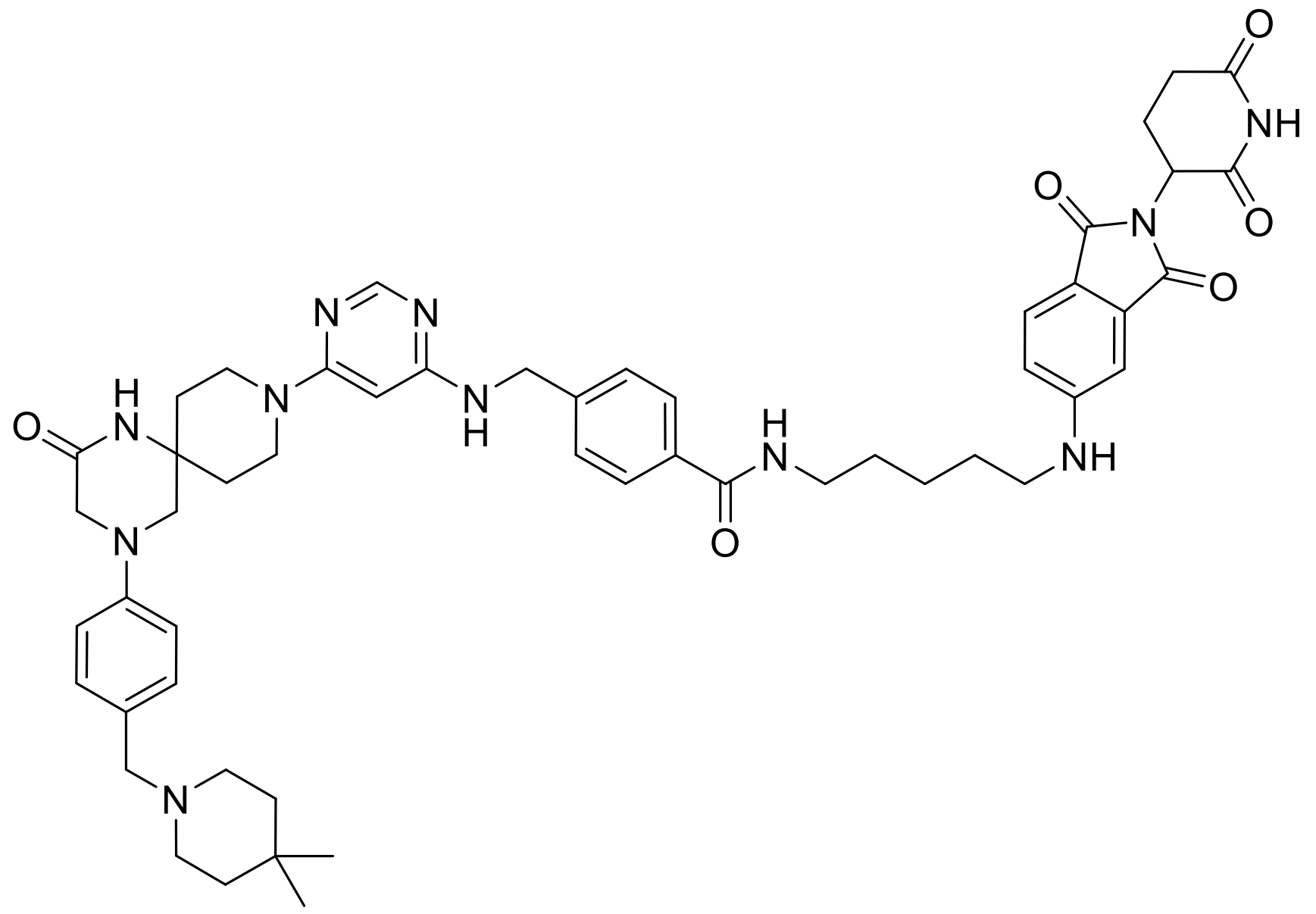

MD-28 (10.0 mg, 16.7 μmol) and **L04** (6.6 mg, 16.7 μmol) were coupled according to **General Procedure C** and purified according to the **General Purification Procedure for METTL3 PROTACs** to afford the title compound as a yellow solid (3.23 mg, 3.4 μmol, 20.6 % yield).

HRMS: Calculated for $[C_{52}H_{64}N_{11}O_6]^+$: 938.5036; found, 938.5030.

^{1}H NMR (700 MHz, DMSO-d_6) δ 10.99 (s, 1H), 8.93 (s, 1H), 8.36 (s, 2H), 8.23 – 8.16 (m, 1H), 7.76 (d, *J* = 8.0 Hz, 2H), 7.48 (d, *J* = 8.4 Hz, 1H), 7.34 (d, *J* = 7.9 Hz, 2H), 7.24 (d, *J* = 8.4 Hz, 2H), 7.04 (s, 1H), 6.95 (d, *J* = 8.5 Hz, 2H), 6.87 (d, *J* = 2.1 Hz, 1H), 6.77 (dd, *J* = 8.4, 2.1 Hz, 1H), 5.83 (s, 1H), 4.95 (dd, *J* = 12.9, 5.5 Hz, 1H), 4.51 (d, *J* = 5.7 Hz, 2H), 4.12 (d, *J* = 5.3 Hz, 2H), 3.70 (s, 2H), 3.41 (s, 2H), 3.20 (q, *J* = 6.6 Hz, 2H), 3.08 (d, *J* = 12.5 Hz, 4H), 2.93 (td, *J* = 15.0, 12.9, 8.6 Hz, 2H), 2.80 (ddd, *J* = 17.1, 13.9, 5.5 Hz, 1H), 2.52 – 2.49 (m, 1H), 2.46 – 2.45 (m, 1H), 1.92 (dtd, *J* = 13.0, 5.5, 2.5 Hz, 1H), 1.69 (q, *J* = 6.6, 3.8 Hz, 2H), 1.63 – 1.58 (m, 2H), 1.57 – 1.51 (m, 2H), 1.49 (q, *J* = 7.3 Hz, 2H), 1.44 (t, *J* = 4.8 Hz, 4H), 1.34 (qd, *J* = 9.5, 8.8, 6.0 Hz, 2H), 0.92 (s, 3H), 0.88 (s, 3H).

4-(((6-(4-(4-((4,4-Dimethylpiperidin-1-yl) methyl) phenyl) -2-oxo-1,4,9-triazaspiro [5.5] undecan-9-yl) pyrimidin-4-yl) amino) methyl) -N-(7-((2-(2,6-dioxopiperidin-3-yl) -1,3-dioxoisoindolin-5-yl) amino) heptyl) benzamide (**12**)

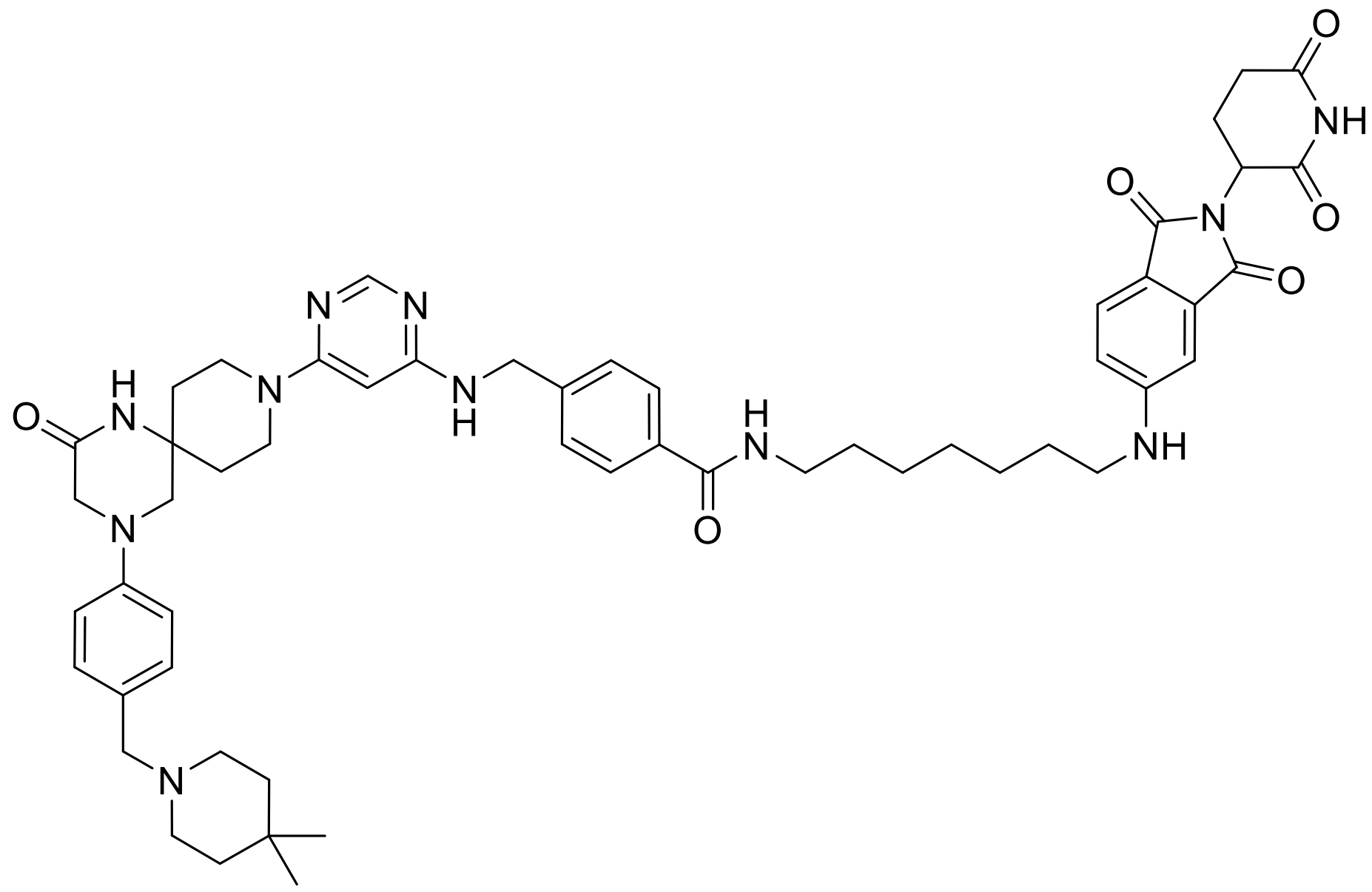

MD-28 (10.0 mg, 15.8 μmol) and **L06** (6.1 mg, 15.8 μmol) were coupled according to **General Procedure C** and purified according to the **General Purification Procedure for METTL3 PROTACs** to afford the title compound as a yellow solid (5.12 mg, 5.3 μmol, 33.5 % yield).

HRMS: Calculated for $[C_{54}H_{68}N_{11}O_6]^+$: 966.5349; found, 966.5363.

^{1}H NMR (700 MHz, Methanol-d_4) δ 8.20 (s, 1H), 7.82 (d, *J* = 8.3 Hz, 2H), 7.52 (d, *J* = 8.4 Hz, 1H), 7.46 (d, *J* = 8.0 Hz, 2H), 7.38 (d, *J* = 8.7 Hz, 2H), 7.03 (d, *J* = 8.8 Hz, 2H), 6.95 (d, *J* = 2.1 Hz, 1H), 6.80 (dd, *J* = 8.4, 2.2 Hz, 1H), 5.83 (s, 1H), 5.03 (dd, *J* = 12.7, 5.5 Hz, 1H), 4.62 (s, 2H), 4.21 (s, 2H), 3.88 (s, 2H), 3.53 (s, 2H), 3.38 (t, *J* = 7.1 Hz, 2H), 3.28 (d, *J* = 2.7 Hz, 1H), 3.19 (t, *J* = 7.1 Hz, 2H), 3.09 (ddd, *J* = 12.8, 10.3, 6.2 Hz, 2H), 2.84 (ddd, *J* = 16.9, 13.7, 5.2 Hz, 1H), 2.76 – 2.67 (m, 2H), 2.66 (s, 2H), 2.11 – 2.06 (m, 1H), 2.04 (s, 1H), 2.03 – 1.99 (m, 1H), 1.93 (ddd, *J* = 11.7, 7.3, 3.9 Hz, 2H), 1.83 (td, *J* = 9.0, 8.6, 4.0 Hz, 2H), 1.64 (dq, *J* = 14.0, 5.1, 3.2 Hz, 10H), 1.49 – 1.40 (m, 7H), 1.36 – 1.27 (m, 4H), 1.07 (s, 3H), 1.03 (s, 3H).

^{13}C NMR (176 MHz, MeOD) δ 174.68, 171.85, 170.72, 170.46, 169.71, 169.61, 169.33, 163.30, 163.10, 162.90, 156.25, 151.69, 135.98, 135.35, 133.60, 133.36, 128.81, 128.45, 126.24, 120.41, 117.89, 115.71, 53.85, 51.82, 50.34, 50.30, 49.85, 49.81, 49.53, 45.87, 44.05, 42.06, 40.90, 36.60, 35.46, 32.21, 31.49, 30.33, 29.99, 29.68, 29.33, 28.81, 28.76, 27.95, 27.87, 23.86, 23.74, 23.45.

(2S,4R) -1-((S)-2-(4-(4-(((6-(4-(4-((4,4-Dimethylpiperidin-1-yl) methyl) phenyl) -2-oxo-1,4,9-triazaspiro [5.5] undecan-9-yl) pyrimidin-4-yl) amino) methyl) benzamido) butanamido) -3,3-dimethylbutanoyl) -4-hydroxy-N-((S)-1-(4-(4-methylthiazol-5-yl) phenyl) ethyl) pyrrolidine-2-carboxamide (**13**)

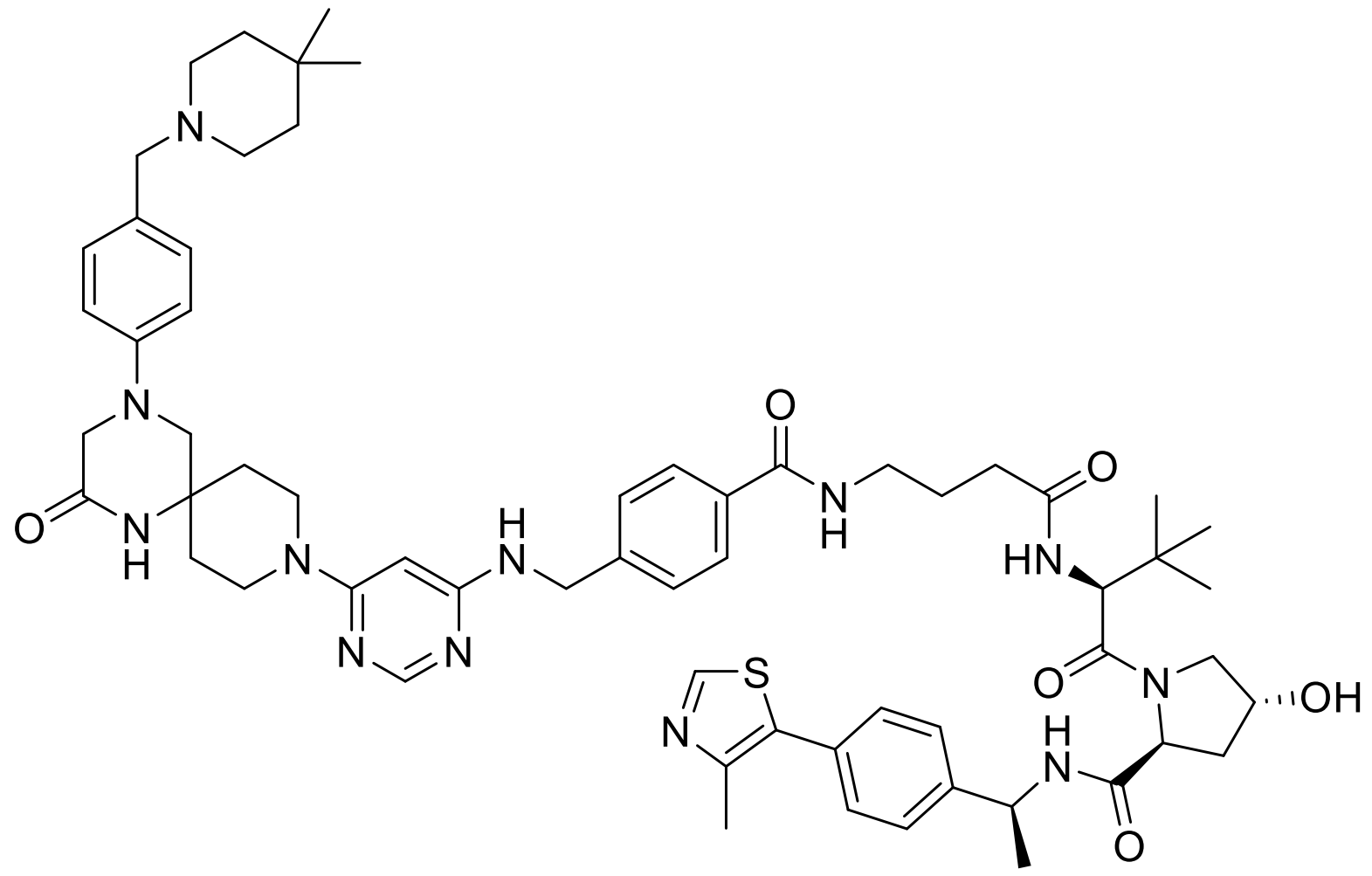

MD-28 (6.5 mg, 10.9 μmol) and **L10** (6.1 mg, 10.9 μmol) were coupled according to **General Procedure C** and purified according to the **General Purification Procedure for METTL3 PROTACs** to afford the title compound as a yellow solid (2.39 mg, 2.1 μmol, 19.8% yield).

HRMS: Calculated for $[C_{61}H_{81}N_{12}O_6S]^+$: 1109.6117; found, 1109.6113.

^{1}H NMR (700 MHz, DMSO-d_6) δ 8.99 (s, 1H), 8.43 (s, 1H), 8.41 (s, 1H), 8.36 (d, *J* = 7.8 Hz, 1H), 8.26 (s, 1H), 7.83 (d, *J* = 8.0 Hz, 2H), 7.77 (d, *J* = 9.3 Hz, 1H), 7.44 (d, *J* = 8.3 Hz, 2H), 7.42 (d, *J* = 8.0 Hz, 2H), 7.38 (d, *J* = 8.2 Hz, 2H), 7.32 (d, *J* = 8.6 Hz, 2H), 7.02 (d, *J* = 8.8 Hz, 2H), 5.90 (s, 1H), 4.92 (p, *J* = 7.1 Hz, 1H), 4.59 (d, *J* = 6.0 Hz, 2H), 4.52 (d, *J* = 9.3 Hz, 1H), 4.42 (t, *J* = 8.1 Hz, 1H), 4.30 – 4.27 (m, 1H), 4.20 (d, *J* = 5.3 Hz, 2H), 3.77 (s, 2H), 3.62 (dd, *J* = 10.5, 4.2 Hz, 1H), 3.59 (d, *J* = 2.1 Hz, 1H), 3.48 (s, 3H), 3.23 (q, *J* = 6.7 Hz, 3H), 3.16 (d, *J* = 12.2 Hz, 2H), 3.01 (dd, *J* = 13.4, 7.3 Hz, 2H), 2.53 – 2.52 (m, 2H), 2.46 (s, 3H), 2.25 (dt, *J* = 14.9, 7.7 Hz, 1H), 2.12 – 2.07 (m, 1H), 2.01 (tdd, *J* = 11.3, 8.5, 5.3 Hz, 2H), 1.82 – 1.74 (m, 3H), 1.71 – 1.65 (m, 2H), 1.51 (dq, *J* = 12.8, 7.1, 5.6 Hz, 8H), 1.38 (d, *J* = 7.0 Hz, 3H), 1.31 – 1.21 (m, 15H), 1.00 (s, 3H), 0.95 (s, 3H), 0.93 (s, 9H).

^{13}C NMR (176 MHz, MeOD) δ 175.42, 173.24, 172.36, 170.44, 169.59, 163.30, 163.10, 162.90, 152.93, 151.71, 149.05, 145.68, 135.29, 133.61, 133.37, 131.54, 130.52, 128.93, 128.47, 127.63, 120.45, 115.73, 71.00, 60.61, 59.32, 57.94, 53.88, 53.80, 51.84, 50.47, 50.34, 50.21, 50.14, 49.85, 49.81, 49.53, 45.90, 40.53, 38.83,

36.60, 36.40, 35.48, 34.98, 34.04, 31.50, 30.89, 28.82, 27.07, 27.03, 26.79, 23.45, 22.40, 15.77.

(2S,4R) -1-((S)-2-(6-(4-(((6-(4-(4-((4,4-Dimethylpiperidin-1-yl) methyl) phenyl) -2-oxo-1,4,9-triazaspiro [5.5] undecan-9-yl) pyrimidin-4-yl) amino) methyl) benzamido) hexanamido) -3,3-dimethylbutanoyl) -4-hydroxy-N-((S)-1-(4-(4-methylthiazol-5-yl) phenyl) ethyl) pyrrolidine-2-carboxamide (**14**)

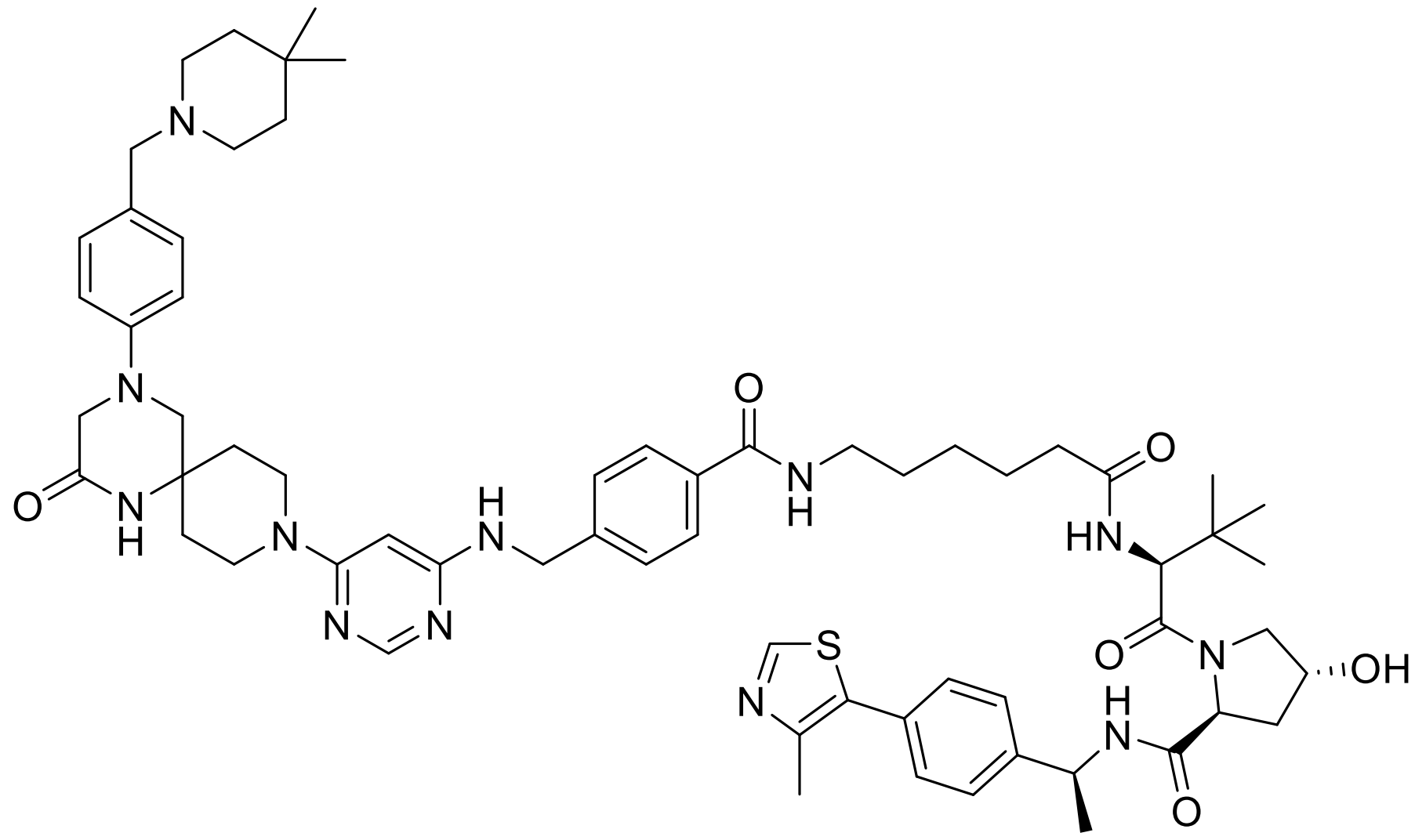

MD-28 (8.0 mg, 13.4 μmol) and **L11** (7.9 mg, 13.4 μmol) were coupled according to **General Procedure C** and purified according to the **General Purification Procedure for METTL3 PROTACs** to afford the title compound as a yellow solid (3.66 mg, 3.2 μmol, 24.0% yield).

HRMS: Calculated for $[C_{63}H_{85}N_{12}O_6S]^+$: 1137.6430; found, 1137.6448.

^{1}H NMR (700 MHz, DMSO-*d*$_6$) δ 8.99 (s, 1H), 8.44 (s, 1H), 8.41 (t, *J* = 5.7 Hz, 1H), 8.37 (d, *J* = 7.8 Hz, 1H), 8.26 (s, 1H), 7.83 (d, *J* = 7.8 Hz, 2H), 7.79 (d, *J* = 9.4 Hz, 1H), 7.43 (dd, *J* = 15.1, 8.2 Hz, 4H), 7.38 (d, *J* = 8.3 Hz, 2H), 7.32 (d, *J* = 8.6 Hz, 2H), 7.03 (d, *J* = 8.4 Hz, 2H), 5.90 (s, 1H), 4.92 (p, *J* = 7.1 Hz, 1H), 4.59 (d, *J* = 5.8 Hz, 2H), 4.52 (d, *J* = 9.4 Hz, 1H), 4.42 (t, *J* = 8.1 Hz, 1H), 4.28 (t, *J* = 2.4 Hz, 1H), 4.20 (d, *J* = 5.3 Hz, 2H), 3.77 (s, 3H), 3.61 (d, *J* = 1.3 Hz, 1H), 3.59 (s, 1H), 3.49 (s, 2H), 3.25 – 3.21 (m, 2H), 3.16 (d, *J* = 12.3 Hz, 2H), 3.01 (td, *J* = 14.9, 12.5, 8.9 Hz, 2H), 2.53 – 2.52 (m, 1H), 2.46 (s, 3H), 2.29 – 2.24 (m, 1H), 2.13 (ddd, *J* = 14.3, 8.3, 6.2 Hz, 1H), 2.01 (ddd, *J* = 13.7, 7.9, 2.7 Hz, 1H), 1.82 – 1.75 (m, 3H), 1.70 – 1.65 (m, 2H), 1.57 – 1.46 (m, 9H), 1.37 (d, *J* = 7.0 Hz, 3H), 1.31 – 1.25 (m, 2H), 1.00 (s, 3H), 0.95 (s, 3H), 0.93 (s, 9H).

NMR Spectra

^{1}H NMR Spectrum of **MD-28** (700 MHz, Methanol-d_4):

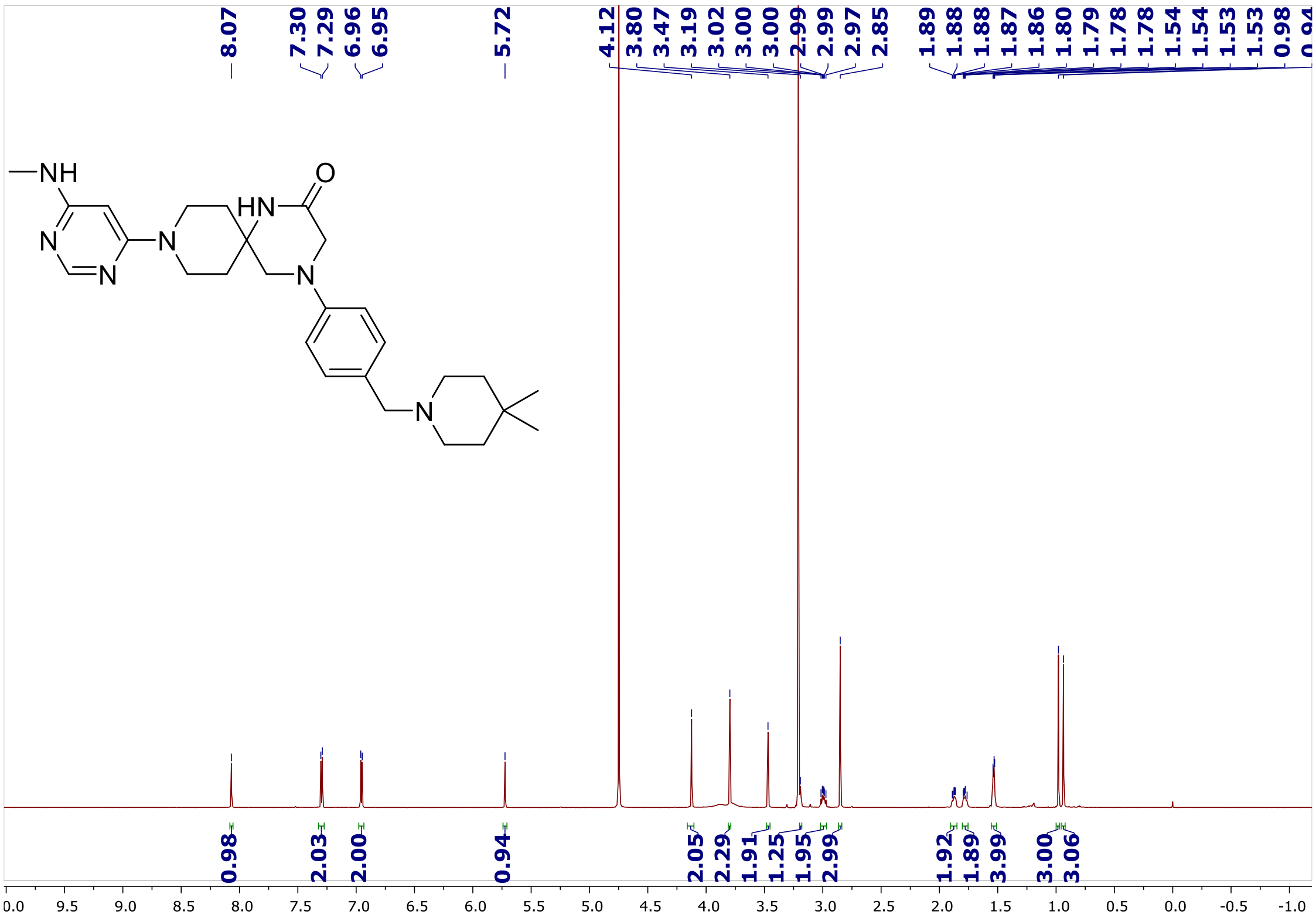

^{1}H NMR Spectrum of **1** (700 MHz, Acetone-d_6):

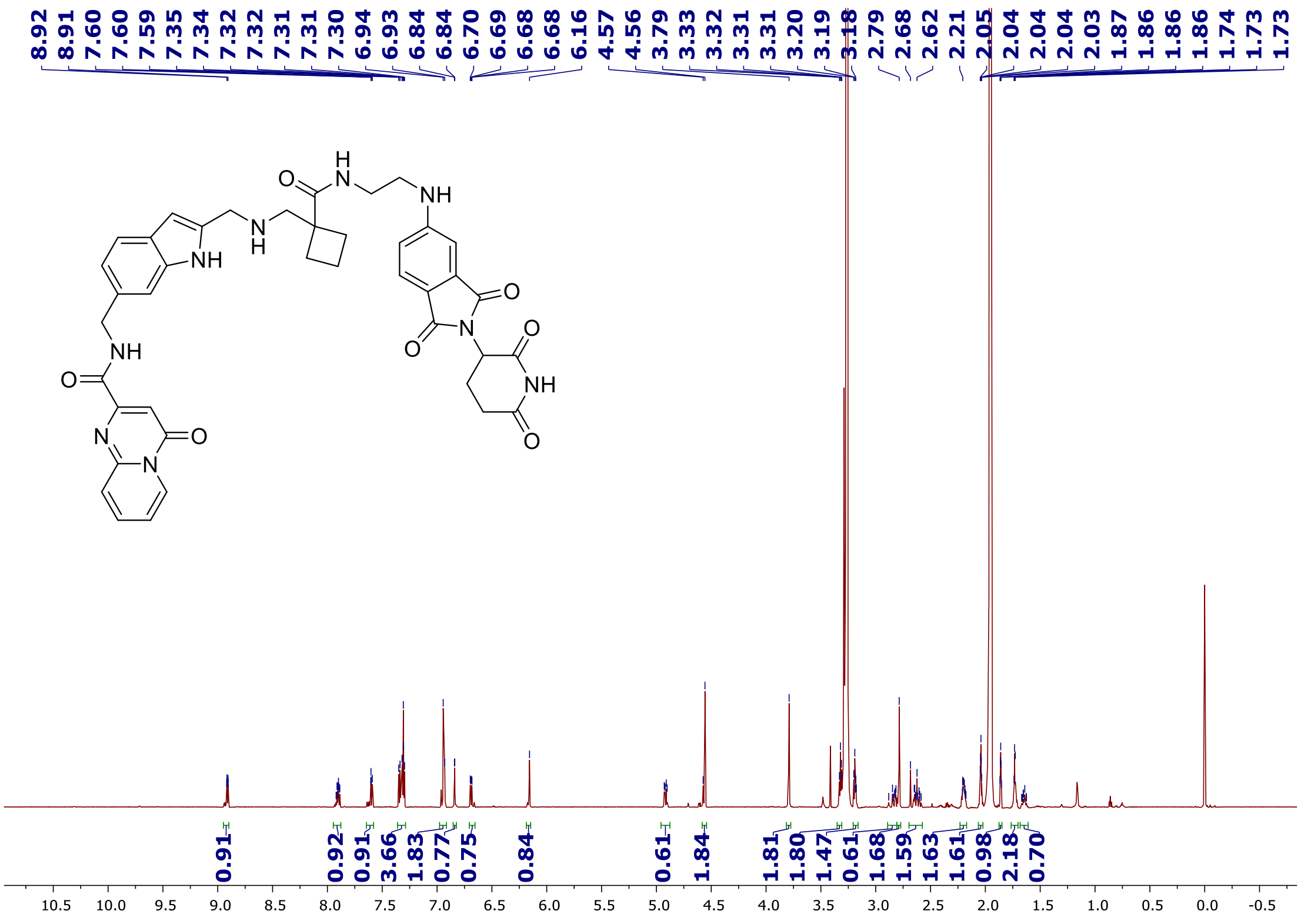

^{1}H NMR Spectrum of **2** (500 MHz, Methanol-d_4):

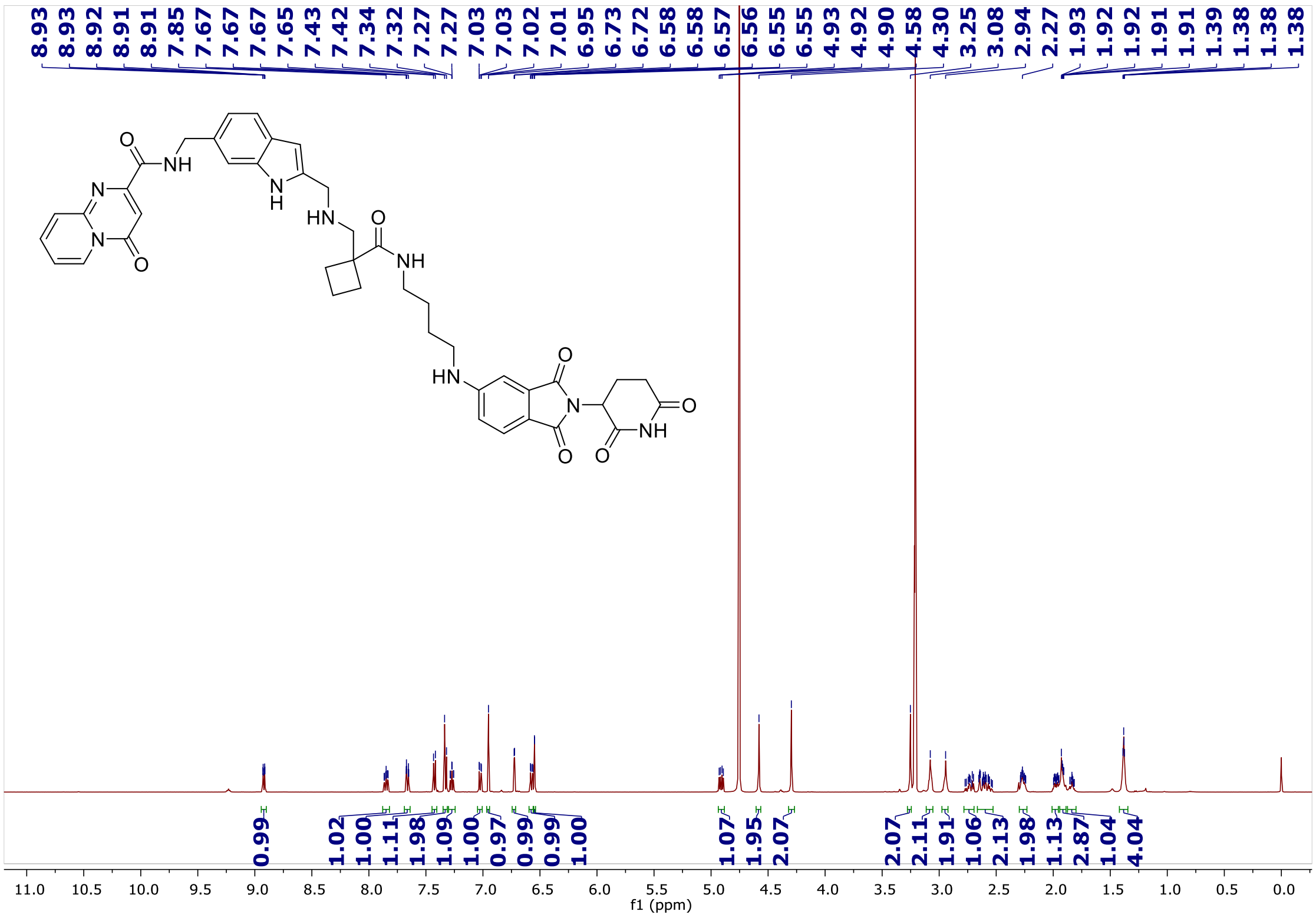

^{13}C NMR Spectrum of **2** (126 MHz, Methanol-d_4):

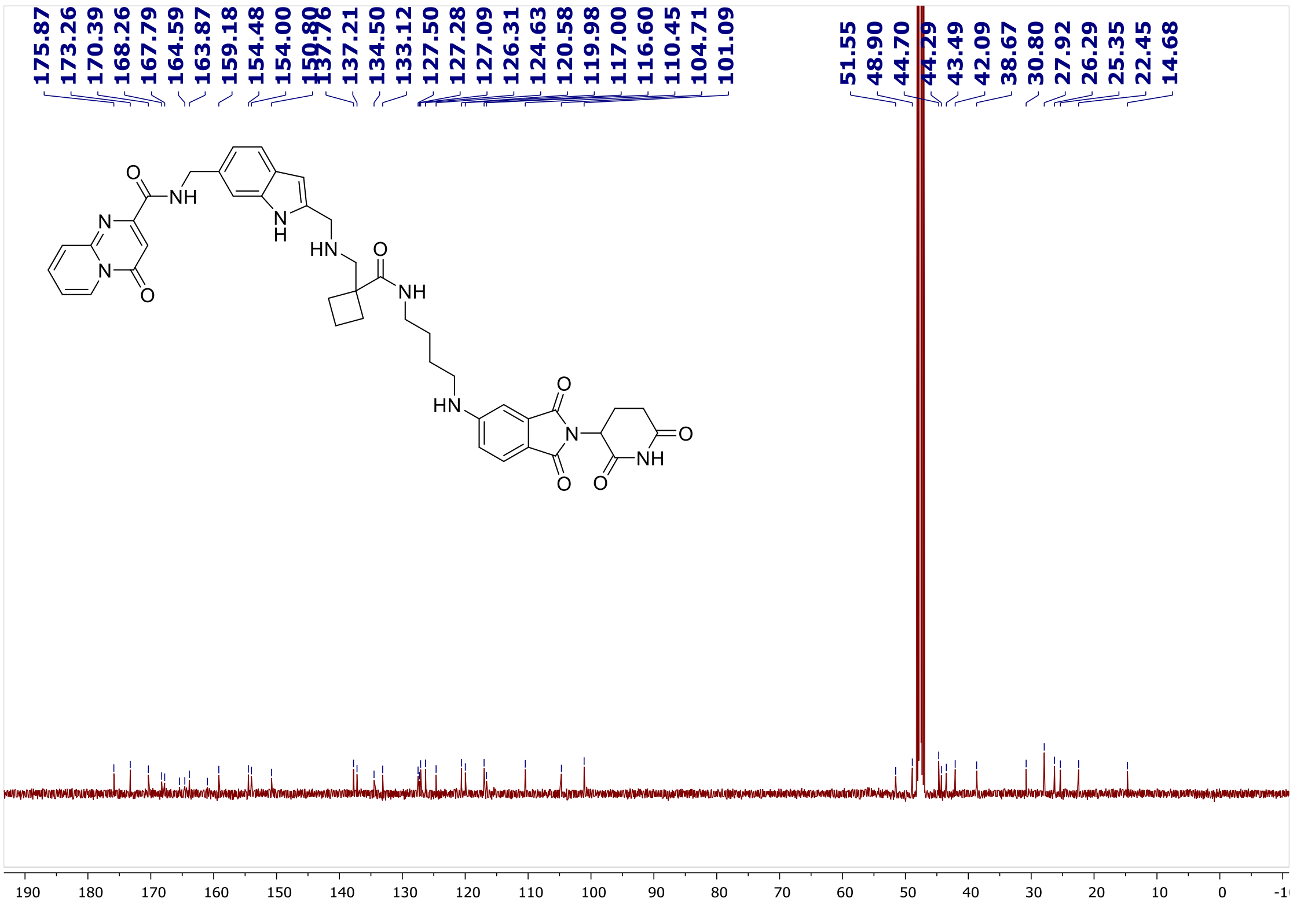

^{1}H NMR Spectrum of **3** (500 MHz, Methanol-d_4):

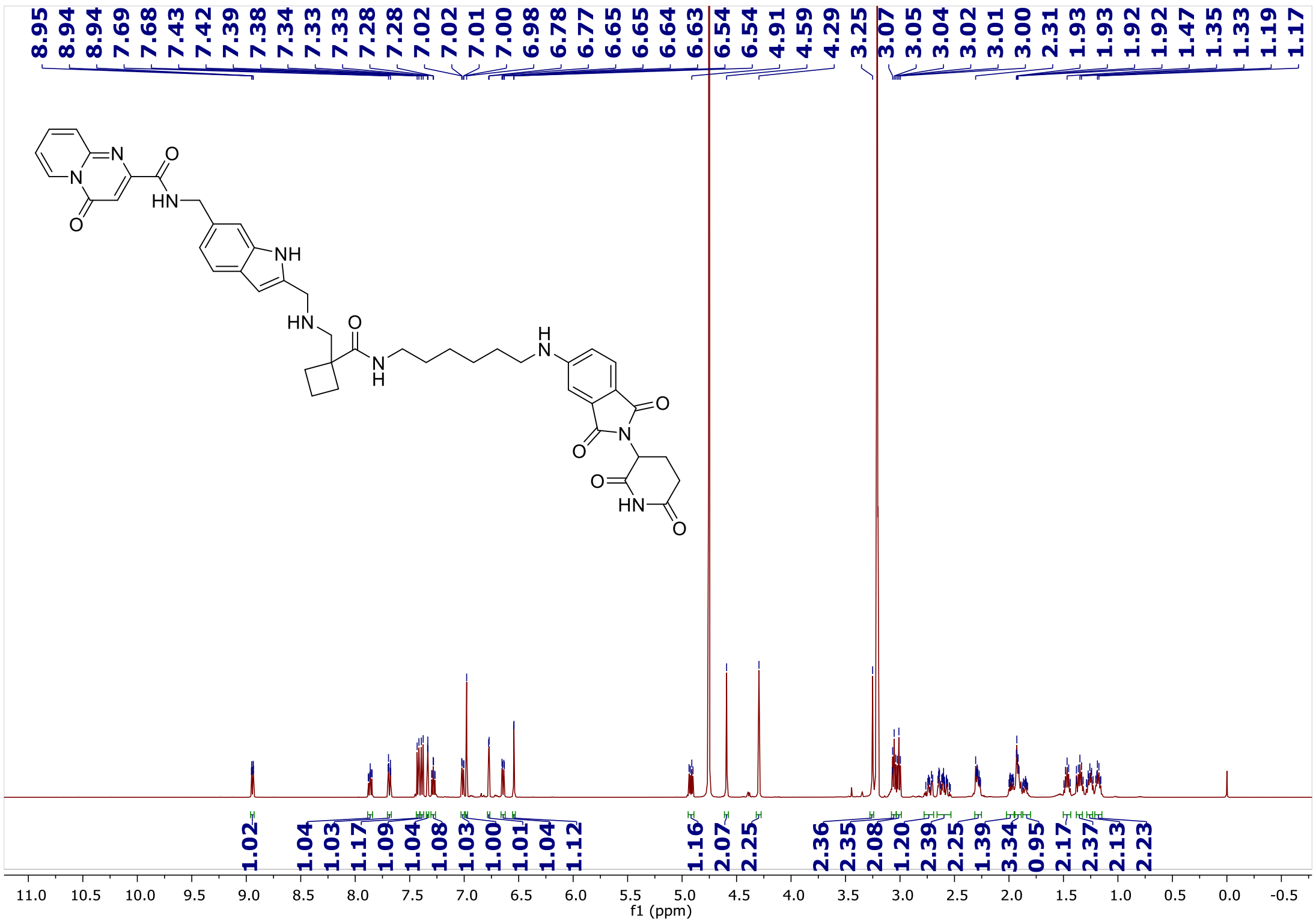

^{1}H NMR Spectrum of **4** (700 MHz, Acetone-d_6 and additional one drop of D_2O):

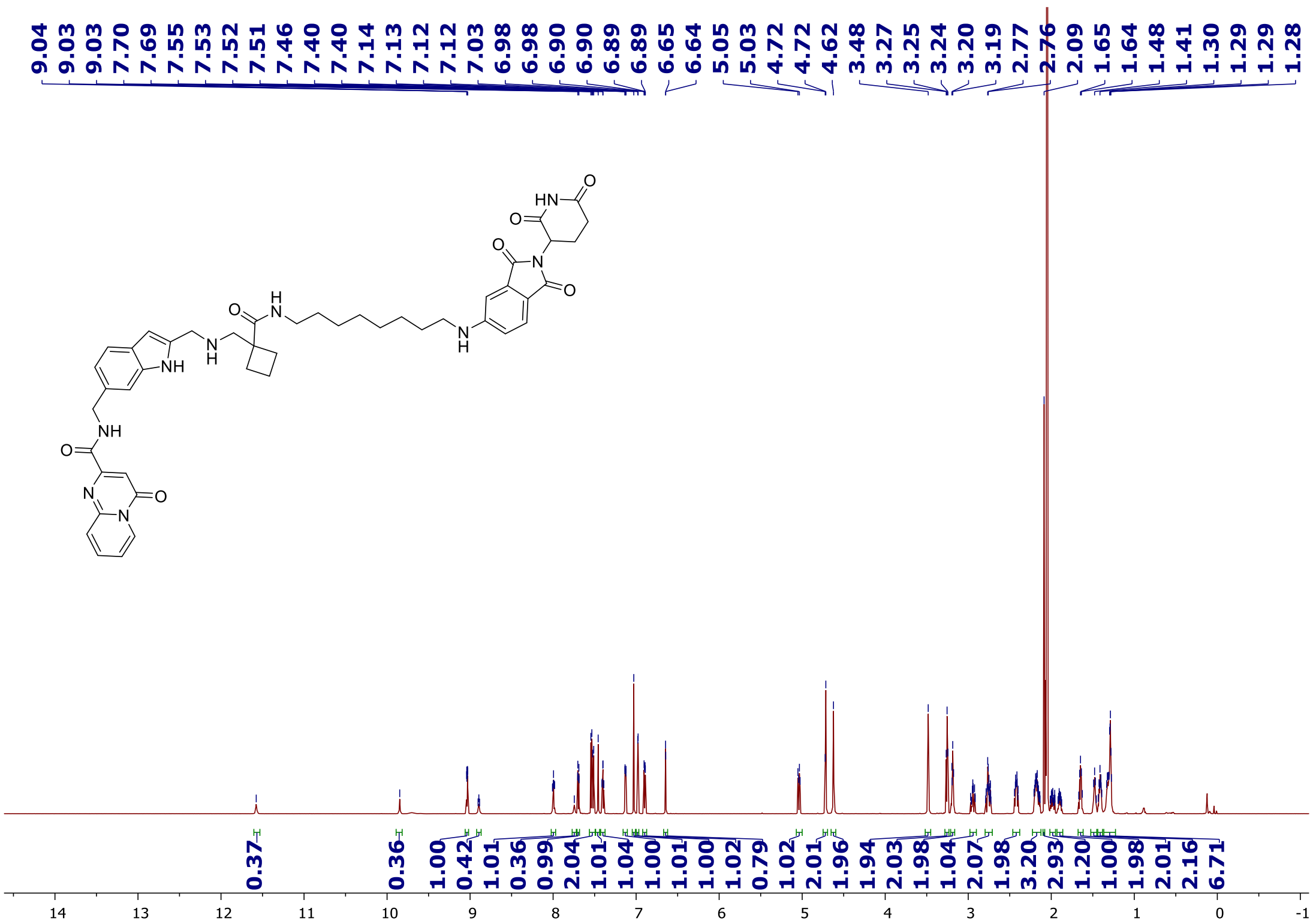

^{13}C NMR Spectrum of **4** (176 MHz, Acetone):

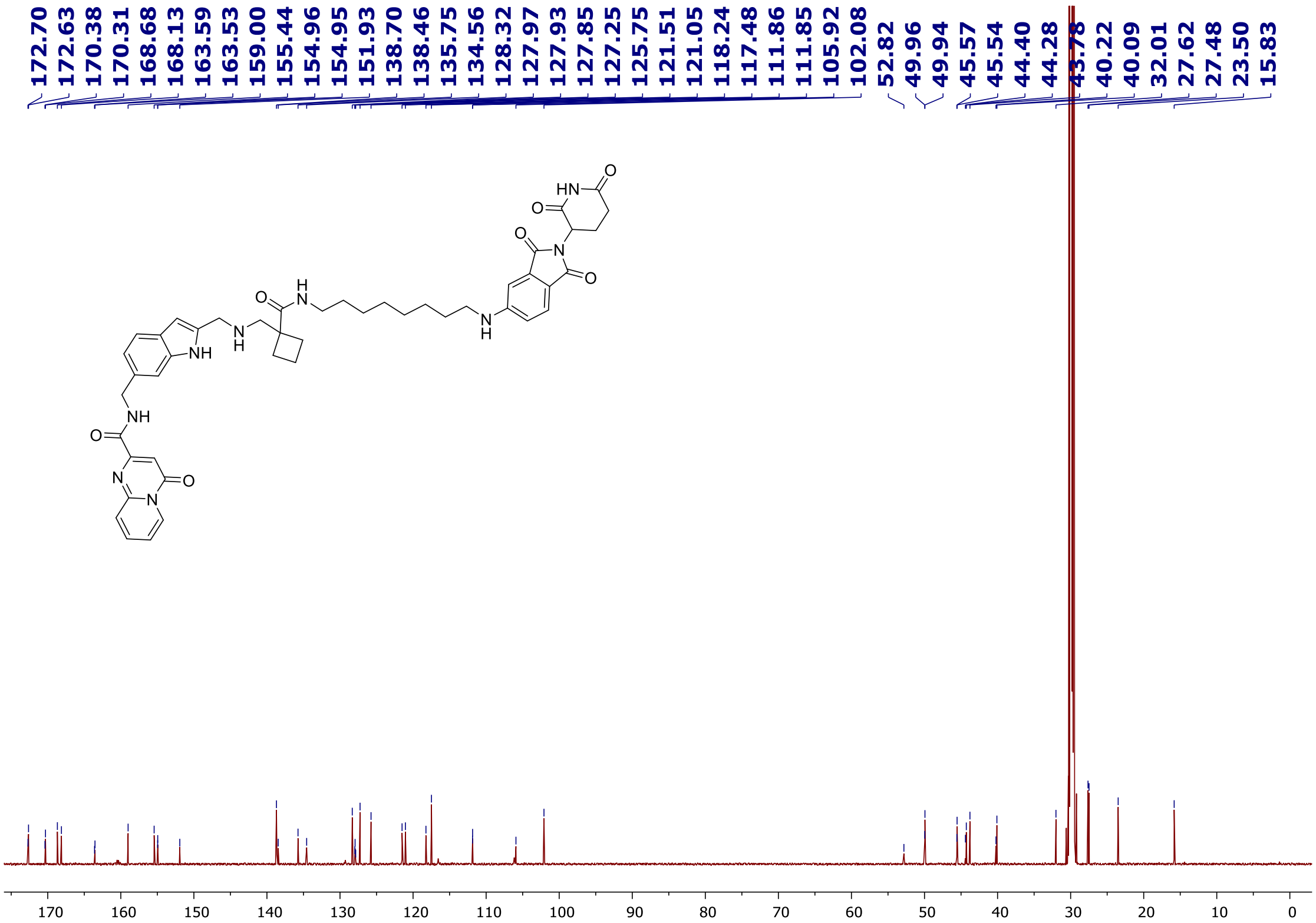

^{1}H NMR Spectrum of **5** (500 MHz, Methanol-d_4):

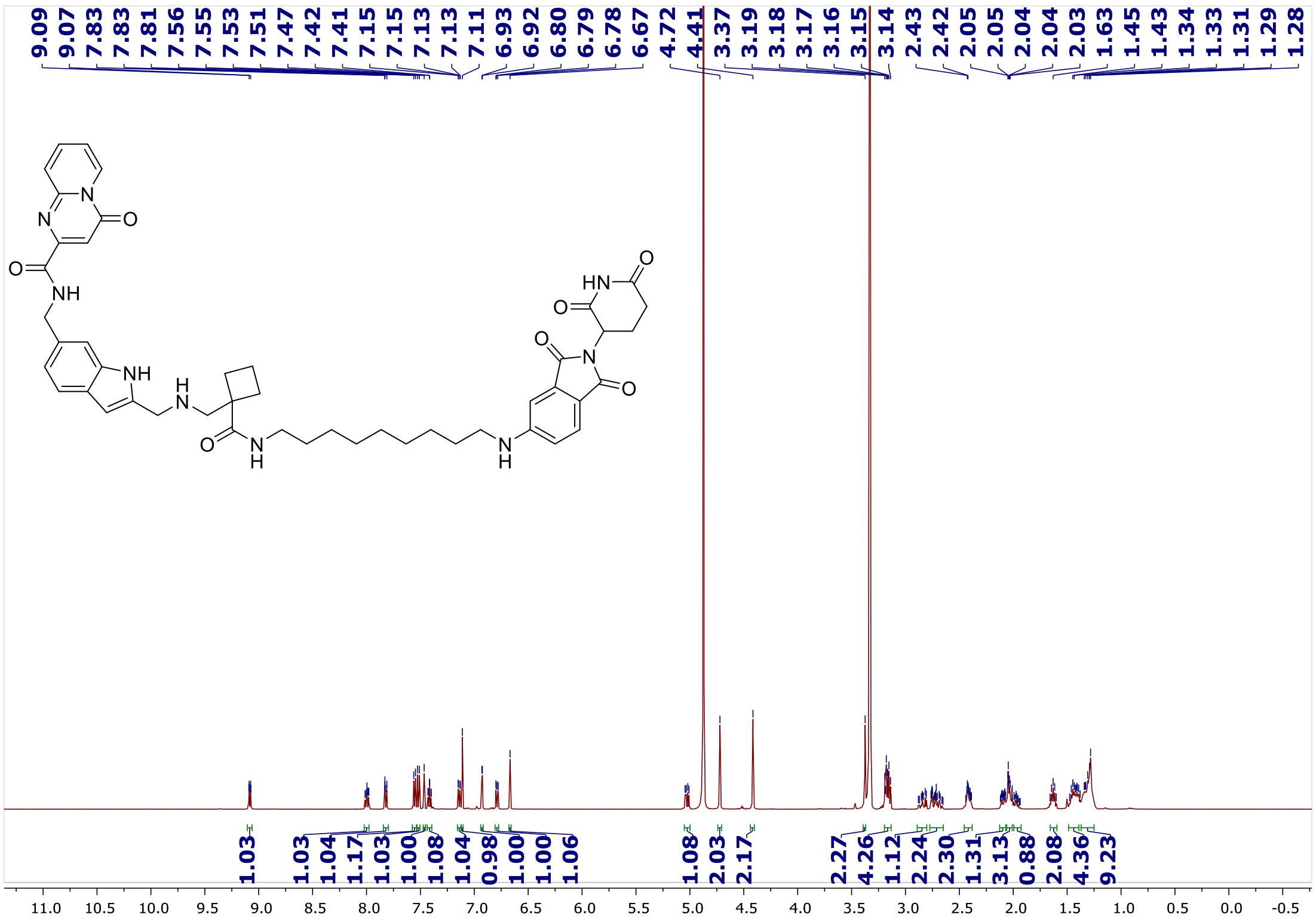

^{1}**H NMR** Spectrum of **6** (700 MHz, Acetone-d_6):

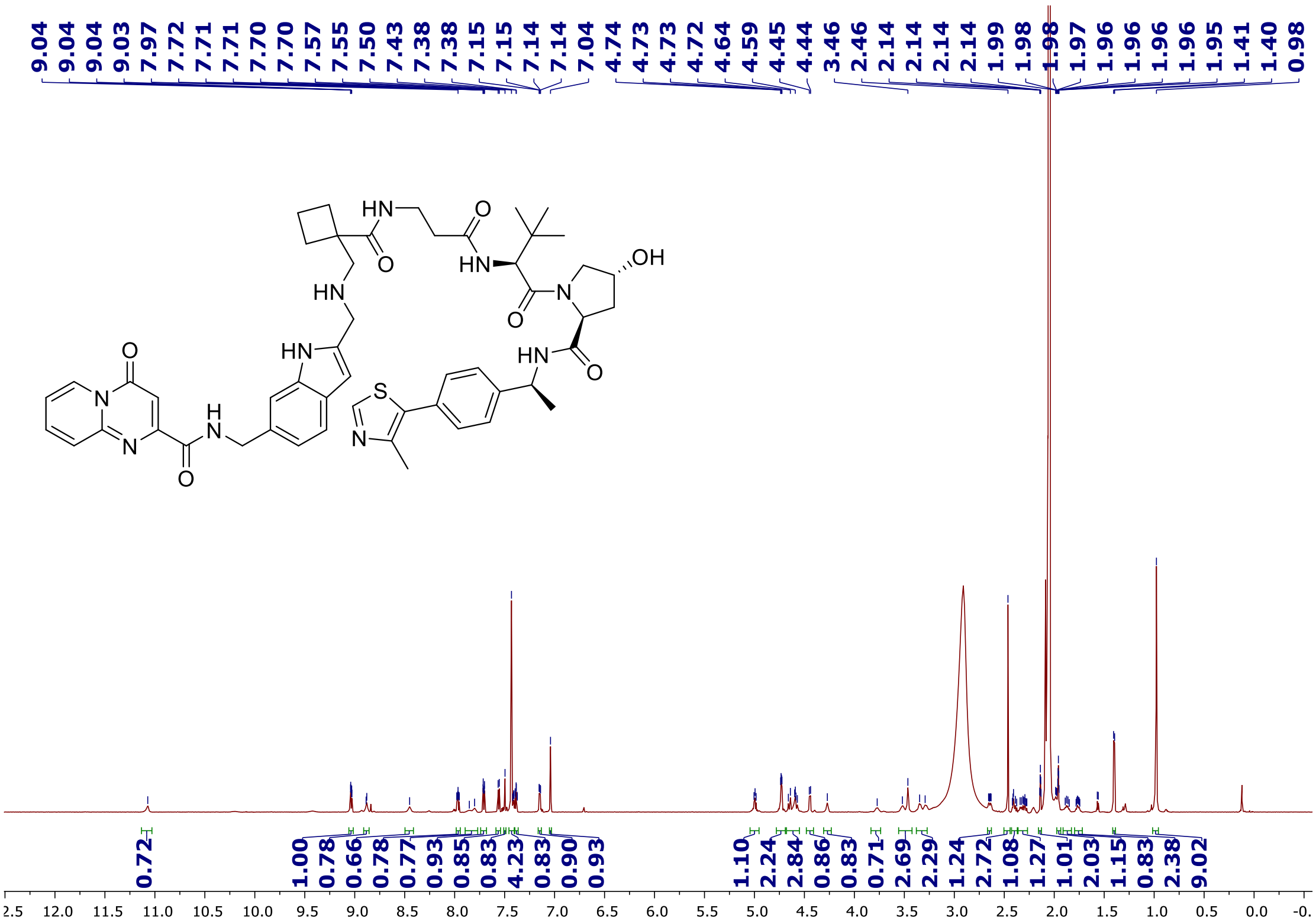

^{1}**H NMR** Spectrum of **7** (500 MHz, Chloroform-d):

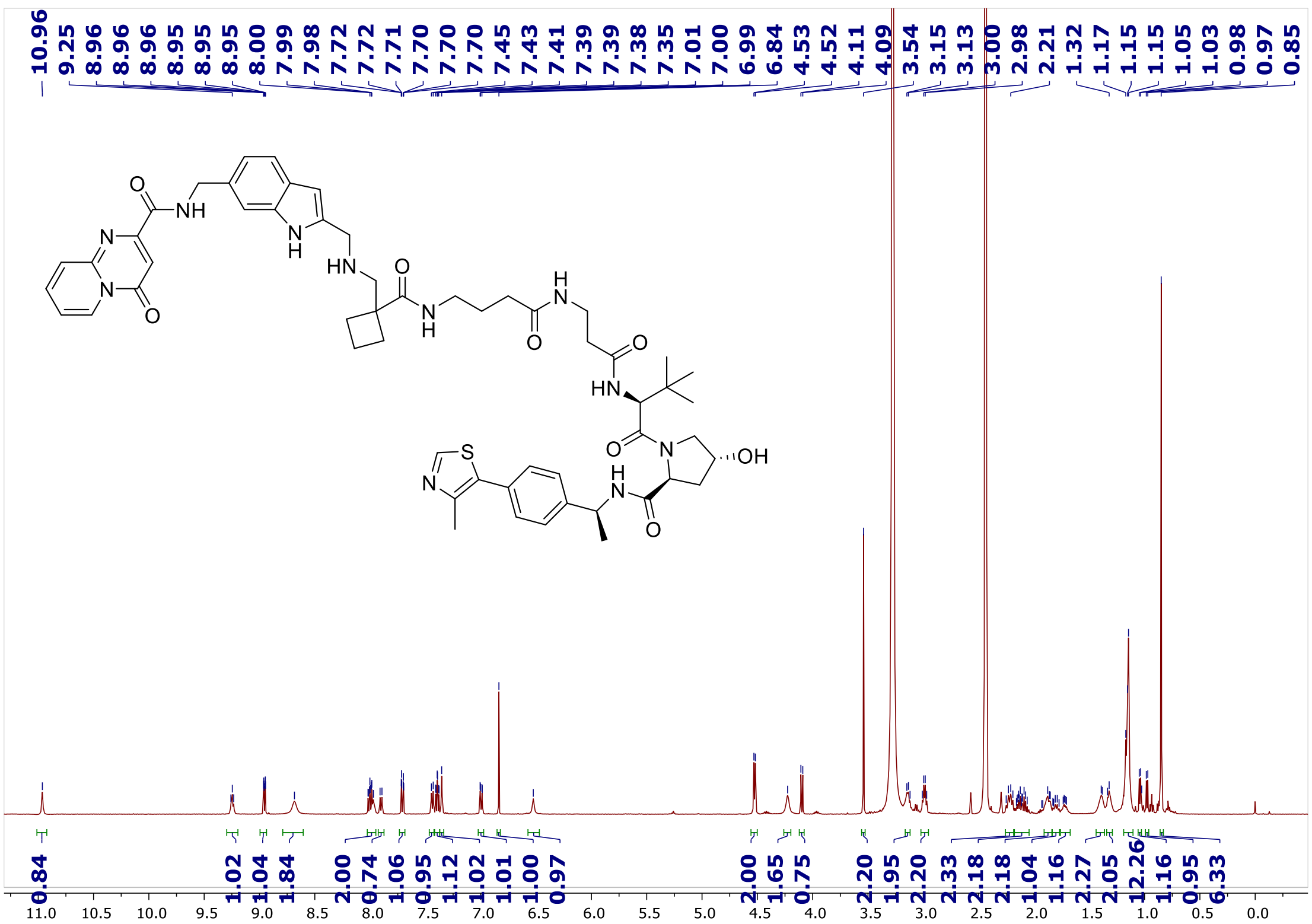

^{1}H NMR Spectrum of **8** (700 MHz, Acetone-d_6):

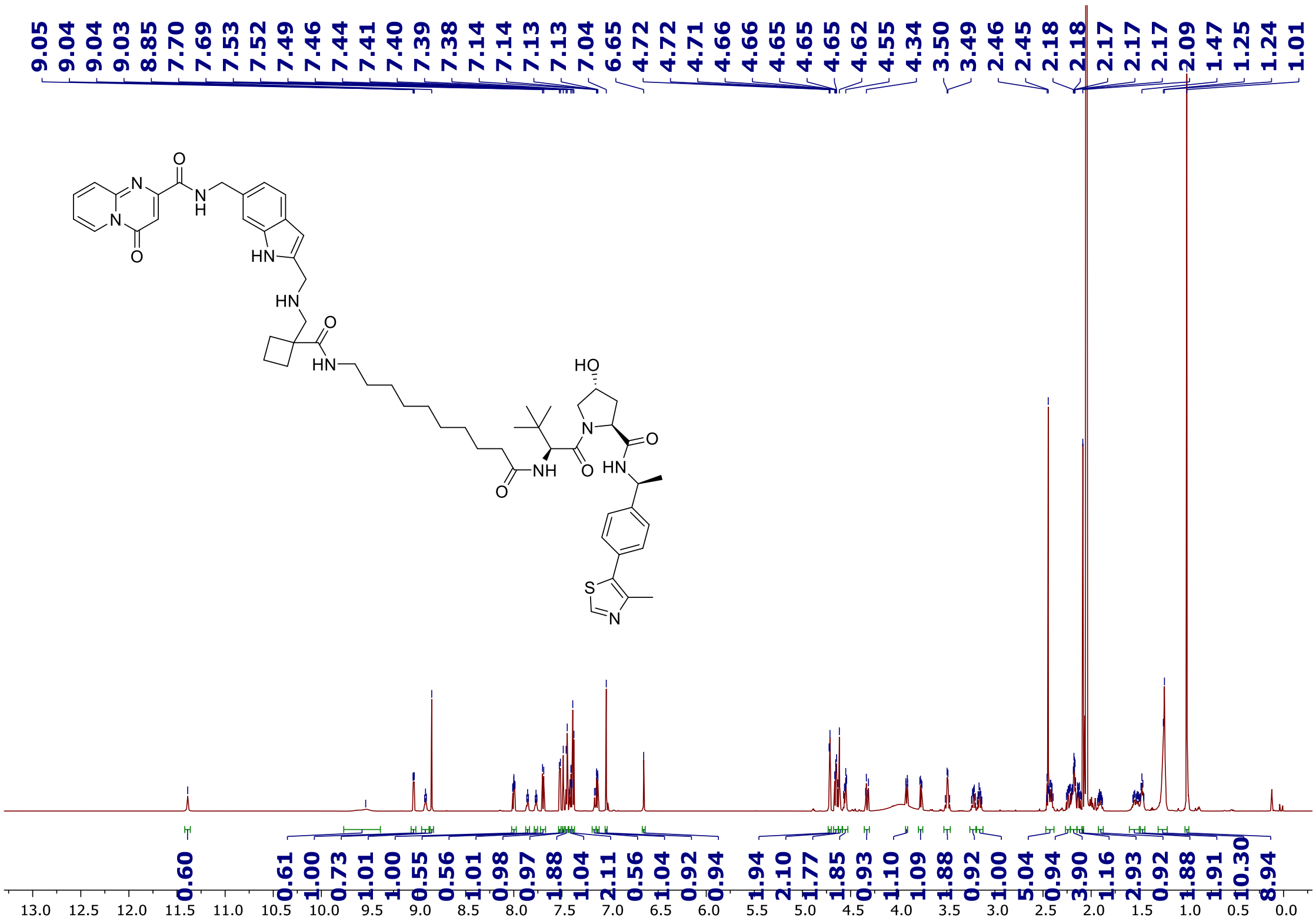

^{13}C NMR Spectrum of **8** (176 MHz, Acetone):

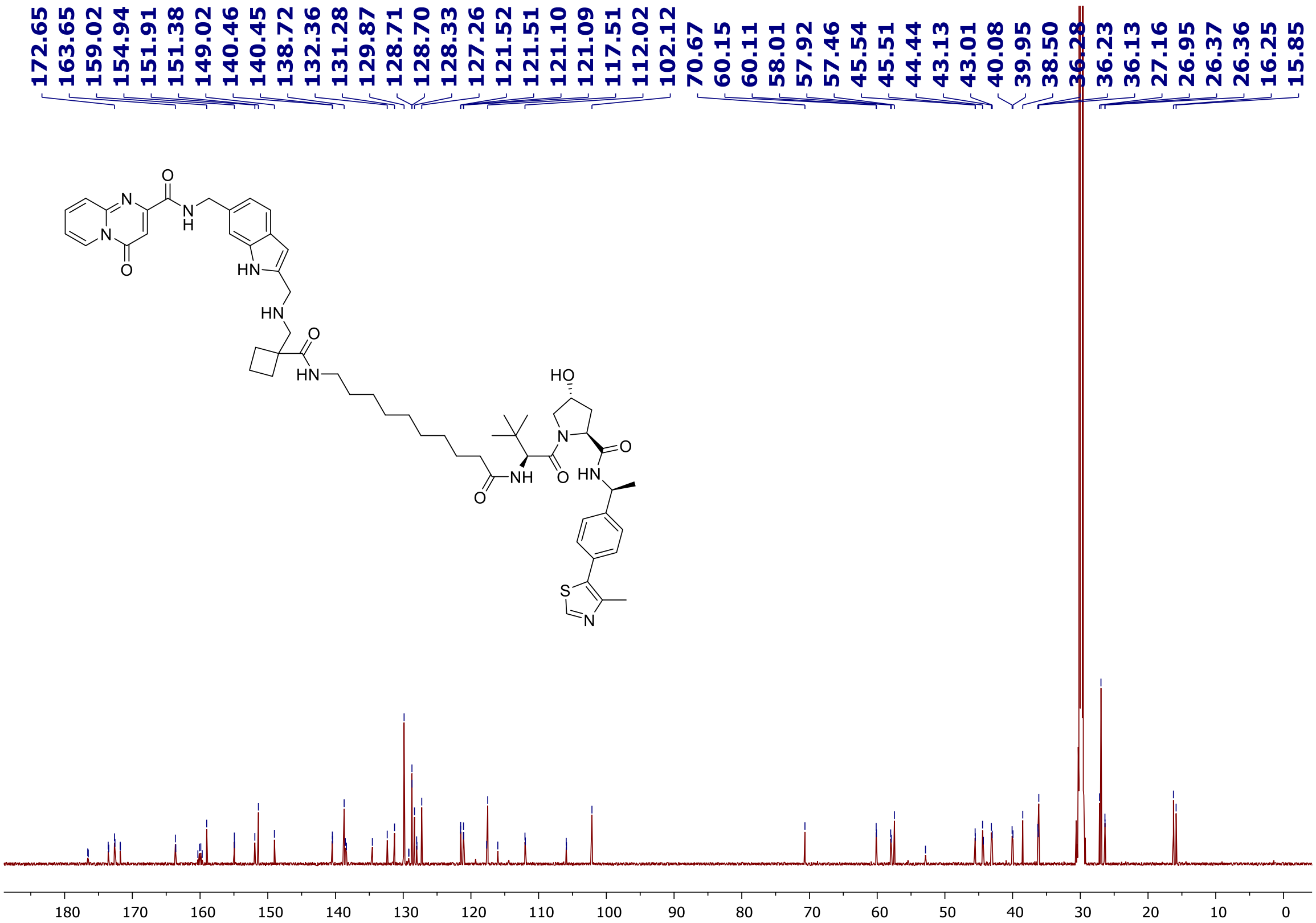

[1] **H NMR** Spectrum of **9** (700 MHz, DMSO-d_6):

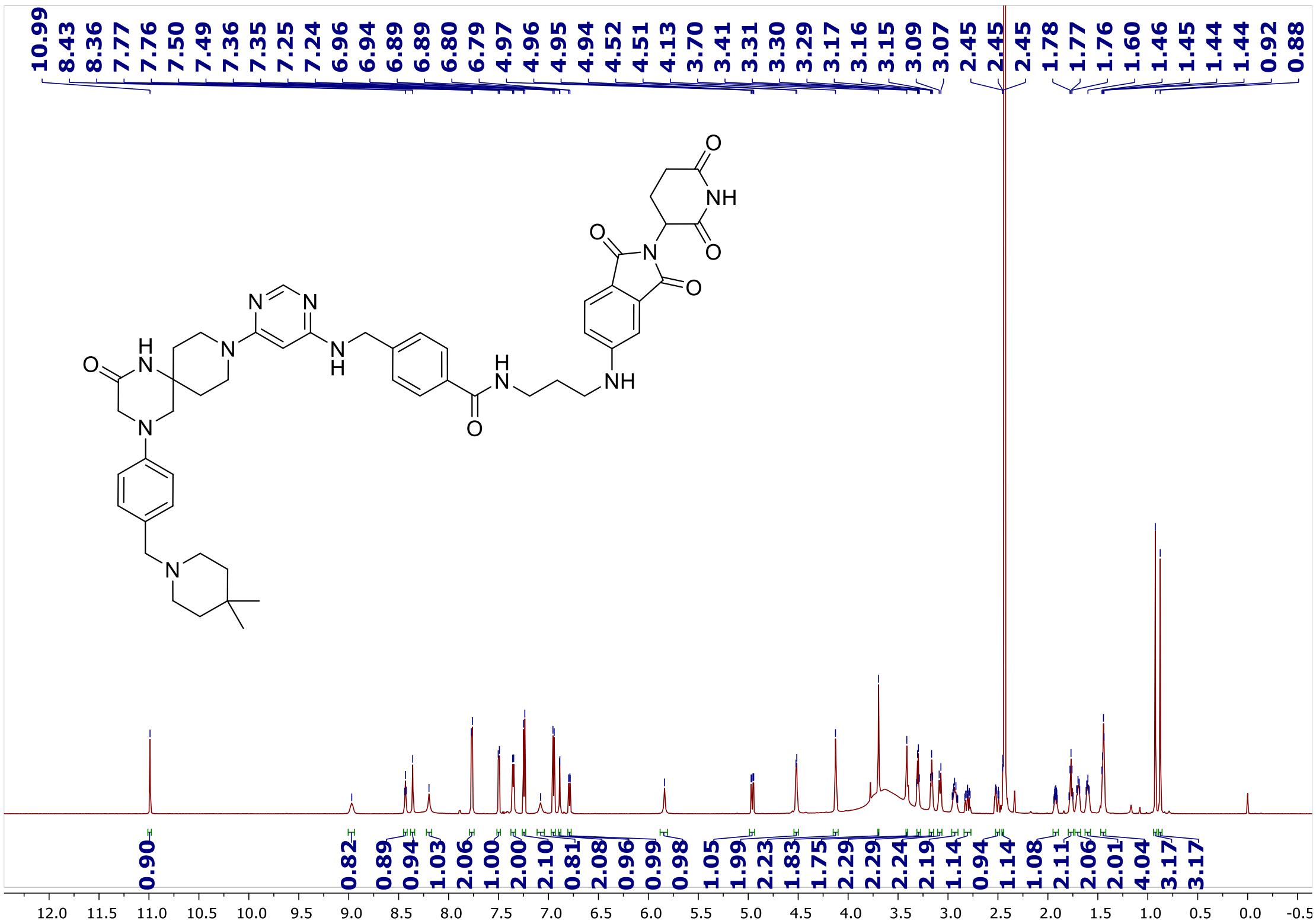

[13] **C NMR** Spectrum of **9** (176 MHz, Methanol-d_4):

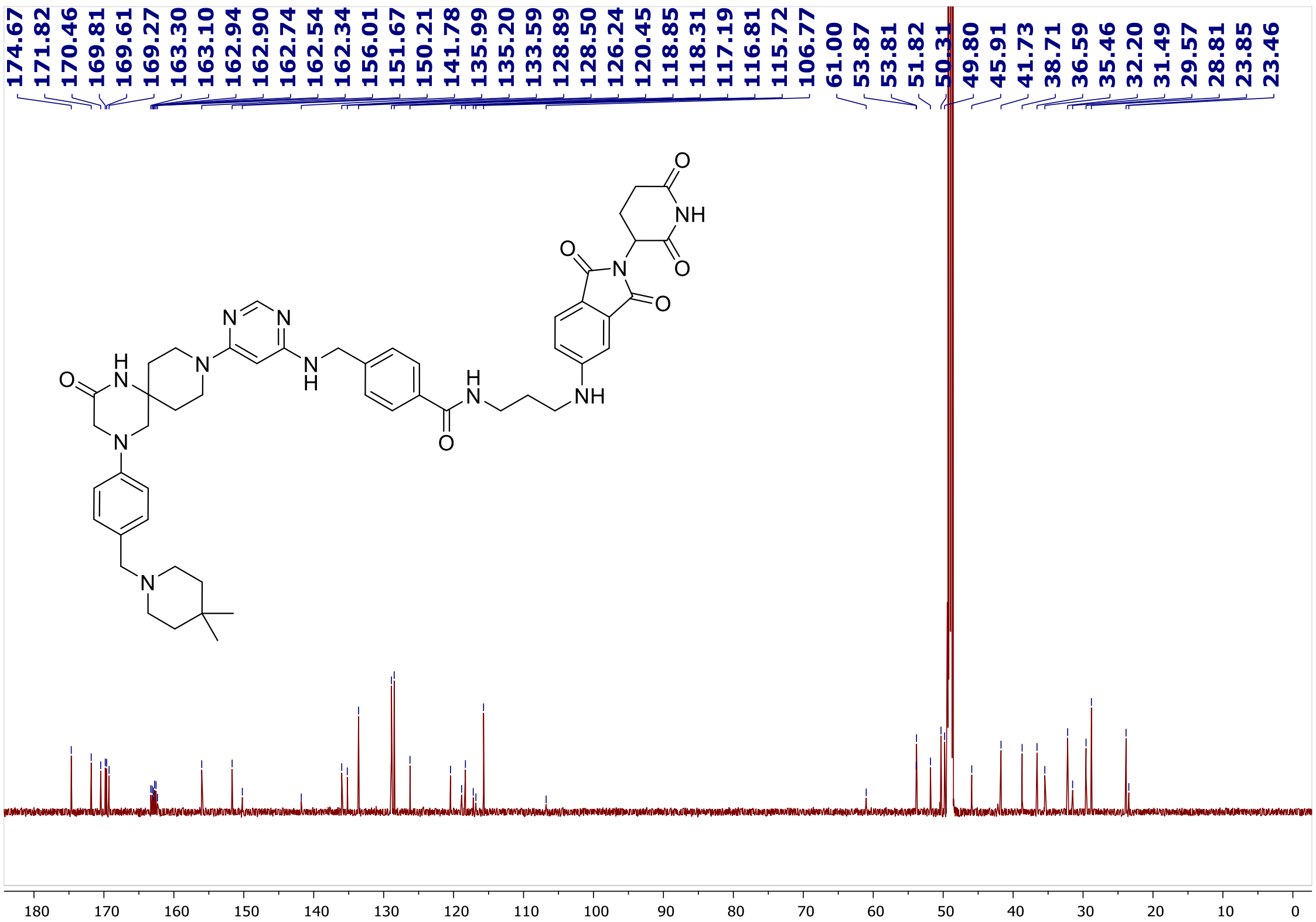

^{1}H NMR Spectrum of **10** (700 MHz, DMSO-d_6):

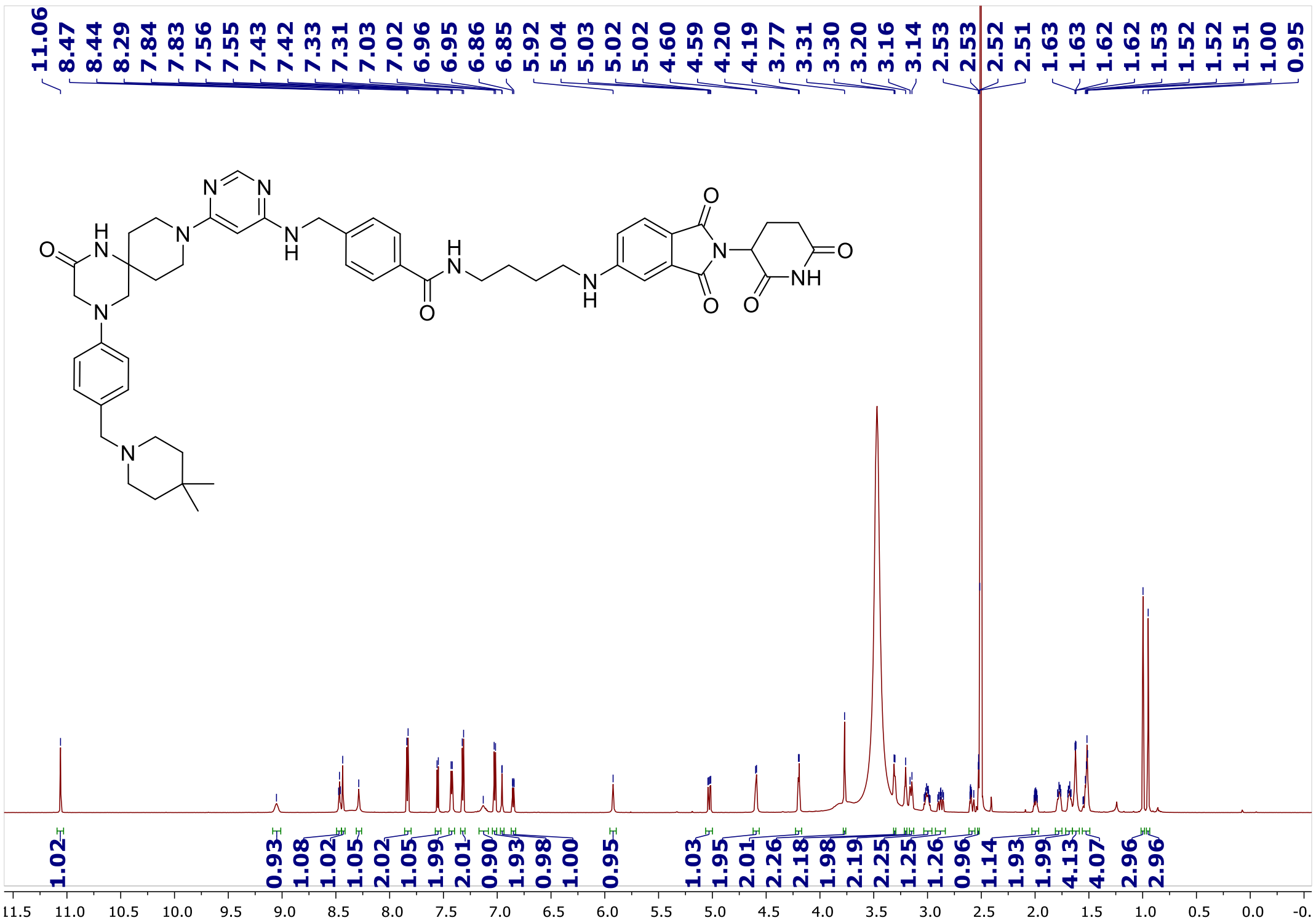

^{13}C NMR Spectrum of **10** (176 MHz, Methanol-d_4):

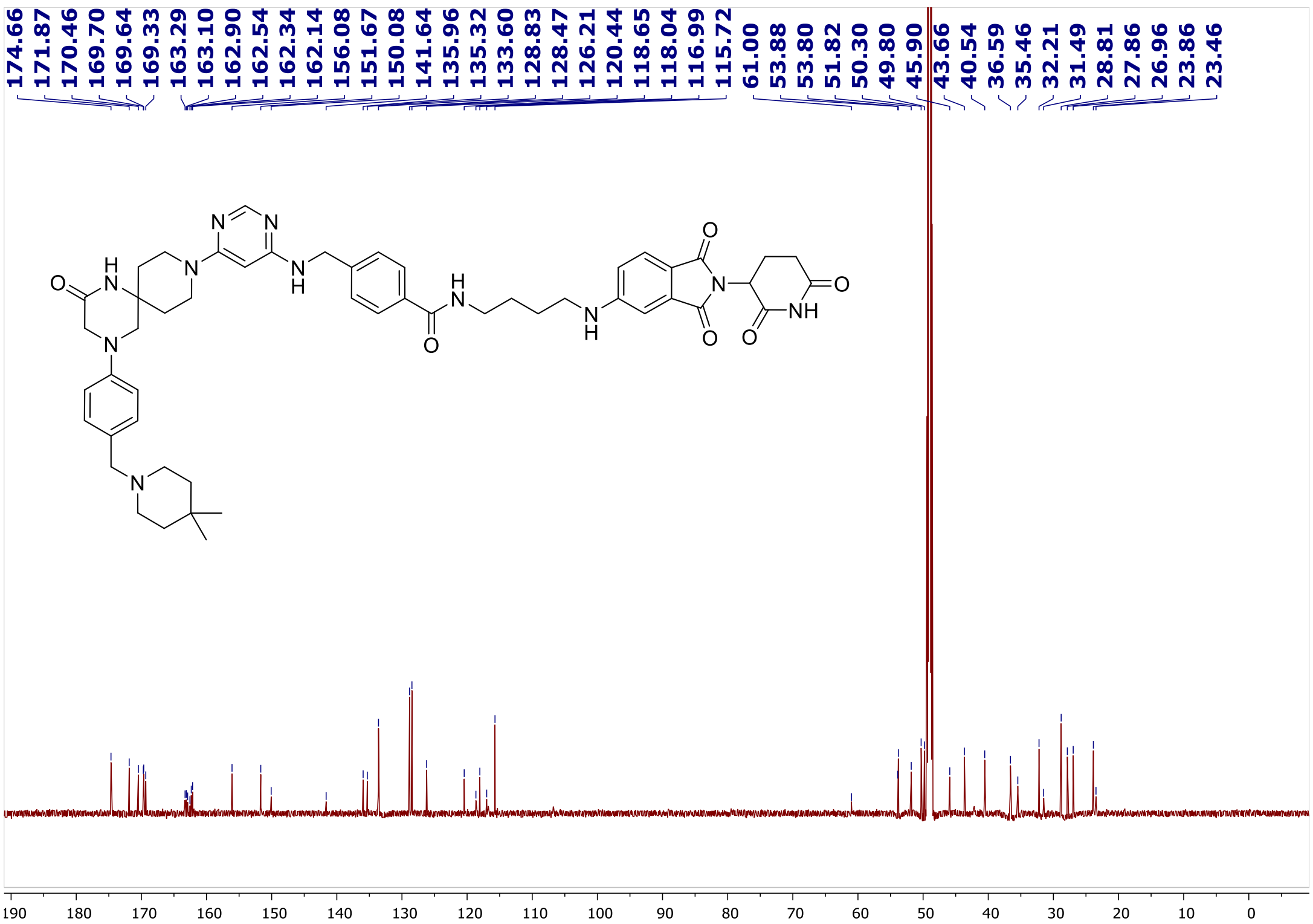

^{1}H **NMR** Spectrum of **11** (700 MHz, DMSO-d_6):

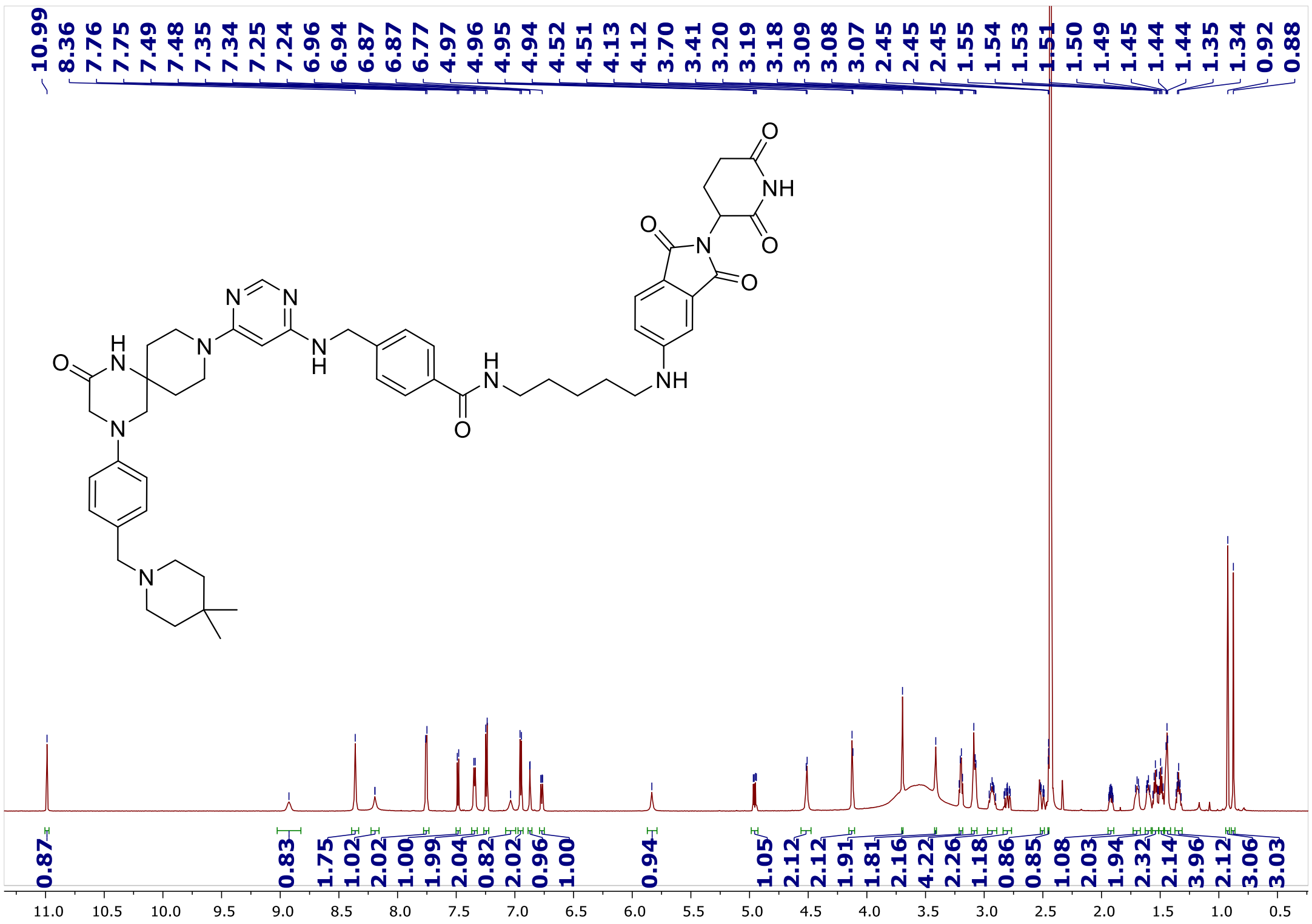

^{1}H **NMR** Spectrum of **12** (700 MHz, Methanol-d_4):

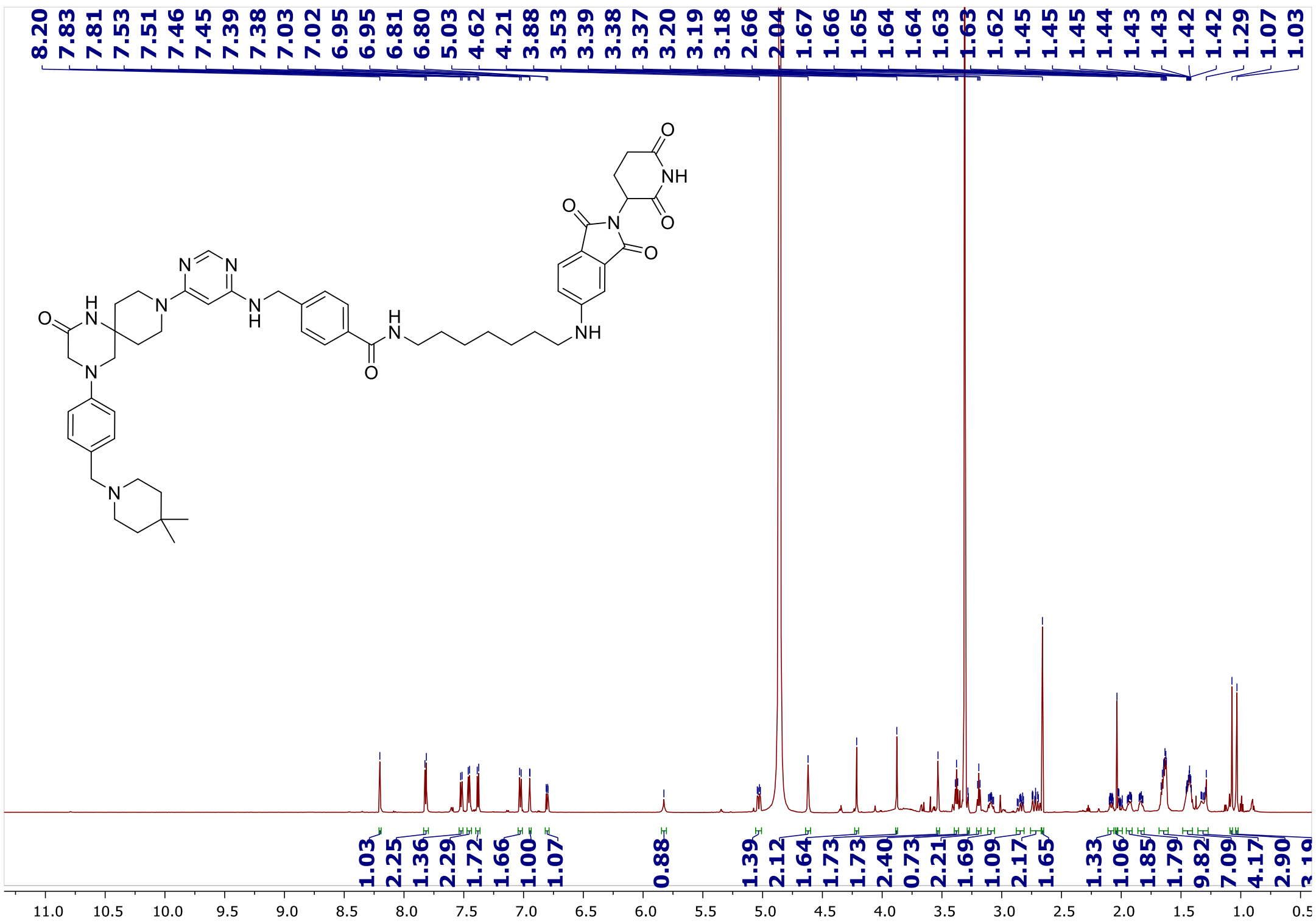

^{13}C NMR Spectrum of **12** (176 MHz, Methanol-d_4):

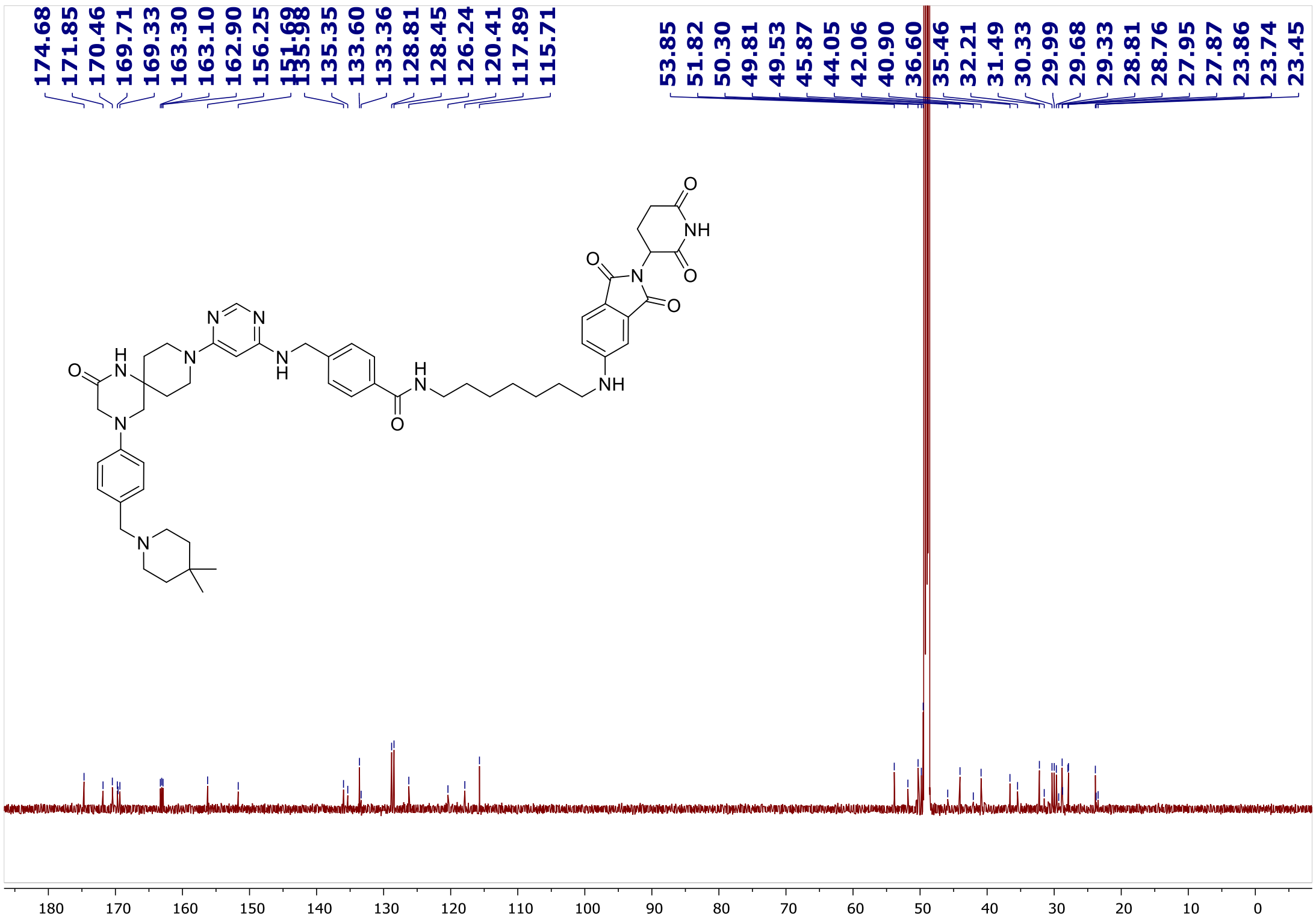

^{1}H NMR Spectrum of **13** (700 MHz, DMSO-d_6):

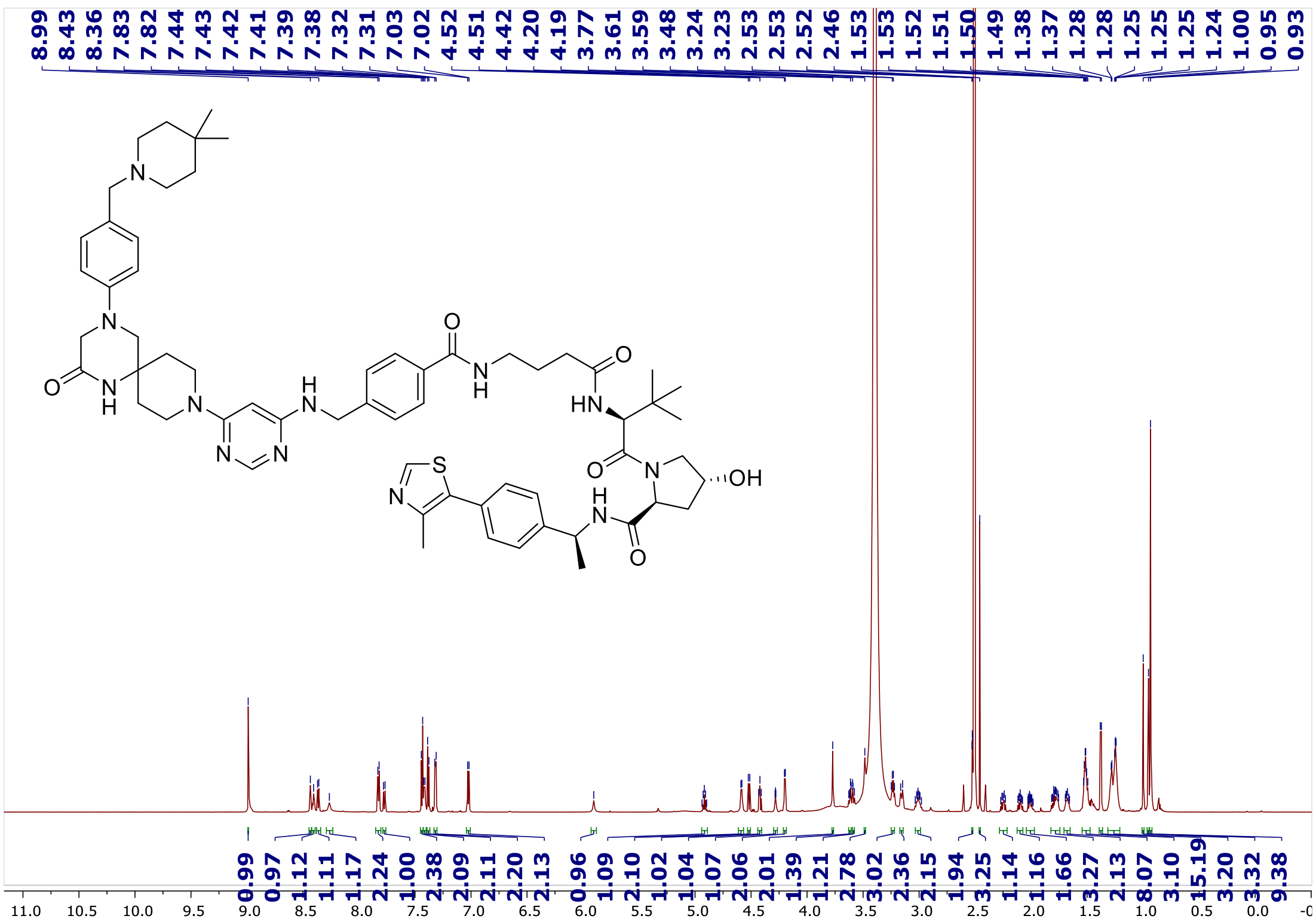

^{13}C NMR Spectrum of **13** (176 MHz, Methanol-d_4):

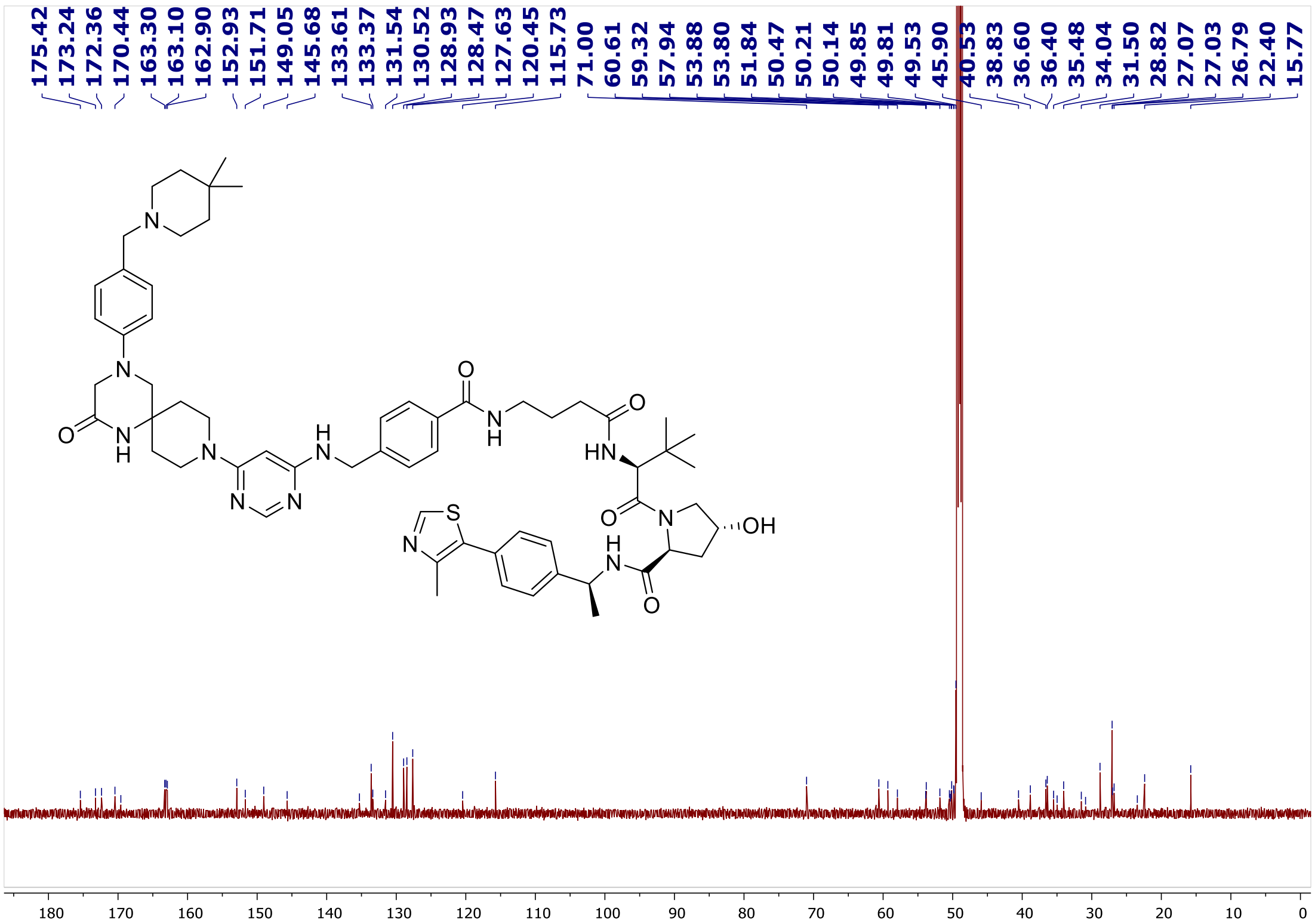

^{1}H NMR Spectrum of **14** (700 MHz, DMSO-d_6):

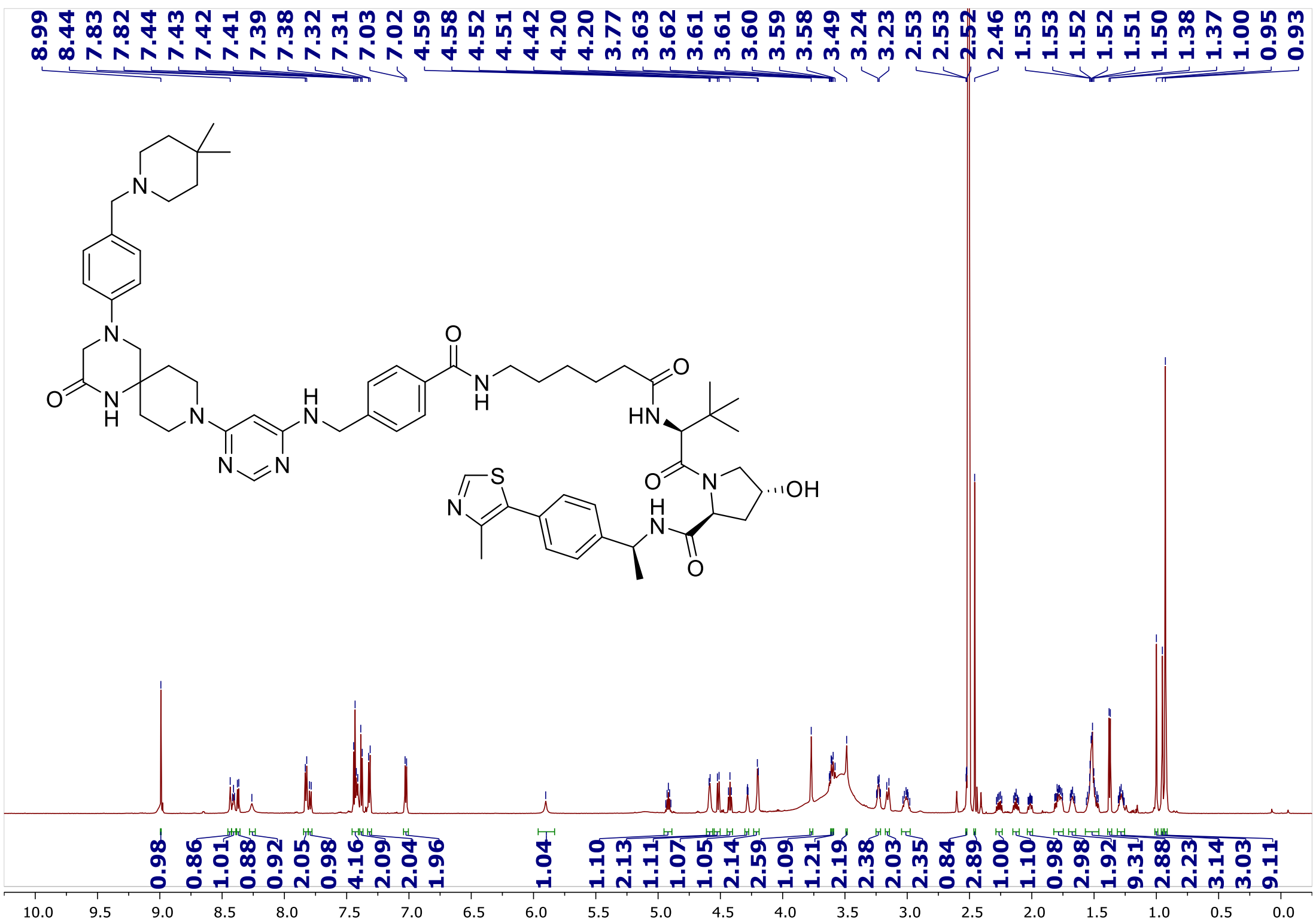

References

(1) Francesco Errani, Annalisa Invernizzi. *et al.* Proteolysis Targeting Chimera Degraders of the METTL3–14 m^6A-RNA Methyltransferase. ***JACS Au*** 2024 *4* (2), 713-729.

(2) Yankova, E., Blackaby, W., Albertella, M. *et al.* Small-molecule inhibition of METTL3 as a strategy against myeloid leukaemia. ***Nature*** 2021 593, 597–601.
